# The Role of –OEt Substituents in Molybdenum-Assisted Pentathiepine Formation—Access to Diversely Functionalized Azines

**DOI:** 10.3390/molecules29163806

**Published:** 2024-08-11

**Authors:** Roberto Tallarita, Lukas M. Jacobsen, Siva S. M. Bandaru, Benedict J. Elvers, Carola Schulzke

**Affiliations:** 1 Institute of Biochemistry, Bioinorganic Chemistry, University of Greifswald, Felix-Hausdorff-Str. 4, 17489 Greifswald, Germany; roberto.tallarita@uni-greifswald.de (R.T.); lukasmanuel.jacobsen@stud.uni-greifswald.de (L.M.J.); siva.bandaru@uni-greifswald.de (S.S.M.B.); benedic@umich.edu (B.J.E.); 2 Department of Chemistry, University of Michigan, Ann Arbor, MI 48109-1055, USA

**Keywords:** pentathiepines, molybdenum, polysulfides, *N*-heterocycles, *S*-heterocycles, indolizines, crystal structure, functionalization

## Abstract

1,2,3,4,5-pentathiepines (PTEs) are naturally occurring polysulfides of increasing scientific interest based on their identified pharmacological activities. Artificial PTEs with *N*-heterocyclic backbones are efficiently synthesized via mediation by a molybdenum–oxo-bistetrasulfido complex. A common feature of all precursor alkynes successfully used to date in this reaction is the presence of a –CH(OEt)_2_ group since the previously postulated mechanism requires the presence of one OEt^–^ as the leaving group, and the second must become a transient ethoxonium moiety. This raised the question of whether there really is a need for two, maybe only one, or possibly even zero ethoxy substituents. This research problem was systematically addressed by respective variations in the precursor-alkyne derivatives and by employing one related allene species. It was found that the total absence of ethoxy substituents prevents the formation of PTEs entirely, while the presence of a single ethoxy group results in the possibility to distinctly functionalize the position on the resulting *N*-heterocyclic pyrrole five ring in the target compound. This position was previously exclusively occupied by an –OEt for all products of the molybdenum-mediated reaction. The allene was applied with similar success as precursor as with the related alkyne. The now-employable significant change in precursor composition gives access to a whole new PTE subfamily, allowing further modulation of (physico)-chemical properties such as solubility, and provides additional insight into the mechanism of PTE formation; it comprises a merely partial validation of the previous hypothesis. The new alkyne precursors and pentathiepines were characterized by a variety of instrumental analyses (NMR, mass spec, UV–vis) and in six cases (one alkyne precursor, one unexpected side product, and four PTEs) by single-crystal X-ray diffraction. Syntheses, isolation procedures, analytical data, and the impact of the findings on the previously proposed mechanism are described in detail herein.

## 1. Introduction

Polysulfide-bearing compounds of marine origin, most notably cyclic polysulfide metabolites, represent a distinctive, relatively uncommon, yet significant category of natural products. Numerous studies have revealed their beneficial biological activities comprising antitumor, antibiotic, anti-inflammatory, and enzyme-inhibitory effects [1]. Peculiar as their chemical structures may look on paper, pentathiepines (PTEs) in general are therefore not only chemically but also pharmacologically quite notable [2,3,4,5,6]. 1,2,3,4,5-pentathiepines are heterocyclic unsaturated polysulfides featuring a seven-membered ring consisting of a chain of five sulfur atoms, the ends of which are tethered together by a –C=C– moiety or, more generally, two sp^2^ hybridized carbon atoms. Simple artificial pentathiepines were first reported in the early 1970s [7]. A significant breakthrough occurred in 1991 with the discovery of these compound types in animals belonging to a group of non-vertebrate marine filter feeders: the *ascidians* or sea squirts [8]. Since then, it has become well known that these animals are quite adept in the production of a variety of sulfur-rich compounds, which include PTEs but also some alkaloids, which are employed as self-defense mechanisms against pathogens [9,10]. Trying to exploit the pentathiepines’ medicinal potential with new synthetic compounds was for a long time based on procedures at relatively or even very harsh conditions [7,11,12,13,14]. In 2013, merely by accident, a new procedure was discovered that facilitates the synthesis of structurally complex *N*-heterocyclic pentathiepines under rather benign conditions [15]. This molybdenum-mediated protocol turned out to be broadly applicable with a tolerance for and compatibility with a large variety of functional groups in the starting materials [16,17].

The key and, at the same time, limiting factor of this route is the employment of 2-(3,3-diethoxyprop-1-yn-1-yl)azine precursors. These are –CH(OEt)_2_ functionalized *N*-heterocyclic alkynes, which can be easily obtained by Sonogashira coupling reactions [18]. Such a synthon, upon reaction with one equivalent of elemental sulfur (cyclo-S_8_) and half an equivalent of bistetrasulfido(oxo)molybdate(IV) complex ((NEt_4_)_2_[Mo^IV^O(S_4_)_2_]), leads to the formation of PTEs. The products may be distinctly substituted on the *N*-heterocyclic six-membered ring of the backbone. However, to date, every single one of the reported respective products from the molybdenum-mediated synthetic procedures exclusively bears an ethoxy group on the pyrrolic five-membered central ring.

**Figure 1:**
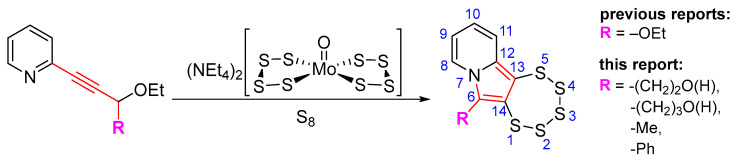
General reaction scheme for the synthesis of 1,2,3,4,5-pentathiepino[6,7-a]indolizines, from diversely substituted 2-(3-ethoxyprop-1-yn-1-yl)pyridines. In all previous reports: R = –OEt; in this report: R = –Me, –Ph, –CH_2_CH_2_OH, and –CH_2_CH_2_CH_2_OH. The blue digits represent the atom numbering scheme for this type of tricyclic pentathiepine.

In this work, we systematically investigated the necessity of the presence of two, one, or zero –OEt substituents on the precursor alkynes for PTE formation. Considering the, by now, vast experience we have with this type of chemistry, we hypothesized that the crucial event in PTE formation is the detachment of one alcoholate monoanion, which thus comprises an essential functional group, while the second crucial event is the formation of a cationic moiety adjacent to the alkyne, which in all likelihood might not have to be limited to an ethoxonium species. We thereby discovered access to the functionalization of the pyrrolic central ring with a variety of substituents, which will also facilitate the future additional targeted modulation of the chemical and electronic structures as well as their (physico-)chemical properties, such as solubility or stability (Figure 1). In order to evaluate the previously proposed mechanism postulated in the primal work of Zubair et al. [15] with regard to molybdenum-mediated PTE formation, for the first time, the alkyne precursor was derivatized from the as of yet omnipresent –CH(OEt)_2_ group. This, consequently, results in a new PTE subfamily with its unprecedented varied functionalization on the central pyrrolic ring.

Aside from supporting the initial and likely most crucial step in the postulated mechanism of molybdenum-mediated PTE formation, these findings will also broaden the scope for drug design and potential therapeutic applications [19]. All newly synthesized pentathiepines were fully characterized spectroscopically/analytically, and some individual molecular structures of precursors, side products, and products were determined with single-crystal X-ray diffraction.

## 2. Results and Discussion

**Figure 2:**
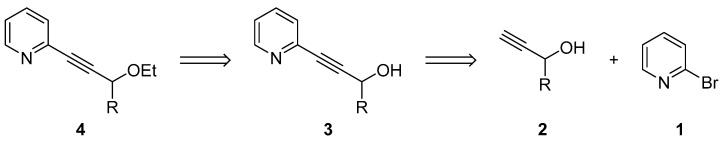
Most feasible general synthetic route for the preparation of the precursor alkynes bearing a single ethoxy substituent; retrosynthetic presentation with precursor numbering pattern to be used throughout.

In order to test the hypothesis, i.e., for the molybdenum-mediated pentathiepine formation, a single –OEt substituent or, more generally, a single aliphatic ether function on the alkyne precursor should be sufficient, respective suitable starting materials had to be developed. This required the simultaneous presence of one –OEt moiety and of a distinct substituent on the tertiary propargyl carbon. To this end, the most suitable sequence of transformations had to be identified, for which various general approaches for their synthesis were investigated, adapted, and refined. The generation of the ether, functioning as the very last step of precursor synthesis, turned out to be the most feasible pathway (Figure 2).

In addition to these precursors, alkyne species were also used in typical PTE syntheses without bearing any ether function: those with cyclic di-ether functions and even one allene compound. This was performed in order to obtain evidence for or against the suggested mechanism of molybdenum-mediated PTE formation, i.e., to (at least partially) validate the mechanism that was postulated already in 2013 [15].

### 2.1. Testing the Postulated Mechanism—An Overview

**Figure 3:**
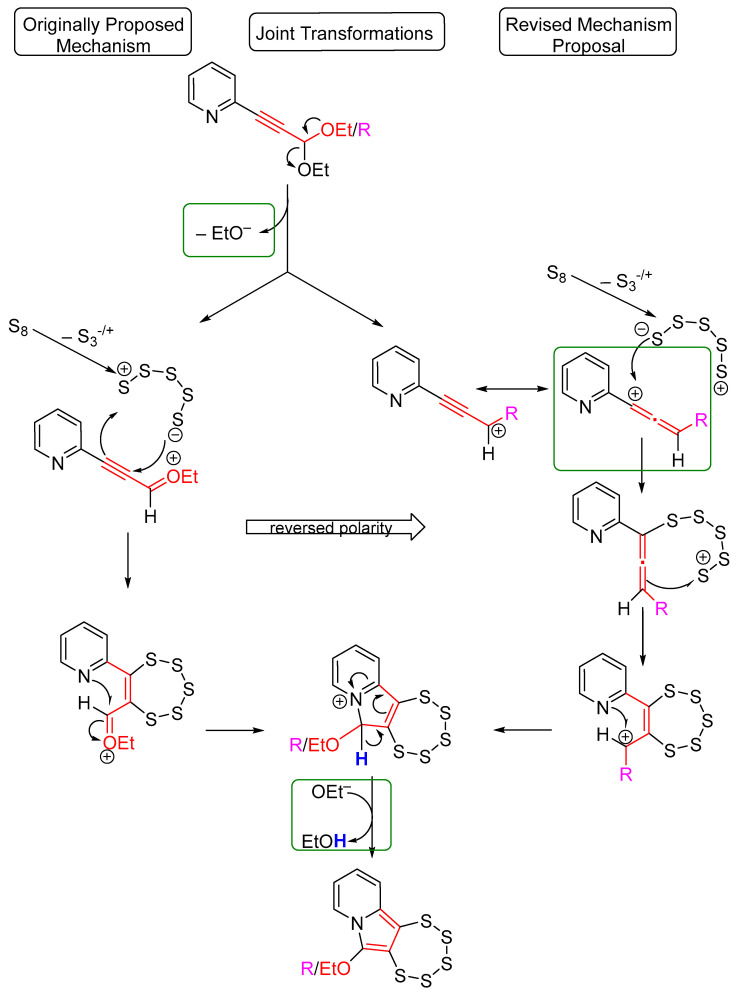
Proposed reaction mechanism(s) for the molybdenum-mediated pentathiepine formation. To the left: the originally proposed transformations [15]; to the right: those we consider more likely based on the observations described below. In the green boxes, events are shown that are strongly supported by the study presented here.

It was originally hypothesized that the first step in the molybdenum-mediated pentathiepine synthesis involves the elimination of one negatively charged OEt^–^ group from the acetal, resulting in the formation of an ethoxonium ion (R_2_C=O^+^Et) [15] (see Figure 3 for a comparison of old and newly considered mechanistic proposals). The OEt^−^ abstraction may be facilitated by interaction with the molybdate complex, but to date, that is not supported by any evidence. The C≡C triple bond in subsequence was proposed to react with a pentasulfido zwitterion (^−^S··S_3_··S^+^) generated from S_8_ with the help of the molybdate complex’s tetrasulfido ligands. A polarization of the triple bond was presumed due to the presence of the positively charged ethoxonium moiety. However, it appears similarly likely that the immediately formed propargylcation engages in resonance in the opposite direction, i.e., towards the alkyne moiety instead of moving the positive charge to the oxygen. Such a cation is typically stabilized via an allene resonance structure, which would reverse the polarity of the attack of the S_5_^+/−^ moiety. Since the sulfur fragment to be attached is symmetrical, the outcome will be the same either way. Considering all this, the presence of an ethoxonium may not be necessary per se according to the revised hypothetical mechanism. Upon the formation of the pentathiepine ring, a rearrangement of the delocalized electrons was anticipated to facilitate the nucleophilic attack of the aza group’s nitrogen on the carbonyl moiety. This may, in fact, be an even more imminent interaction with a carbocation. Then, proton loss occurs, followed by re-aromatization of the newly formed tricyclic skeleton in either of the two mechanisms. Besides the *N*-heterocyclic pentathiepine, an actual ethanol molecule results from this reaction [15].

One result of the study by Zubair et al. was confirmation of the impossibility to generate a pentathiepine from an alcohol-modified precursor alkyne (propargylic alcohol) devoid of any –OEt group. We tested the analogous precursor **3a**, which is a pyridine rather than a pyrazine species, as in said reference [15], under typical reaction conditions for PTE formation. As anticipated, the reaction towards the PTE, which was repeatedly carried out, did not yield any product whatsoever (Table 1). Similar results were obtained with the propyl-substituted precursor **4′a**. (The prime is applied to the label since the compound does not bear an ethoxy substituent, as shown in Figure 2 above.) This confirms the previous observation that in the absence of –OEt, the reaction does not occur, and it also supports the elimination of the alcoholate anion to be an early, if not the very first, and a truly crucial step of molybdenum-mediated pentathiepine formation.

We next looked at alkyne precursor species with only one ethoxy substituent, the first one being **4b**. In this case, the –OEt-bearing carbon was also a methylene, as in **3a** and **4′a**. Not surprisingly, the reactivity here is also notably modified from previous reports since the –CH(OEt)_2_ group is in fact a di-acetal protected aldehyde, while the –CH_2_(OEt) in **4b** is an ether. Still, the adjacency to a triple bond and potential conjugation to an aromatic system may support removal of the OEt^–^ group and stabilize the subsequent intermediate. In case of **4b**, this would result in a primary carbenium cation, which has a substantially high reactivity. While a pentathiepine product was indeed found, it was, quite surprisingly, the –OEt-substituted one, namely **5b** (confirmed crystallographically), and it was formed only in traces. We propose that in an intermolecular interaction of precursors and/or transient intermediates, it is likely that one –OEt is transferred onto an activated precursor, and from this, the reaction proceeds as typical. However, due to the high reactivity of the intermediate carbenium cation, this scenario takes place only for a negligible number of reactants, and the resulting reaction mixture contains a considerably broad variety of side products, which could not be isolated or characterized. Employing a methylene carbon as the one to become part of the pyrrolic 5-ring system therefore appears unsuitable.

Following this, the aforementioned position was substituted by a methyl (**4c**) and a phenyl group (**4d**). Here, the transient species during the reaction are secondary carbenium cations, which are significantly more stable than the primary ones. Supposedly, the lifespans of these species during PTE formation are longer compared to the primary one by an extent that allows the reaction to proceed as normal and thereby to outrun unwanted side reactions and/or spontaneous decomposition [20,21]. The small and electron-donating methyl group was employed to explore changes in the reactivity without significantly altering the molecular size. In contrast, the phenyl group was used to introduce a planar, aromatic system that can engage in π–π interactions and potentially stabilize reactive intermediates through conjugation. Two precursors with cyclic analogs of the acetal-protecting group (**4e** and **4f**) were tested to obtain evidence for the reunion of the two components that are split from the precursors (OEt^−^ and H^+^) according to the proposed mechanism. This does indeed take place, as was proven with these particular experiments. In addition, two very interesting PTEs were thereby accessed, now bearing aliphatically linked free alcohol functions on position C-6, which should alter their solvent preferences quite notably. Finally, the allene variation **4g** was taken into the tests to bring in support for the importance of the delocalization and flexibility of the electron density in the π system, showing that an allene may also be one intermediate form of the reaction mechanism.

All in all, four new PTEs (**5c–f**) were successfully synthesized, all bearing distinct substituents at position C-6, which corresponds to the only available location for functionalization directly adjacent to the poly-sulfur ring. Detailed descriptions of all syntheses are provided in the following sections.

### 2.2. Complete Absence of –OEt

A key test in order to evaluate the significance (or insignificance) of the –OEt substituent was to run a reaction in the complete absence of any –OEt group. Precursor **3a** was readily available, as it was also used as starting material for another derivative, and it is the close pyridine relative of a previously employed pyrazine derivative [15]. All respective reactions towards a pentathiepine failed. In addition to the propargyl derivative, one precursor alkyne was also investigated, which has a non-functionalized substituent at the alkyne. The respective precursor 2-(pent-1-yn-1-yl)pyridine (**4′a**) was synthesized without any complication via a Sonogashira coupling reaction between 2-bromopyridine **1** and 1-pentyne. Employing this precursor, however, did not facilitate the isolation or identification of any product resembling a pentathiepine; i.e., the absence of –OEt prevents the formation of the pentathiepine via the molybdenum-mediated pathway, providing direct evidence for the importance of OEt^−^ elimination as a crucial transformation in the reaction sequence.

### 2.3. Presence of a Single –OEt

The simplest version of the alkyne precursor with a single –OEt substituent is the one with the ethoxy residing on a methylene carbon. Obtaining precursor compound **4b**, in contrast to the others, was synthetically relatively straightforward. It involves comparably few synthetic steps due to the commercial availability of the propargyl alcohol starting material. A Sonogashira coupling reaction of 2-bromopyridine **1** with propargyl alcohol led to the desired product with satisfying yields. This was followed by an etherification via the Williamson procedure to yield **4b** [22]. However, optimizing compound purification proved to be complex and laborious. The synthesized species were not bench-stable. Isolation required multiple chromatographic columns, and these most often needed to be run on gradient due to the high polarity. The thermal stability turned out to be a key issue, as intermediate **3a** is unstable above 40 °C.

**Figure 4:**
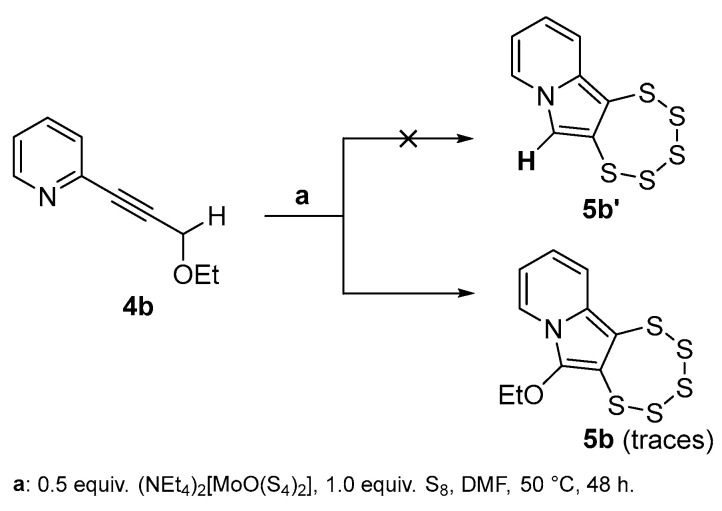
Expected (**5b’**) and actual (**5b**, only traces found) reaction products employing precursor **4b**.

Precursor alkyne **4b** was then tested for pentathiepine synthesis. In contrast to what was observed in reality, a pentathiepine with a hydrogen attached to position C-6 (**5b’**) was expected to result from this trial (Figure 4). Employing **4b** resulted in an undefined mixture of products, which was impossible to isolate and characterize comprehensively. In a proof-of-principle test reaction, a modest amount of a substance in the characteristic yellow PTE color was collected after column chromatography. However, when dissolving it in dichloromethane (DCM), which is part of the typical work-up process, this led to an abrupt color change of the solution to green. A green coloration observed in these reactions was already noted in the past to be a sign of PTE degradation [2,16,17]. In subsequent experiments, carefully avoiding any contact with DCM allowed the isolation of trace amounts of compound **5b**, which was characterized by single-crystal X-ray diffraction (SCXRD) from the very few crystals grown. Its molecular structure was already reported previously [16].

The trace detection of **5b** does not invalidate the hypothesis that the elimination of OEt^−^ is a crucial event during pentathiepine formation. As stated above the simultaneous presence of the activated precursor bearing a primary carbenium cation, free ethanolate and –OEt groups in adjacent activated precursors may well result in the generation of a small portion of intermediate that rearranges eventually to PTE **5b**. The fact that this is found only in traces rather supports the too-high reactivity of transient primary carbenium cation intermediates.

**Figure 5:**
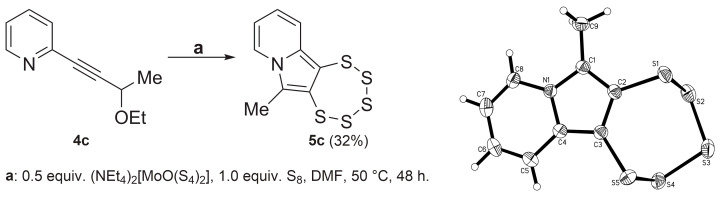
(**Left**): Synthesis of **5c** from **4c** via the molybdenum-mediated pathway providing access to the first indolizine derived PTE lacking the usual –OEt on C-6. (**Right**): Molecular structure of **5c**. Ellipsoids are shown at the 50% probability level.

With regard to a methyl group being attached to position C-6 in the resultant PTE, the synthesis of the starting material **4c** followed the same concept as for **4b**. 2-Bromopyridine **1** and 3-butyn-2-ol **3c** were reacted in a Sonogashira coupling reaction, followed by etherification with EtI. Remarkably, the use of compound **4c** as precursor led to the formation of the first pentathiepine **5c** bearing a methyl group as a new substituent, which replaces the otherwise omnipresent –OEt in position C-6 (Figure 5).

**Figure 6:**
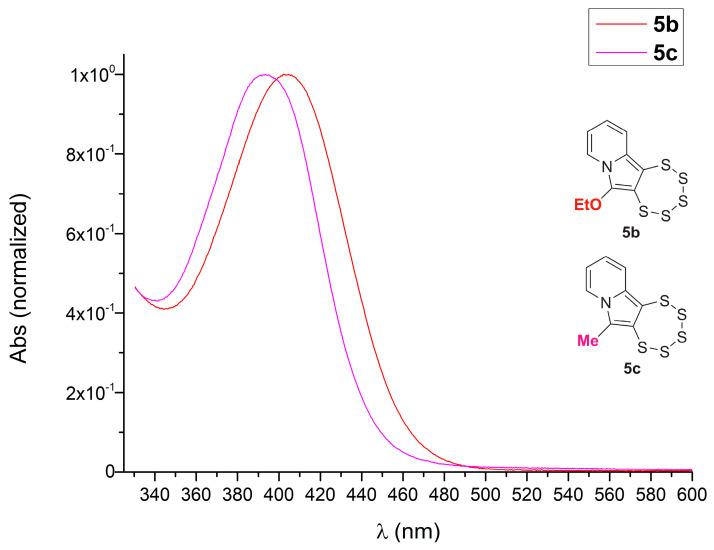
UV–vis spectrum of **5c** compared to the reported spectrum of **5b** (both with normalized absorbance) showing that replacing an ethoxy by a methyl substituent has only a moderate impact on the frontier orbitals (i.e., the molecule’s electronic structure).

The product exhibits a typical bright yellow-orange hue, consistent with other pentathiepine derivatives (UV–vis absorption maxima appear typically around 405 nm), with a maximum absorption in the UV–vis spectrum of 393.5 nm (Figure 6). The replacement of the –OEt substituent by a –CH_3_ substituent therefore induces a blue shift of approximately 10 nm, which does not change the coloration of the compound much but is consistent with a subtle change in the electronic structure. The peak shape, on the other hand, is essentially identical to those in previously recorded UV–vis spectra. It was recently established [16] that this absorption of **5b** and closely related compounds (all with an –OEt on C-6) is due to an excitation from HOMO to LUMO. The HOMO has partial oxygen character in the investigated molecules, while the LUMO does not. It can therefore be tentatively concluded that the change in substituents from ethoxy to methyl increases the HOMO–LUMO gap slightly, most likely due to a relative decrease in energy of the HOMO, which widens the gap.

An immediately notable difference between this new PTE and the more classical ones is evident in the ^1^H-NMR spectrum: the normally very characteristic “fingerprint” at around 4.5 ppm attributed to the diastereotopic –CH_2_O– protons is absent in the spectrum of **5c** (see Figure S27). While mass and elemental analyses corroborated the proposed composition, single-crystal X-ray diffraction (SCXRD; Figure 5 and Table S3) unambiguously proved the molecular/chemical structure of this new derivative. The metrical parameters of crystalline **5c** are unremarkable, as they do not deviate from the tight ranges observed in previously published closely related structures [16,17,23].

**Figure 7:**
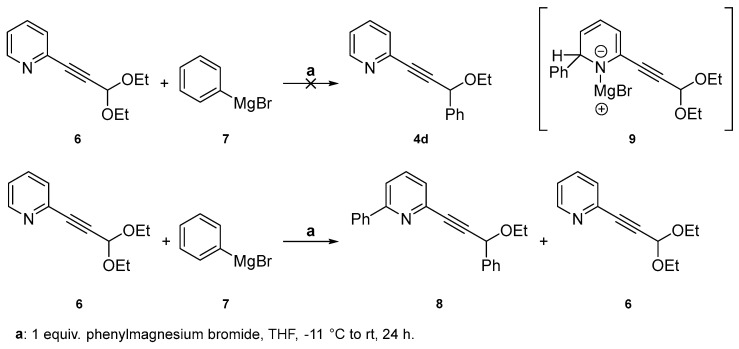
Attempt to replace one –OEt group of **6** by a phenyl substituent leading to **8** instead. (**Top**): hypothesized reaction and product **4d**. (**Bottom**): Actual reaction/product **8** plus unreacted starting material. On the right: The postulated stabilized intermediate **9** likely supporting the formation of **8**.

Finally, a phenyl substituent was introduced at position C-6 in order to install a substituent with a different electronic influence compared to the methyl group and to investigate whether a larger size or aromaticity of the substituent might negatively impact PTE formation. The synthesis of precursor alkyne **4d** proved to be particularly challenging and required quite sophisticated adaptations. The intermediates involved in its synthesis are light-sensitive, thermo-labile, and simply nowhere near bench stability. The pursuit of the synthesis of **4d** commenced based on the hypothesis that reducing the diethoxy acetal-protected aldehyde **6**, an already well-known starting material for PTEs [16], should provide an easy access. A formal nucleophilic substitution was contemplated with a carbanion species such as Grignard arylmagnesium bromide **7** [24]. However, the reaction yielded a double-substituted species **8** with residual starting material (Figure 7). Variations in the reaction conditions, such as significantly low temperature (−60/−11/0 °C) and exploring the use of other carbanion sources, e.g., aryl lithium [25,26,27], exclusively led to compound **8**. While the nucleophilic attack in the *ortho*-position on the aza group was somewhat predictable based on the literature precedence, it was hoped to be observed only to a minor extent and not as the by-far major component. The observations associated with this process can be attributed to the stabilization of intermediate **9**, facilitated by coordination of the magnesium salt to the nitrogen atom.

It was briefly considered that **8** could be used for the synthetic procedure towards the pentathiepine. However, the inclusion of multiple aromatic rings would have inevitably led to a pronounced decrease of the solubility in aqueous media, which is already comparably poor in the typical PTEs derived from the molybdenum-mediated synthesis and would make utilization for subsequent biological testing entirely impossible [2,28,29].

**Figure 8:**
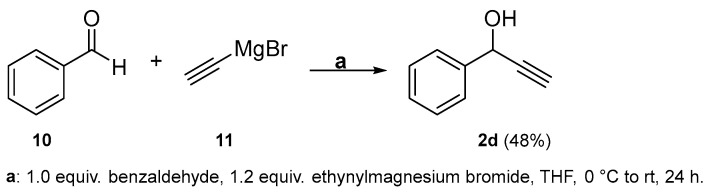
Synthesis of **2d**. N.B.: Lower temperatures lead to an increased amount of side products such as compound **12,** which was isolated from a reaction at −60 °C.

An alternative approach towards **4d** was pursued via synthesizing it essentially from scratch by adapting a procedure for related compounds from the literature [30]. First, benzaldehyde **10** was reduced using ethynylmagnesium bromide **11**. The reaction proceeded comparably efficiently at 0 °C in dry THF and yielded the desired alkyne **2d** (Figure 8).

**Figure 9:**
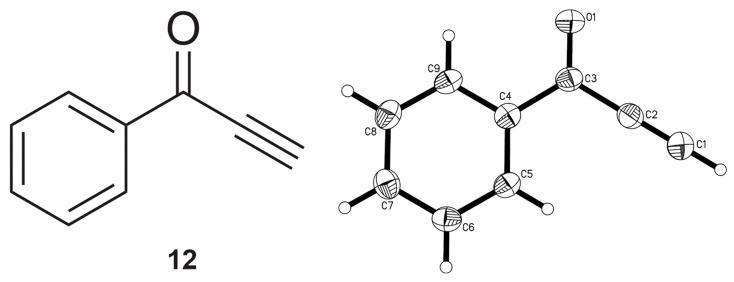
Chemical (**left**) and molecular (**right**) structures of 1-phenylprop-2-yn-1-one **12**, the side product from the Grignard reaction conducted at −60 °C. Ellipsoids are shown at the 50% probability level.

A synthesis at −60 °C in order to slow conversion down and have better control of the reaction unexpectedly resulted in the very opposite, i.e., a messier mixture of (side) products. It was possible to isolate one of the latter (known compound **12** [31,32,33]) in trace amounts from this attempt, which could then be characterized by SCXRD (Figure 9).

**Figure 10:**
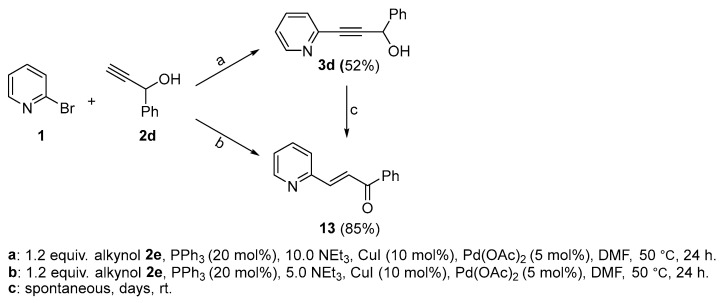
Sonogashira coupling reaction towards **3d** and complications arising from Lewis acid-supported or spontaneous rearrangements to **13**.

The subsequent classic Williamson-type etherification reaction [22] employing compound **2d** proved challenging. Even though the product could be detected by mass spectrometry, the reaction yielded a rather complex mixture of side products. Hence, a Sonogashira cross-coupling reaction with 2-bromopyridine **1** was attempted prior to etherification rather than later to eliminate the acetylenic proton and potentially render the follow-up intermediate compound more manageable. Standard conditions were employed, utilizing DMF as solvent with PdCl_2_, CuI, and PPh_3_. Notably, a high volume of the base NEt_3_ (10 equiv.) turned out to be a decisive factor, as the use of lower amounts (5 equiv. or less) resulted in the formation of a side product (**13**, Figure 10). Likely, **13** is the result of a domino coupling–isomerization reaction, as observed previously [34]. The reported respective rearrangement was supported by the presence of Pd(II), i.e., Lewis acid-catalyzed. In consequence, a significantly higher concentration of NEt_3_ facilitated the formation of **3d** in our case by serving as a counter Lewis and Brønsted base. Notably, **3d** required swift handling and protection from light, as it spontaneously and entirely converted itself over time to **13**.

**Figure 11:**
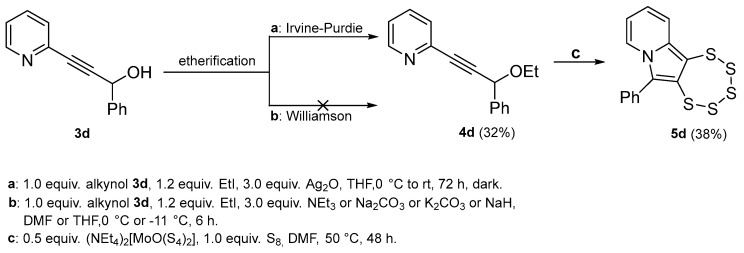
Top: Irvine–Purdie etherification of **3d** to yield **4d** followed by pentathiepine formation of **5d**. Bottom: Failed Williamson etherification.

The subsequently attempted transformation of **3d** to **4d** employing conventional Williamson etherification protocols did not yield the desired product. The reactions gave complex mixtures of impurities devoid of the target molecule. Variations including temperature adjustments from 0 °C down to −11 °C, replacing dry DMF solvent with dry THF, and employing distinct bases such as NEt_3_, Na_2_CO_3_, K_2_CO_3_, and NaH all failed. Notably, the use of alkoxide bases [35] offered a clean and reliable route for obtaining the related allene, as will be described below. Eventually Irvine–Purdie etherification was pursued as an alternative, which was initially developed for the methylation of sugars [36]. This method requires the use of Ag_2_O instead of more conventional bases [37] and the addition of activated zeolites to promote a more anhydrous environment. In our case, the reaction was run in darkness for 3 days at room temperature and indeed resulted in the successful formation of the desired product (Figure 11).

**Figure 12:**
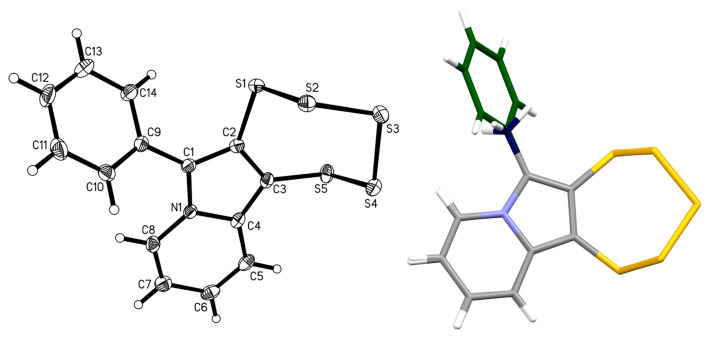
(**Left**): Molecular structure of **5d**. Ellipsoids are shown at the 50% probability level. (**Right**): Superimposition of the structures of **5c** (methyl carbon shown in dark blue) and **5d** (phenyl carbons shown in green). Overlay computed and visualized with Mercury [38].

Once the alkyne precursor **4d** was synthesized, thankfully, the subsequent PTE formation of **5d** under typical conditions progressed without any complications (Figure 11). The maximum in the UV–vis absorption spectrum of **5d** is even slightly more blue-shifted to 390.0 nm compared to **5c** (392.0 nm), although this difference is not significant. Both compounds deviate in their electronic structure from the previously investigated –OEt, bearing PTEs with a smaller HOMO–LUMO gap, which goes back to the presence of the oxygen bound to C-6 in the –OEt derivatives. The very close similarity of the spectra of **5c** and **5d** implies that the methyl and phenyl groups, which have different inductive/electronic effects as substituents, do not contribute (or at least do not contribute distinctly) to the molecules’ frontier orbitals. The molecular structure of **5d** was determined by SCXRD structural analysis (Figure 12). As usual, the metrical parameters observed are essentially in line with previous structural reports. Apparently, not even the substitution with a relatively large phenyl ring changes the preferred structural geometries of the pentathiepine. However, the two atom–atom distances within the 5-ring involving position C-6 (atom C1 in the structure) are slightly elongated for the phenyl derivative compared to the methyl derivative by roughly 0.01 Å, and for the bond to the substituent (C1–C9), the opposite is the case, with a respective 0.01 Å elongation for the methyl substituent relative to the phenyl substituent. Likely, in the case of the phenyl, the resonant π system of the backbone is extended so that it includes to a certain extent the aromatic substituent as well. However, the differences are rather subtle (see overlay of both structures, which shows hardly any variation of relative atom positions; Figure 12), and the electronic structure is likely affected only to a very minor extent, which is in accordance with the recorded very similar UV–vis absorption spectra of **5c** and **5d**.

### 2.4. Cyclic Acetal Precursors

Next, cyclic acetals were tested as starting materials for pentathiepine synthesis. This was carried out for three main reasons. (i) Naturally occurring pentathiepines possess an ethylamine side chain (–CH_2_CH_2_NH_2_) adjacent to the polysulfide ring. It was previously shown that the terminal –NH_2_ group, being both basic and nucleophilic, facilitates activating the bio-defensive mechanism by initiating an intramolecular nucleophilic attack on the polysulfur ring, resulting in its opening and the release of sulfur-based nucleophiles [39,40]. The pentathiepine product from cyclic acetal precursors was expected to bear a respective alcohol analogue of the amine functional group, albeit with a milder nucleophilicity, i.e., less prone to self-destruction as the amine would be [41,42] but at the same time a very good model thereof. (ii) The presence of a free alcohol function in the product should improve the pentathiepine’s solubility in water or aqueous solvent mixtures, a lack of which impairs biological testing and application. Poor aqueous solubility is one of the PTE characteristics that we often struggled with previously when assessing their biological activity [23]. And (iii) the cyclic acetal is an ideal precursor in order to investigate the fate of the abstracted OEt^−^ or, more generally, OR^−^ functional groups. The removal of the OEt^−^ ion is supposed to be the initiating mechanistic step in PTE formation, and the subsequent combination with the proton, which is eventually released as well, is expected to lead to the ethanol side product. With the cyclic acetal precursors, the dissociation of one of the two C–O bonds to the alkyne-bound carbon (later C-6 in the pentathiepine) will mimic the release of OEt^−^, while the fragment stays on the molecule during further transformations, by which the transfer of the proton to the alcoholate can also be evidenced with the chemical structure of the PTE product.

**Figure 13:**
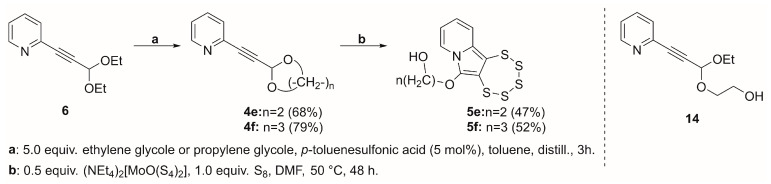
(**Left**): Synthesis of precursors **4e** and **4f** from **6** and transformation to the pentathiepines **5e** and **5f**, bearing free alcohol functions linked aliphatically to carbon C-6. (**Right**): Incompletely reacted side product **14** in the reaction towards **4e**.

The synthesis of the two precursor alkynes **4e** and **4f** was performed using classical aldehyde-protection protocols, which, in these specific cases, served as protecting-group-swapping reactions [43,44]. Both products were obtained by refluxing a toluene solution of precursor **6**, ethylene, or propylene glycol in the presence of catalytic amounts of trifluoromethanesulfonic acid (Figure 13). Toluene was then distilled off together with the formed ethanol. For the synthesis of the smaller cycle, it was also possible to isolate a partially substituted product, namely **14**.

**Figure 14:**
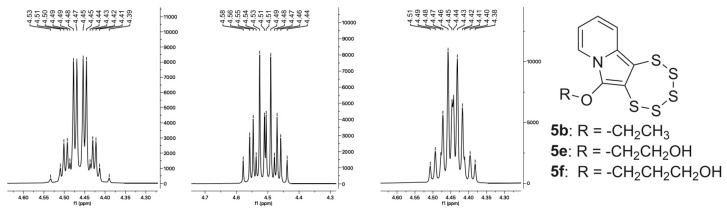
^1^H-NMR spectroscopic fingerprint sections for **5b** (**left**), **5e** (**center**), and **5f** (**right**), showing the diastereoisotopic –CH_2_– pattern, which confirms PTE formation for all known species bearing an –OCH_2_– moiety on the pyrrolic carbon C-6. (For full spectra, see previous publication for **5b** [16], Figure S31 for **5e**, and Figure S33 for **5f**.)

In subsequence, the two cyclic acetal precursors **4e** and **4f** were used for PTE synthesis under the typical conditions of the molybdenum-mediated pentathiepine formation. Both reactions succeeded, and the products were isolated and characterized. Their syntheses were unambiguously confirmed by the diastereoisotopic ^1^H-NMR patterns stemming from the protons of their –OCH_2_– moieties (Figure 14). The absorbance maxima in their UV–vis spectra lie exactly where those of previous species with the ethoxy substituents in position C-6 were found: 402.5 nm for **5e** and 403.5 nm for **5f**. This further confirms that the contribution of the ether oxygen atom to the HOMO is responsible for the red shift of the absorption peaks in the spectra of ether derivatives relative to those of **5c** and **5d**.

**Figure 15:**
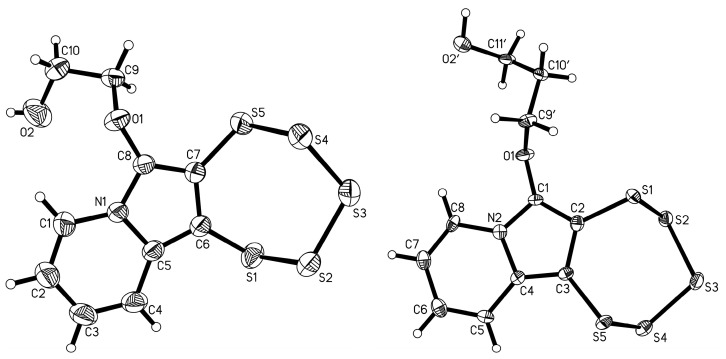
Molecular structures of **5e** (**left**) and **5f** (**right**) with free alcohol functions attached to carbon C-6 by an aliphatic linker. Ellipsoids are shown at the 50% probability level. The structure of **5f** is disordered in the –C_3_H_6_OH chain; only the major component (accounting for 52% of occupancy) is shown.

It was further possible to obtain single crystals of both products (**5e** and **5f**), which were then investigated by X-ray diffraction structural analysis (Figure 15). The alcohol hydrogen atoms in both cases were found in the electron density maps and refined nearly freely. In case of **5e**, the O–H distance had to be restrained, as in the crystal packing, two alcohol functions are located very close two each other, and the hydrogen atoms might be mobile to some extent in between the two adjacent oxygen atoms. In the case of **5f,** because of the disorder problem, the occupancies were constrained to that of the respective parent oxygen atom, while the locations were refined entirely freely.

With the confirmed chemical structures of **5e** and **5f**, it was possible to unambiguously confirm that the proton, which is released during the transformation from alkyne to pentathiepine, ends up on the alcoholate oxygen, which is generated in the initial, or at least a very early, step in the mechanism. It was also shown that it does not necessarily have to be an ethoxy that is part of the precursor, while the presence of at least one ether function is with a very high likelihood essential considering all observed or found to be impossible transitions investigated in this study.

### 2.5. Allene Precursor Variation

In the originally proposed reaction mechanism [15], the C≡C triple bond has two roles: (i) It will become oxidized; i.e., it is the reducing agent for the two sulfur atoms binding to the two carbon atoms and thereby becomes an inherent component of the pentathiepine unit (S–C=C–S); this is undisputedly proven by the chemical structure of the pentathiepine product. (ii) It is also part of the resonant π system of the molecule before, during, and after the transformation and helps in facilitating the formation of the *N*-heterocyclic 5-ring by allowing π-electron density to flow in between the *N*-heterocyclic 6-ring and the –CH_2_OEt moiety. With regard to the latter, an allene might be considered a putative intermediate resulting from a potential molecule rearrangement after OEt^−^ abstraction if such a rearrangement takes place prior to the attachment of the S_5_-moiety. In order to assess this hypothesis, an allene precursor (**4g**) was synthesized as a derivative of **4c**. Specifically in the context of indolizine derivative synthesis, a respective tautomerism in between alkyne and allene was proposed previously [45]. Various procedures for the synthesis of an allene ether from the corresponding alkyne tautomer have been reported in the literature. Methods involving the use of strong base or highly reactive BuLi [46] were tested but failed to produce the target compound. Eventually, the procedure described by Hoff et al. [47] was adapted, exposing **4c** to *t*-BuOK in dry THF, which efficiently yielded **4g**.

**Figure 16:**
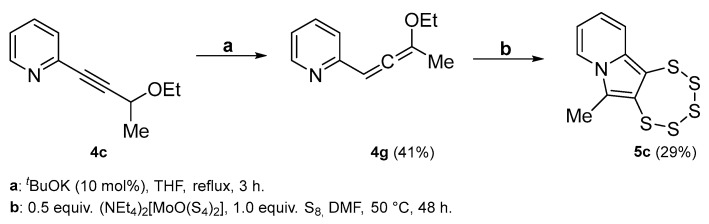
Synthesis of precursor **4g** from **4c** and transformation to the pentathiepine **5c** under standard conditions.

This allene derivative could indeed be transformed to **5c** when exposed to the same reaction conditions as **4c** (Figure 16), with even comparable yields. The successful reaction does not comprise unambiguous evidence for the allene intermediate in the proposed new reaction mechanism since the proton to be abstracted resides in distinct positions (here adjacent to the heterocycle/in the mechanism on the opposite end of the allene intermediate). Still, employing **4g** successfully at least strongly implies that the allene is indeed an active intermediate in the molybdenum-mediated pentathiepine formation.

## 3. Materials and Methods

### 3.1. Materials, Methods, and Instrumentation

#### 3.1.1. General Experimental Procedures

Most experiments were conducted under either nitrogen or argon atmosphere employing standard Schlenk techniques. Instances where reactions were exposed to air are explicitly indicated. Reagents and starting materials were utilized without additional purification. Tetrahydrofuran (THF) underwent pre-drying over KOH and CuCl, followed by further drying over Na prior to usage. Dimethylformamide (DMF) was dried by refluxing under argon for 48 h over P_2_O_5_. Compounds including Sonogashira products (**3a, 3c, 3d, 4′a,** and **6**), Williamson etherification (**4b** and **4c**), **and** pentathiepines (**5a, 5c, 5d, 5e,** and **5f**) were synthesized following the literature procedures [16,18,22,35]. Compound **4d** was synthesized with an adaptation of the Irvine–Purdie procedure [36,37]. Column chromatography purification was conducted using silica gel from VWR (particle size of 0.063–0.200 mm; 70–230 mesh ASTM). Flash chromatography purification was performed on an Interchim instrument 5.020 with columns PF-30SIHP-JP-F0080 and PF-30SIHP-JP-F0120 provided by the same company (Interchim, Montluçon, France). ^1^H- and ^13^C-NMR spectra were recorded on a Bruker Avance II 300 spectrometer (300 and 75.5 MHz, respectively, Bruker, Ettlingen, Germany) employing CD_2_Cl_2_, acetone-d_6_, and CD_3_CN dried over activated zeolites as solvents. Chemical shifts (δ) are reported in parts per million (ppm). Residual protons of the deuterated solvent were used as reference for ^1^H-NMR spectra, while the deuterated solvent itself was used as reference for ^13^C-NMR. Multiplicities are abbreviated as follows: s (singlet), d (doublet), t (triplet), q (quartet), m (multiplet), and *J* (coupling constant in Hertz). UV–vis spectra were recorded on a UV-3600 SHIMADZU UV–Vis-NIR spectrophotometer (Shimadzu, Tokyo, Japan). Mass spectra were obtained with an Advion Expression CMS with an APCI ionization source (Advion, Ithaca, NY, USA).

#### 3.1.2. Singe-Crystal X-ray Diffraction

The single-crystal X-ray diffraction (SCXRD) data of **3a**, **5d**–**f**, and **12** were recorded at 100 K or at room temperature on an XtaLAB Synergy diffractometer from Rigaku (Neu-Isenburg, Germany), with mirror-monochromated Cu-*K*α-radiation (λ = 1.54184 Å) and a hybrid pixel array detector (HyPix). Samples were mounted on LithoLoops produced by Molecular Dimensions (Sheffield, UK) and fixed on pins produced by Hampton Research (Aliso Viejo, CA, USA). The data for **5c** were collected at 170 K on a STOE-IPDS 2T diffractometer with graphite-monochromated Mo-*K*α-radiation (λ = 0.71073 Å). The sample was mounted on a glass fiber. Absorption corrections were performed using X-Red32 and X-Shape (by STOE & Cie GmbH 2010, Darmstadt, Germany) in the case of the Mo source or CrysAlisPro (different versions; Rigaku OD, 2022, Tokyo, Japan) in the case of the Cu source. All structures were solved by dual methods with SHELXT-2018 and refined by full-matrix least-squares techniques using the SHELXL-2018/19 executables and the WingX GUI [48,49,50]. All non-hydrogen atoms were refined with anisotropic displacement parameters. In the structure of **5c**, all hydrogen atoms were refined freely, as the data were of exceptionally good quality. All other C-bound hydrogen atoms in the other structures were refined isotropically at calculated positions using a riding model, with their *U*_iso_ values constrained to 1.2 times *U*_eq_ of their pivot atoms for aromatic or methylene hydrogen atoms and to 1.5 times *U*_eq_ for the methyl hydrogen atoms. All oxygen-bound hydrogen atoms were found and refined as freely as possible. In the case of **5e**, the O–H distance was fixed (DFIX), as the hydrogen tended to come too close to another adjacent alcohol function. In the case of **5f**, the displacement parameters were constrained to the parent oxygen atom (1.5 times *U*_eq_). The –C_3_H_6_OH chain of this molecule is also disordered over two orientations, which were modelled with SIMU and gave refined occupancies of 52% and 48%. General crystallographic, crystal, and refinement data are provided in the Supplementary Data Files. Crystallographic data were deposited with the Cambridge Crystallographic Data Centre, CCDC, 12 Union Road, Cambridge CB21EZ, UK. These data can be obtained free of charge upon providing the depository numbers CCDC 2363324 (**5d**), 2363325 (**5e**), 2363326 (**5f**), 23633247 (**5c**), 2363328 (**3a**), and 2363324 (**12**) by email (deposit@ccdc.cam.ac.uk) or their web interface (at http://www.ccdc.cam.ac.uk accessed on 8 August 2024).

### 3.2. Syntheses

#### 3.2.1. Sonogashira Cross-Coupling Reactions

Unless stated otherwise, Sonogashira coupling reactions were performed as follows: A mix of DMF (5.00 mL) and NEt_3_ (8.75 mL, 63.00 mmol, 10.00 equiv.) was introduced into an oven-dried Schlenk tube equipped with a side-arm stopcock and a stirring rod. The solution was degassed with nitrogen (N_2_) for 10 min and kept under N_2_ atmosphere throughout the preparation of the synthesis. Reactants were added in the following sequence: CuI (120 mg, 0.63 mmol, 0.10 equiv.) and then the proper alkyne (7.56 mmol, 1.20 equiv.), upon which the solution turned yellow. PPh_3_ (330 mg, 1.26 mmol, 0.20 equiv.) was added along with 2-bromopyridine (1.00 g, 6.30 mmol, 1.00 equiv.). Finally, Pd(OAc)_2_ (71 mg, 0.315 mmol, 0.05 equiv.) was introduced, causing the solution to rapidly change to a reddish-brown color. The mixture was stirred at 50 °C for 72 h. The reaction progress was monitored using TLC (eluent ratio 80/20 = Hex/EtOAc, *v*/*v*; 254 nm UV lamp; KMnO_4_ stain) and MS. Upon completion, the solvent was evaporated to dryness under vacuum using a cooling trap. The remaining residue was extracted with DCM and washed three times with saturated NH_4_Cl solution. The organic phase was recovered, dried over Na_2_SO_4_, and dried with a rotary evaporator. Lastly, column chromatography was carried out (eluent mix ratio Hex/EtOAc *v*/*v* specified for every product).

*3-(pyridin-2-yl)prop-2-yn-1-ol* (**3a**): Purified via flash chromatography (Hex/EtOAc = 50/50), reaction performed in THF at 40 °C, yield: 83%, white crystals, R_f_ = 0.2 (Hex/EtOAc = 50/50). ^1^H NMR (CD_2_Cl_2_, 300 MHz): δ 8.59–8.46 (m, 1H), 7.65 (td, 1H, *J* = 7.8, 1.8 Hz), 7.41 (dt, 1H, *J* = 7.8, 1.1 Hz), 7.23 (ddd, 1H, *J* = 7.5, 5.0, 1.3 Hz), 4.84 (t, 1H, *J* = 6.2 Hz), 4.52 (d, 2H, *J* = 6.1 Hz). ^13^C{^1^H} NMR (CD_2_Cl_2_, 75.5 MHz): δ 149.60, 142.70, 136.59, 127.25, 123.13, 89.01, 83.57, 50.60. MS (APCI) *m*/*z*: [M + H]^+^ calculated for C_8_H_8_NO 134.1; found 134.1.

*4-(pyridine-2-yl)but-3-yn-2-ol* (**3c**): Purified via flash chromatography (Hex/EtOAc = 60/40), yield: 78%, colorless oil, R_f_ = 0.3 (Hex/EtOAc = 50/50). ^1^H NMR (Acetone-d_6_, 300 MHz): δ 8.57 (dt, 1H, *J* = 5.0, 1.4 Hz), 7.77 (tt, 1H, *J* = 7.7, 1.8 Hz), 7.45 (dd, 1H, *J* = 7.8, 1.3 Hz), 7.34 (ddd, 1H, *J* = 7.8, 4.9, 1.3 Hz), 5.01 (d, 1H, *J* = 4.5 Hz), 4.76 (dt, 1H, *J* = 11.0, 5.2 Hz), 1.51 (d, 3H, *J* = 6.6 Hz). ^13^C{^1^H} NMR (Acetone-d_6_, 75.5 MHz): δ 151.76, 145.00, 138.32, 128.91, 124.93, 94.02, 84.20, 59.31, 25.81. MS (APCI) *m*/*z*: [M + H]^+^ calculated for C_9_H_10_NO 148.1; found 148.1.

*1-phenyl-3-(pyridin-2-yl)prop-2-yn-1-ol* (**3d**): Purified twice via flash chromatography (Hex/EtOAc = 70/30), yield: 52%, colorless oil, R_f_ = 0.3 (Hex/EtOAc = 85/15). ^1^H NMR (Acetone-d_6_, 300 MHz): δ 8.63–8.45 (m, 1H), 7.75 (td, 1H, *J* = 7.7, 1.8 Hz), 7.62 (dd, 1H, *J* = 7.1, 1.9 Hz), 7.50–7.22 (m, 5H), 5.72 (d, 1H, *J* = 4.5 Hz), 5.34 (d, 1H, *J* = 5.6 Hz). ^13^C{^1^H} NMR (Acetone-d_6_, 75 MHz): δ 151.48, 144.51, 143.21, 137.82, 129.81, 129.29, 128.55, 128.10, 124.65, 91.32, 86.20, 65.22, 65.12. MS (APCI) *m*/*z*: [M + H]^+^ calculated for C_14_H_12_NO 210.1; found 210.2.

*2-(pent-1-yn-1-yl)pyridine* (**4′a**): Purified via column chromatography (Hex/EtOAc = 90/10), yield: 81%, pale-yellow oil, R_f_ = 0.3 (Hex/EtOAc = 85/15). ^1^H NMR (CD_2_Cl_2_, 300 MHz): δ 8.47 (dt, 1H, *J* = 5.0, 1.3 Hz), 7.56 (td, 1H, *J* = 7.7, 1.9 Hz), 7.32 (dt, 1H, *J* = 7.8, 1.2 Hz), 7.13 (ddd, 1H, *J* = 7.7, 5.0, 1.2 Hz), 2.38 (t, 2H, *J* = 7.1 Hz), 1.61 (h, 2H, *J* = 7.2 Hz), 1.02 (t, 3H, *J* = 7.4 Hz). ^13^C{^1^H} NMR (CD_2_Cl_2_, 75.5 MHz): δ 150.14, 144.32, 136.23, 127.15, 122.58, 90.79, 81.02, 22.32, 21.50, 13.70. MS (APCI) *m*/*z*: [M + H]^+^ calculated for C_10_H_12_N 146.1; found 146.1.

*2-(3,3-diethoxyprop-1-yn-1-yl)pyridine* (**6**): Purified via flash chromatography (Hex/EtOAc = 90/10), yield: 58%, pale-brown oil, R_f_ = 0.2 (Hex/EtOAc = 80/20). ^1^H NMR (CD_2_Cl_2_, 300 MHz): δ 8.47 (ddd, 1H, *J* = 5.0, 1.8, and 1.0 Hz), 7,57 (td, 1H, *J* = 7.8 and 1.8 Hz), 7,37 (dt, 1H, *J* = 7.9 and 1.5 Hz), 7.17 (ddd, 1H, *J* = 7.61, 4.86, and 1.2 Hz), 5.38 (s, 1H), 3.75–3.50 (m, 4H), and 1.15 (t, 6H, *J* = 7.0 Hz). ^13^C{^1^H} NMR (CD_2_Cl_2_, 75.5 MHz): δ 150.5, 142.6, 136.4, 127.7, 123.7, 92.9, 84.4, 61.5, 15.2. MS (APCI) *m*/*z*: [M + H]^+^ calculated for C_12_H_16_NO_2_ 206.1; found 206.1.

*(E)-1-phenyl-3-(pyridin-2-yl)prop-2-en-1-one* (**13**): Purified via column chromatography (Hex/EtOAc = 70/30), yield: 85%, colorless oil, R_f_ = 0.5 (Hex/EtOAc = 85/15). ^1^H NMR (CD_3_CN, 300 MHz): δ 8.65 (dd, 1H, *J* = 4.8, 1.7 Hz), 8.14–7.98 (m, 3H), 7.84–7.48 (m, 7H), 7.39–7.30 (m, 1H). ^13^C{^1^H} NMR (CD_3_CN, 75.5 MHz): δ 190.15, 153.18, 150.18, 142.83, 137.87, 137.06, 133.14, 128.81, 128.51, 125.48, 124.98, 124.65. MS (APCI) *m*/*z*: [M + H]^+^ calculated for C_14_H_12_NO 210.1; found 210.2.

#### 3.2.2. Ethylation via Williamson Reaction

In a two-neck 100 mL round-bottom flask equipped with a side arm stopcock, flamed under vacuum, the proper alkynol (7.50 mmol, 1.00 equiv.) was added with anhydrous DMF (5.00 mL). This was followed by the addition of K_2_CO_3_ (3.11 g, 22.50 mmol, 3.00 equiv.), previously dried at 150 °C under vacuo for 8 h, and iodoethane (0.72 mL, 9 mmol, 1.20 equiv.). The reaction was stirred at rt and under N_2_ atmosphere throughout its preparation. The reaction was monitored via TLC (eluent ratio 80/20 = Hex/EtOAc, *v*/*v*; 254 nm UV lamp; KMnO_4_ stain). Upon completion, the solvent was evaporated to dryness under vacuum using a cooling trap. The remaining residue was extracted with DCM and washed three times with distilled water.

*2-(3-ethoxyprop-1-yn-1-yl)pyridine* (**4b**): Purification required chromatographic column (Hex/EtOAc = 70/30) followed by a distillation with a Kugelrohr apparatus (80 °C, 0.1 bar), yield: 47%, pale-yellow oil, R_f_ = 0.3 (Hex/EtOAc = 70/30). ^1^H NMR (CD_2_Cl_2_, 300 MHz): δ 8.53–8.40 (m, 1H), 7.57 (td, 1H, *J* = 7.7, 1.8 Hz), 7.35 (dt, 1H, *J* = 7.8, 1.1 Hz), 7.15 (ddd, 1H, *J* = 7.7, 4.9, 1.2 Hz), 3.55 (q, 2H, *J* = 7.0 Hz), 1.15 (t, 3H, *J* = 7.0 Hz). ^13^C{^1^H} NMR (CD_2_Cl_2_, 75.5 MHz): δ 149.98, 142.84, 136.06, 127.10, 122.99, 85.40, 85.13, 65.65, 58.20, 14.83. MS (APCI) *m*/*z*: [M + H]^+^ calculated for C_10_H_12_NO 162.1; found 162.1.

*2-(3-ethoxybut-1-yn-1-yl)pyridine* (**4c**): Purified via flash chromatography (Hex/EtOAc = 90/10), yield: 56%, colorless oil, R_f_ = 0.3 (Hex/EtOAc = 90/10). ^1^H NMR (CD_2_Cl_2_, 300 MHz): δ 8.44 (ddd, 1H, *J* = 4.9, 1.8, 1.0 Hz), 7.55 (td, 1H, *J* = 7.7, 1.8 Hz), 7.32 (dt, 1H, *J* = 7.8, 1.1 Hz), 7.13 (ddd, 1H, *J* = 7.7, 4.9, 1.2 Hz), 4.30 (q, 1H, *J* = 6.6 Hz), 3.73 (dq, 1H, *J* = 9.1, 7.0 Hz), 3.40 (dq, 1H, *J* = 9.1, 7.0 Hz), 1.40 (d, 3H, *J* = 6.7 Hz), 1.12 (t, 3H, *J* = 7.0 Hz). ^13^C{^1^H} NMR (CD_2_Cl_2_, 75.5 MHz): δ 150.29, 143.30, 136.37, 127.43, 123.22, 89.66, 84.36, 65.63, 64.67, 22.20, 15.37. MS (APCI) *m*/*z*: [M + H]^+^ calculated for C_11_H_14_NO 176.1; found 176.1.

#### 3.2.3. Ethylation via Irvine–Purdie Reaction

In a two-neck 100 mL round-bottom flask equipped with a side arm stopcock, flamed under vacuo, alkynol **3d** (0.50 g, 2.392 mmol, 1.00 equiv.) was added with anhydrous DMF (10.00 mL), followed by the addition of activated zeolites, iodoethane (0.23 mL, 2.870 mmol, 1.20 equiv.), and lastly Ag_2_O (1.69 g, 7.176 mmol, 3.00 equiv.), previously dried at 150 °C under vacuo for 8 h. The reaction was stirred at rt under N_2_ atmosphere and protected from light throughout its preparation. The reaction was monitored via TLC (eluent ratio 80/20 = Hex/EtOAc, *v*/*v*; 254 nm UV lamp; KMnO_4_ stain). Upon completion, the solvent was evaporated to dryness under vacuum using a cooling trap. The remaining residue was extracted with DCM and washed three times with distilled water. The organic phase was recovered, dried over Na_2_SO_4_, and dried with a rotary evaporator. Lastly, the product was purified via flash chromatography (eluent Hex/EtOAc = 85/15, *v*/*v*).

*2-(3-ethoxy-3-phenylprop-1-yn-1-yl)pyridine* (**4d**): yield: 32%, R_f_ = 0.2 (Hex/EtOAc = 80/20). ^1^H NMR (CD_2_Cl_2_, 300 MHz): δ 9.13–8.98 (m, 1H), 8.21–8.06 (m, 3H), 8.00–7.86 (m, 4H), 7.76 (ddd, 1H, *J* = 7.7, 4.8, 1.3 Hz), 7.00 (s, 1H), 4.71 (qd, 2H, *J* = 7.1, 2.2 Hz), 1.79 (t, 3H, *J* = 7.1 Hz). ^13^C{^1^H} NMR (CD_2_Cl_2_, 75 MHz): δ 154.52, 150.49, 142.53, 136.63, 136.53, 129.68, 129.15, 128.03, 127.77, 123.87, 87.26, 84.84, 69.72, 65.02, 14.38. MS (APCI) *m*/*z*: [M + H]^+^ calculated for C_16_H_16_NO 238.1; found 238.2.

#### 3.2.4. Aldehyde Reduction

In a two-neck 200 mL round-bottom flask equipped with a side arm stopcock and a dropping funnel, flamed under vacuum, benzaldehyde **10** (5.00 g, 4.81 mL, 47.081 mmol, 1.00 equiv.) was added and cooled to 0 °C with an ice bath. A 0.5 M molar solution of ethynylmagnesium bromide **11** (94.16 mL) was added to the dropping funnel. The solution was added to the aldehyde in 2 h while stirring. The final mix was left to warm up to room temperature and stirred for another 12 h. The reaction was monitored via TLC (eluent ratio 80/20 = Hex/EtOAc, *v*/*v*; 254 nm UV lamp; KMnO_4_ stain). Upon completion, the final mixture was quenched and then washed three times with NH_4_Cl saturated solution. The organic phase was recovered, dried over Na_2_SO_4_, and dried with a rotary evaporator. Lastly, the product was purified via flash chromatography (Hex/EtOAc = 85/15, *v*/*v*).

*1-phenylprop-2-yn-1-ol* (**2d**): yield: 48%, pale-yellow oil, R_f_ = 0.2 (Hex/EtOAc = 80/20). ^1^H NMR (Acetone-d_6_, 300 MHz): δ 7.67–7.49 (m, 2H), 7.45–7.23 (m, 3H), 5.49 (dd, 1H, *J* = 5.9, 2.3 Hz), 5.05 (dd, 1H, *J* = 6.0, 1.5 Hz), 3.06 (d, 1H, *J* = 2.4 Hz). ^13^C{^1^H} NMR (Acetone, 75 MHz): δ 143.18, 129.66, 129.15, 127.91, 86.27, 75.60, 64.73. MS (APCI) *m*/*z*: [M + H]^+^ calculated for C_9_H_9_O 133.1; found 133.2.

#### 3.2.5. Nucleophilic Attack on Diethyl Acetal

To a three-neck 250 mL round-bottom flask equipped with a side arm stopcock and a reflux condenser, magnesium turnings (0.28 g, 11.690 mmol, 1.20 equiv.) were added and flamed under vacuum with a heating gun. After cooling to room temperature, three cycles of nitrogen–vacuum were performed, and 100 mL of anhydrous THF was added. Subsequently, phenyl bromide (2.00 g, 10.716 mmol, 1.1 equiv.) and a catalytic amount of iodine I_2_ (0.12 g, 0.487 mmol, 0.05 equiv.) were added. The reddish color of the dissolved iodine immediately disappeared to give a limpid, colorless solution. The reaction was stirred at room temperature until the exothermicity of the reaction decreased. (No more boiling of the solvent was observed.) The mixture was then refluxed until the magnesium was almost consumed and let to cool down to room temperature. Under nitrogen atmosphere, the solution was transferred with a cannula to a dropping funnel connected to a two-neck 250 mL round-bottom flask previously flamed under vacuum and containing **7** (2.00 g, 9.742 mmol, 1.00 equiv.) dissolved in 50 mL of anhydrous THF. The Grignard solution was slowly added at room temperature, and the reaction was further stirred for 12 h. Upon completion, the final mixture was quenched and then washed three times with NH_4_Cl saturated solution. The organic phase was recovered, dried over Na_2_SO_4_, and dried with a rotary evaporator. Lastly, the product was purified via column chromatography (Hex/EtOAc = 80/20, *v*/*v*).

*2-(3-ethoxy-3-phenylprop-1-yn-1-yl)-6-phenylpyridine* (**8**): yield: 39%, pale-yellow oil, R_f_ = 0.3 (Hex/EtOAc = 80/20). ^1^H NMR (CD_2_Cl_2_, 300 MHz): δ 8.41 (d, 1H, *J* = 4.9 Hz), 7.43 (td, 1H, *J* = 7.7, 1.9 Hz), 7.28–6.91 (m, 12H), 6.37 (s, 1H), 5.57 (s, 1H), 3.76–3.48 (m, 2H), 0.93 (t, 3H, *J* = 7.1 Hz). ^13^C{^1^H} NMR (CD_2_Cl_2_, 75.5 MHz): δ 163.58, 148.99, 146.49, 142.12, 140.61, 135.97, 129.79, 128.44, 128.32, 127.77, 126.46, 126.33, 124.74, 124.14, 121.24, 121.19, 68.57, 54.52, 54.48, 54.16, 53.80, 53.44, 53.08, 32.03, 15.29. MS (APCI) *m*/*z*: [M + H]^+^ calculated for C_22_H_20_NO 314.2; found 314.3.

#### 3.2.6. Diethyl Acetal Exchange Reaction

Compound **7** (2.00 g, 9.742 mmol, 1.00 equiv.), *p*-toluenesulfonic acid (0.08 g, 0.487 mmol, 0.05 equiv.), and the proper glycole (48.71 mmol, 5.00 equiv.) were dissolved with toluene (1.00 L) in a two-neck 2 L round-bottom flask equipped with a stirring rod and a Claisen distillation apparatus. Toluene was distilled out until the complete disappearance of the starting material. The reaction was monitored via TLC (eluent Et_2_O; 254 nm UV lamp). When the starting material was not completely converted, more toluene was added to the reaction, and the distillation continued until completion. The residue was quenched and washed three times with NaHCO_3_-saturated solution. The organic phase was recovered, dried over Na_2_SO_4_, and dried with a rotary evaporator. Lastly, the product was purified with column chromatography (Et_2_O).

*2-((1,3-dioxolan-2-yl)ethynyl)pyridine* (**4e**): yield: 68%, pale-yellow oil, R_f_ = 0.7 (Et_2_O). ^1^H NMR (CD_2_Cl_2_, 300 MHz): δ 8.54 (ddd, 1H, *J* = 4.9, 1.8, 1.0 Hz), 7.65 (td, 1H, *J* = 7.7, 1.8 Hz), 7.43 (dt, 1H, *J* = 7.8, 1.1 Hz), 7.25 (ddd, 1H, *J* = 7.7, 4.9, 1.2 Hz), 5.86 (s, 1H), 4.16–3.87 (m, 4H). ^13^C{^1^H} NMR (CD_2_Cl_2_, 75.5 MHz): δ 150.39, 142.24, 136.44, 127.70, 123.82, 93.38, 84.37, 84.17, 64.91. MS (APCI) *m*/*z*: [M + H]^+^ calculated for C_10_H_10_NO_2_ 176.1; found 176.1.

*2-((1,3-dioxan-2-yl)ethynyl)pyridine* (**4f**): yield: 79%, colorless oil, R_f_ = 0.6 (Et_2_O). ^1^H NMR (Acetone-d_6_, 300 MHz): δ 8.47 (ddd, 1H, *J* = 4.9, 1.8, 1.0 Hz), 7.58 (td, 1H, *J* = 7.7, 1.8 Hz), 7.38 (dt, 1H, *J* = 7.8, 1.1 Hz), 7.18 (ddd, 1H, *J* = 7.7, 4.9, 1.2 Hz), 5.52 (s, 1H), 4.27–4.07 (m, 2H), 3.85–3.66 (m, 2H), 1.90–1.48 (m, 2H). ^13^C{^1^H} NMR (Acetone-d_6_, 75 MHz): δ 150.38, 142.22, 136.43, 127.70, 123.83, 90.76, 84.86, 83.49, 65.19, 25.94, 25.70. MS (APCI) *m*/*z*: [M + H]^+^ calculated for C_11_H_12_NO_2_ 190.1; found 190.1.

*2-((1-ethoxy-3-(pyridin-2-yl)prop-2-yn-1-yl)oxy)ethan-1-ol* (**14**): yield: 15%, pale-yellow oil, R_f_ = 0.2 (Et_2_O). ^1^H NMR (CD_2_Cl_2_, 300 MHz): δ 8.48 (ddd, *J* = 4.9, 1.8, 0.9 Hz, 1H), 7.59 (td, *J* = 7.7, 1.8 Hz, 1H), 7.38 (dt, *J* = 7.8, 1.1 Hz, 1H), 7.18 (ddd, *J* = 7.7, 4.9, 1.2 Hz, 1H), 5.45 (s, 1H), 3.87–3.45 (m, 6H), 2.95 (s, 1H), 1.15 (t, *J* = 7.1 Hz, 3H). ^13^C{^1^H} NMR (CD_2_Cl_2_, 75.5 MHz): δ 150.39, 142.25, 136.60, 127.75, 123.89, 92.28, 84.75, 84.03, 67.64, 61.90, 30.40, 15.14. MS (APCI) *m*/*z*: [M + H]^+^ calculated for C_12_H_16_NO_3_ 222.1; found 222.2.

#### 3.2.7. Tautomerization from Alkyne to Allene

In a three-neck 100 mL round-bottom flask equipped with a side-arm stopcock, a stirring rod, and a reflux condenser and previously flamed under vacuum, **4c** (1.50 g, 8.567 mmol, 1.00 equiv.) was dissolved in 50 mL of anhydrous THF under N_2_ atmosphere. A catalytic amount of *^t^*BuOK (0.10 g, 0.857 mmol, 0.10 equiv.) was added, and the solution was refluxed for 2 h. The reaction was monitored via TLC (eluent ratio 80/20 = Hex/EtOAc, *v*/*v*; 254 nm UV lamp; KMnO_4_ stain). Upon completion, the reaction was quenched and washed three times with NaHCO_3_-saturated solution. The organic phase was recovered, dried over Na_2_SO_4_, and dried with a rotary evaporator. Lastly, the product was purified via column chromatography (eluent Hex/EtOAc = 90/10, *v*/*v*).

*2-(3-ethoxybuta-1,2-dien-1-yl)pyridine* (**4g**): yield 41%, colorless oil, R_f_ = 0.7 (Hex/EtOAc = 80/20). ^1^H NMR (300 MHz, CD_2_Cl_2_): δ 8.53 (dt, 1H, *J* = 4.8, 1.5 Hz), 7.62 (td, 1H, *J* = 7.7, 1.9 Hz), 7.28 (dd, 1H, *J* = 7.9, 1.2 Hz), 7.17–6.89 (m, 3H), 4.46–4.25 (m, 2H), 3.88 (q, 2H, *J* = 7.0 Hz), 1.39 (t, 3H, *J* = 7.0 Hz). ^13^C{^1^H} NMR (CD_2_Cl_2_, 75 MHz): δ 158.53, 155.51, 149.94, 136.68, 129.19, 128.17, 122.84, 122.42, 89.76, 63.40, 14.64. MS (APCI) *m*/*z*: [M + H]^+^ calculated for C_11_H_14_NO 176.1; found 176.1.

#### 3.2.8. Synthetic Procedures for Pentathiepine Formation

Pentathiepine syntheses were executed following the method described by Zubair et al. [15]. The appropriate quantities of alkynes (1.00 mmol, 1.00 equiv.), molybdenum–oxo-bistetrasulfido complex (0.50 mmol, 0.50 equiv.), and elemental sulfur (1.00 mmol, 1.00 equiv.) were introduced into flame-sealed Schlenk tubes containing a magnetic stirring bar and purged of air under vacuum. Then, the system underwent three cycles of nitrogen–vacuum purging, after which 3 mL of dry DMF was added. The resulting heterogeneous solution was stirred at 50 °C for 48 h. The reaction was stopped, and the solvent was removed under vacuum using a cooling trap. The product was purified using column chromatography commencing with 100% hexane and, when necessary, transitioning in gradient to an increased polarity of the eluent up to a mix of hexane and EtOAc, the ratio of which (*v*/*v*) is specified for every molecule below. This ensured the removal of sulfur. Its presence (Rf = 0.95) was checked via TLC with a 254 nm UV lamp.

*6-methyl-[1,2,3,4,5]pentathiepino[6,7-a]indolizine* (**5c**): yield: 32%, orange crystalline solid, R_f_ = 0.2 (hexane). ^1^H NMR (CD_2_Cl_2_, 300 MHz): δ 7.67 (d, *J* = 7.1 Hz, 1H), 7.57 (dt, *J* = 9.1, 1.2 Hz, 1H), 7.04–6.92 (m, 1H), 6.74 (td, *J* = 6.8, 1.3 Hz, 1H), 2.57 (s, 3H). ^13^C{^1^H} NMR (CD_2_Cl_2_, 75 MHz): δ 136.21, 128.83, 126.66, 123.54, 121.83, 118.68, 113.19, 111.42, 10.64. MS (APCI) *m*/*z*: [M + H]^+^ calculated for C_9_H_8_NS_5_ 289.9; found 290.0.

*6-phenyl-[1,2,3,4,5]pentathiepino[6,7-a]indolizine* (**5d**): yield: 38%, orange crystalline solid, R_f_ = 0.2 (hexane). ^1^H NMR (CD_2_Cl_2_, 300 MHz): δ 7.84 (d, 1H, *J* = 8.1 Hz), 7.63–7.28 (m, 6H), 6.95 (dd, 1H, *J* = 9.1, 6.6 Hz), 6.57 (t, 1H, *J* = 6.9 Hz). ^13^C{^1^H} NMR (CD_2_Cl_2_, 75 MHz): δ 136.59, 131.36, 129.55, 129.27, 129.14, 124.05, 122.95, 118.90, 113.49. MS (APCI) *m*/*z*: [M + H]^+^ calculated for C_14_H_10_NS_5_ 351.9; found 352.1.

*2-([1,2,3,4,5]pentathiepino[6,7-a]indolizin-6-yloxy)ethan-1-ol* (**5e**): flash chromatography in gradient up to Hex/EtOAc = 60/40. Yield: 47%, orange crystalline solid, R_f_ = 0.2 (Hex/EtOAc = 60/40). ^1^H NMR (CD_2_Cl_2_, 300 MHz): δ 7.86 (dd, 1H, *J* = 7.1, 1.2 Hz), 7.50 (dd, 1H, *J* = 9.1, 1.3 Hz), 6.90 (ddd, *J* = 9.2, 6.6, 1.1 Hz, 1H), 6.66 (td, 1H, *J* = 6.8, 1.2 Hz), 4.56–4.35 (m, 2H), 3.95 (q, 2H, *J* = 4.7 Hz), 2.07 (t, 1H, *J* = 5.8 Hz). ^13^C{^1^H} NMR (CD_2_Cl_2_, 75 MHz): δ 141.99, 132.78, 123.37, 123.07, 119.95, 115.90, 114.33, 110.74, 79.50, 63.30.MS (APCI) *m*/*z*: [M + H]^+^ calculated for C_10_H_10_NO_2_S_5_ 335.9; found 336.0.

*3-([1,2,3,4,5]pentathiepino[6,7-a]indolizin-6-yloxy)propan-1-ol* (**5f**): flash chromatography in gradient up to Hex/EtOAc = 50/50. Yield: 52%, orange crystalline solid, R_f_ = 0.2 (Hex/EtOAc = 60/40). ^1^H NMR (CD_2_Cl_2_, 300 MHz): δ 7.77 (dt, 1H, *J* = 7.1, 1.1 Hz), 7.47 (dt, 1H, *J* = 9.2, 1.2 Hz), 6.87 (ddd, 1H, *J* = 9.2, 6.6, 1.1 Hz), 6.63 (ddd, 1H, *J* = 7.1, 6.6, 1.2 Hz), 4.51 (qt, 2H, *J* = 9.6, 6.1 Hz), 4.00–3.81 (m, 2H), 2.07 (p, 2H, *J* = 6.1 Hz), 1.76–1.63 (m, 1H). ^13^C{^1^H} NMR (CD_2_Cl_2_, 75 MHz): δ 140.53, 130.81, 121.35, 121.06, 118.09, 113.28, 112.41, 108.89, 73.58, 59.20, 54.18, 53.82, 53.46, 53.10, 52.74, 32.70. MS (APCI) *m*/*z*: [M + H]^+^ calculated for C_11_H_12_NO_2_S_5_ 349.9; found 350.1.

## 4. Conclusions

In the course of trying to validate the reaction mechanism proposed for pentathiepine formation, as early as 2013, significant changes were made to the employed precursors with regard to the otherwise omnipresent two –OEt functional groups in the alkyne starting materials, resulting in one of these being always present in the products in the past. This study confirms that a single –OEt or, more generally, one ether function is sufficient to facilitate pentathiepine formation; yet, without it, the reaction does not proceed. It was further confirmed that in the case that two ether functions are present, only one is disconnected, while the second remains bound to the molecule. The proton, which is also released during the reaction, inevitably ends up on the oxygen that was initially discharged to jointly form the neutral alcohol. This study grants access to a functionalization of the C-6 atom of the pyrrolic ring, which is the only position directly adjacent to the PTE moiety that can be addressed. The successful application of an allene for PTE synthesis confirms that the delocalization of π-electron density is likely crucial for the reaction and that an allene may be an active intermediate in the reaction sequence. Four new pentathiepines were synthesized in course of this study, which comprise the very first at C-6 non-OEt functionalized derivatives from the molybdenum-mediated reaction. By this, it is possible to install functional groups that can improve, e.g., solubility in water or aqueous solvent mixtures (glycol ethers). It further facilitates investigating the effect that electron withdrawing or pushing substituents on this position may have on the electronic structures; from the first spectroscopic and metrical data of this study, the respective effect does not appear to be strong. However, this work still provides a starting point to modulating the resultant pentathiepines’ (physico)-chemical characteristics, which has not been attempted or exploited before and thereby comprises an unprecedented synthetic strategy.

## Figures and Tables

**Table 1:** Summary of the starting materials tested for the C-6 functionalization of indolizine-derived PTEs.

Starting Material		Product		Yield (%)
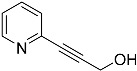	**3a**	None		/
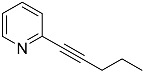	**4′a**	None		/
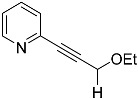	**4b**	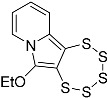	**5b**	Traces ^1^
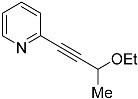	**4c**	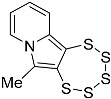	**5c**	32 ^2^
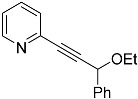	**4d**	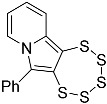	**5d**	38
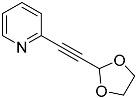	**4e**	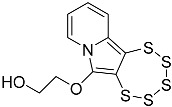	**5e**	47
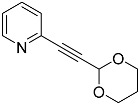	**4f**	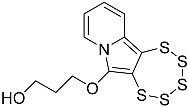	**5f**	52
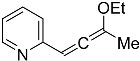	**4g**	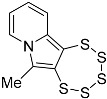	**5c**	29 ^2^

^1^ Confirmed crystallographically. ^2^ Yields are comparable employing **4c** and **4g** as precursor.

## Data Availability

The crystallographic data were deposited with the CCDC and are available by download via the deposition numbers (see experimental Section 3.1.2). Spectra, if not part of the manuscript, are provided in the Supplementary Materials. Original data are available from the authors upon request.

## Supplementary Materials

The following supporting information can be downloaded at: https://www.mdpi.com/article/10.3390/molecules29163806/s1, Figures S1–S38: ^1^H, ^13^C NMR spectra of **3a**, **3c**, **4’a**, **3d**, **6**, **4b**, **4c**, **4d**, **2d**, **8**, **4e**, **4f**, **4g**, **5c**, **5d**, **5e**, **5f**, **13**, **14**. Figures S39–S42: UV-vis spectra of **5c**, **5d**, **5e**, **5f**. Figures S43–S60: APCI Mass spectra of **3a**, **3c**, **4c**, **4g**, **4b**, **4’a**, **2d**, **3d**, **4d**, **13**, **14**, **8**, **4e**, **4f**, **5c**, **5d**, **5e**, **5f**. Figure S61: Tautomerism of precursor **4g** (2-(3-ethoxybuta-1,2-dien-1-yl)pyridine to the left and 2-(3-ethoxybuta-1,3-dien-1-yl)pyridine to the right). Tables S1–S6: Crystal data and structure refinement for **3a**, **12**, **5c**, **5d**, **5e**, **5f**.
