# Correction: Tallarita et al. The Role of –OEt Substituents in Molybdenum-Assisted Pentathiepine Formation—Access to Diversely Functionalized Azines. *Molecules* 2024, *29*, 3806

**DOI:** 10.3390/molecules30214189

**Published:** 2025-10-27

**Authors:** Roberto Tallarita, Lukas M. Jacobsen, Siva S. M. Bandaru, Benedict J. Elvers, Carola Schulzke

**Affiliations:** 1 Institute of Biochemistry, Bioinorganic Chemistry, University of Greifswald, Felix-Hausdorff-Str. 4, 17489 Greifswald, Germany; roberto.tallarita@uni-greifswald.de (R.T.); lukasmanuel.jacobsen@stud.uni-greifswald.de (L.M.J.); siva.bandaru@uni-greifswald.de (S.S.M.B.); benedic@umich.edu (B.J.E.); 2 Department of Chemistry, University of Michigan, Ann Arbor, MI 48109-1055, USA

## Supplemental Materials

In the original publication [1], the Supplementary Materials file was submitted but could not be accessed for technical reasons. As part of this correction, the authors are including Figure S61, which demonstrates that precursor **4g** (data from the original publication) is a tautomer.

The Corrected Supplementary Materials should be read as follows:

**Supplementary Materials:** The following supporting information can be downloaded at: https://www.mdpi.com/article/10.3390/molecules29163806/s1, Figures S1–S38: ^1^H, ^13^C NMR spectra of **3a**, **3c**, **4’a**, **3d**, **6**, **4b**, **4c**, **4d**, **2d**, **8**, **4e**, **4f**, **4g**, **5c**, **5d**, **5e**, **5f**, **13**, **14**. Figures S39–S42: UV-vis spectra of **5c**, **5d**, **5e**, **5f**. Figures S43–S60: APCI Mass spectra of **3a**, **3c**, **4c**, **4g**, **4b**, **4’a**, **2d**, **3d**, **4d**, **13**, **14**, **8**, **4e**, **4f**, **5c**, **5d**, **5e**, **5f**. Figure S61: Tautomerism of precursor **4g** (2-(3-ethoxybuta-1,2-dien-1-yl)pyridine to the left and 2-(3-ethoxybuta-1,3-dien-1-yl)pyridine to the right). Tables S1–S6: Crystal data and structure refinement for **3a**, **12**, **5c**, **5d**, **5e**, **5f**.

The authors state that the scientific conclusions are unaffected by the issue. This correction was approved by the Academic Editor. The original publication has also been updated.
