# Supplementary material for: The Role of –OEt Substituents in Molybdenum-Assisted Pentathiepine Formation—Access to Diversely Functionalized Azines
Source: Molecules. 2024 Aug 11;29(16):3806. doi: 10.3390/molecules29163806 (PMC12696505; doi:10.3390/molecules29163806)
Supplement: Supplementary file 1 [file molecules-29-03806-s001.zip › molecules-3105066-supplementary.pdf]

Supporting Information

The Role of –OEt Substituents in Molybdenum-Assisted Pentathiepine Formation—Access to Diversely Functionalized Azines.

Roberto Tallarita [1], Lukas M. Jacobsen [1], Siva S. M. Bandaru [1], Benedict J. Elvers [1,2] and Carola Schulzke [1,*]

[1] Institute of Biochemistry, Bioinorganic Chemistry, University of Greifswald, Felix-Hausdorff-Str. 4, 17489 Greifswald, Germany; roberto.tallarita@uni-greifswald.de (R.T.); lukasmanuel.jacobsen@stud.uni-greifswald.de (L.M.J.); siva.bandaru@uni-greifswald.de (S.S.M.B.); benedic@umich.edu (B.J.E.)

[2] Department of Chemistry, University of Michigan, Ann Arbor, MI 48109-1055, USA

* Correspondence: carola.schulzke@uni-greifswald.de; Tel.: +49-3034-420-4321

Table of content

Figures S1 – S38: ^{1}H, ^{13}C NMR spectra of **3a, 3c, 4'a, 3d, 6, 4b, 4c, 4d, 2d, 8, 4e, 4f, 4g, 5c, 5d, 5e, 5f, 13, 14.**
Figures S39 – S42: UV-vis spectra of **5c**, **5d**, **5e**, **5f**.
Figures S43 – S60: APCI Mass spectra of **3a**, **3c**, **4c**, **4g**, **4b**, **4'a**, **2d**, **3d**, **4d**, **13**, **14**, **8**, **4e**, **4f**, **5c**, **5d**, **5e**, **5f**.
Figure S61: Tautomerism of precursor 4g (2-(3-ethoxybuta-1,2-dien-1-yl)pyridine to the left and 2-(3-ethoxybuta-1,3-dien-1-yl)pyridine to the right).
Tables S1 – S6: Crystal data and structure refinement for **3a**, **12**, **5c**, **5d**, **5e**, **5f**.

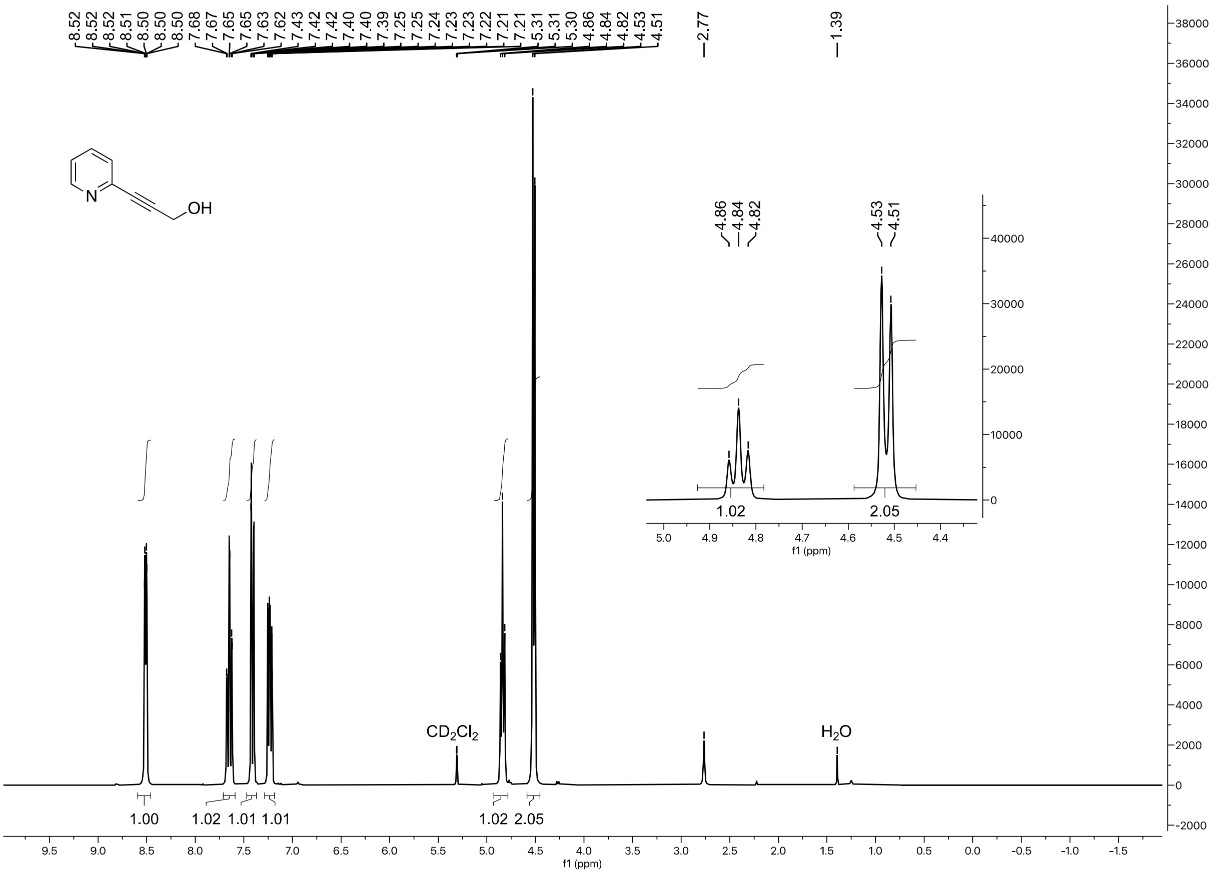

Figure S1. ^{1}H NMR spectrum of 3-(pyridin-2-yl)prop-2-yn-1-ol (**3a**).

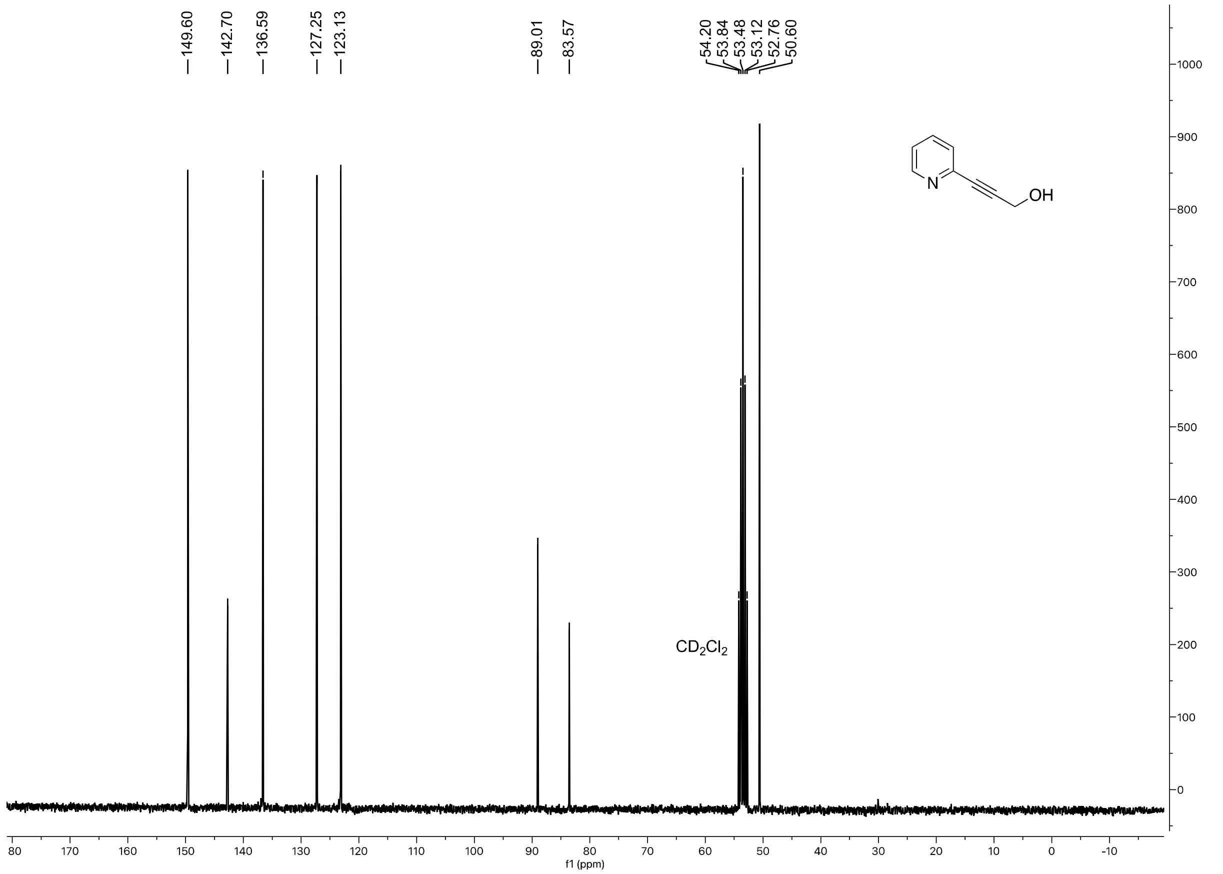

Figure S2. ^{13}C NMR spectrum of 3-(pyridin-2-yl)prop-2-yn-1-ol (**3a**).

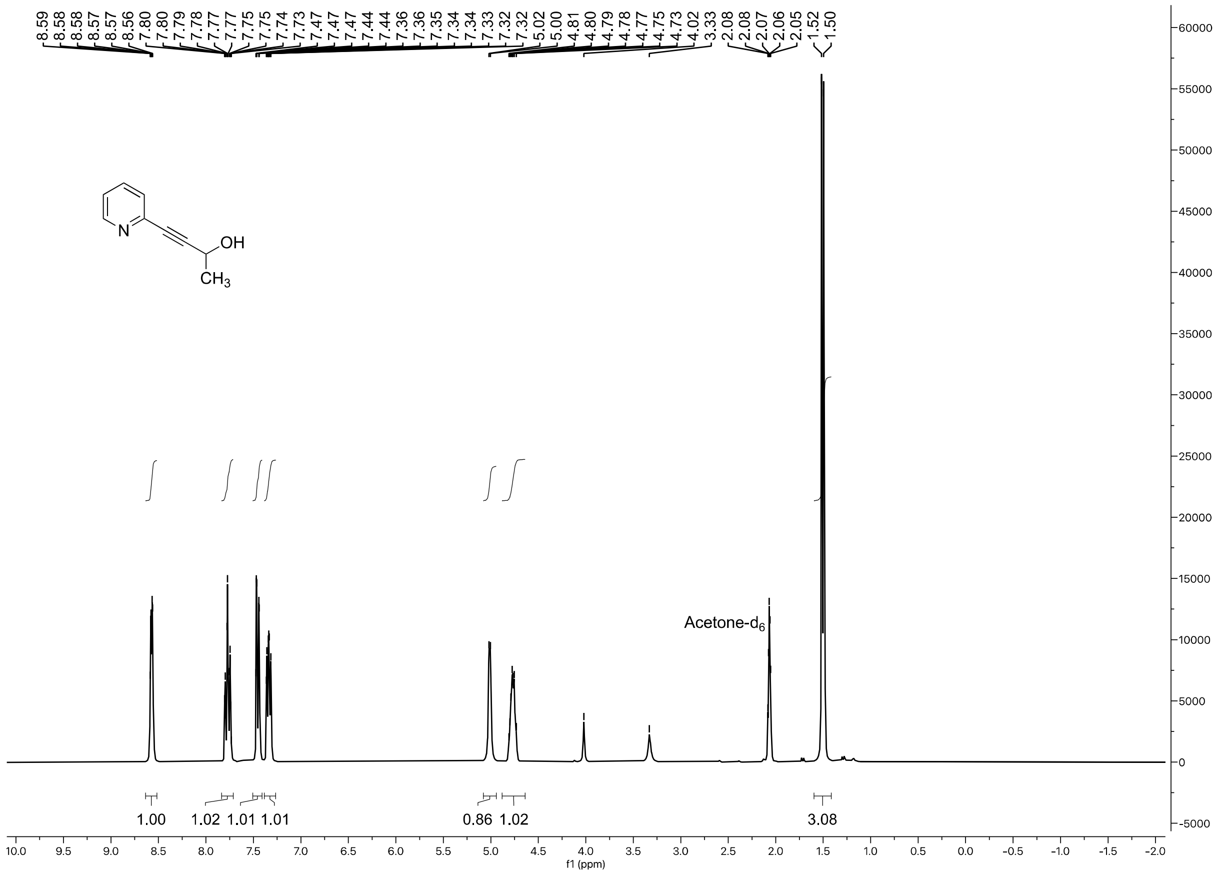

Figure S3. ^{1}H NMR spectrum of 4-(pyridine-2-yl)but-3-yn-2-ol (**3c**).

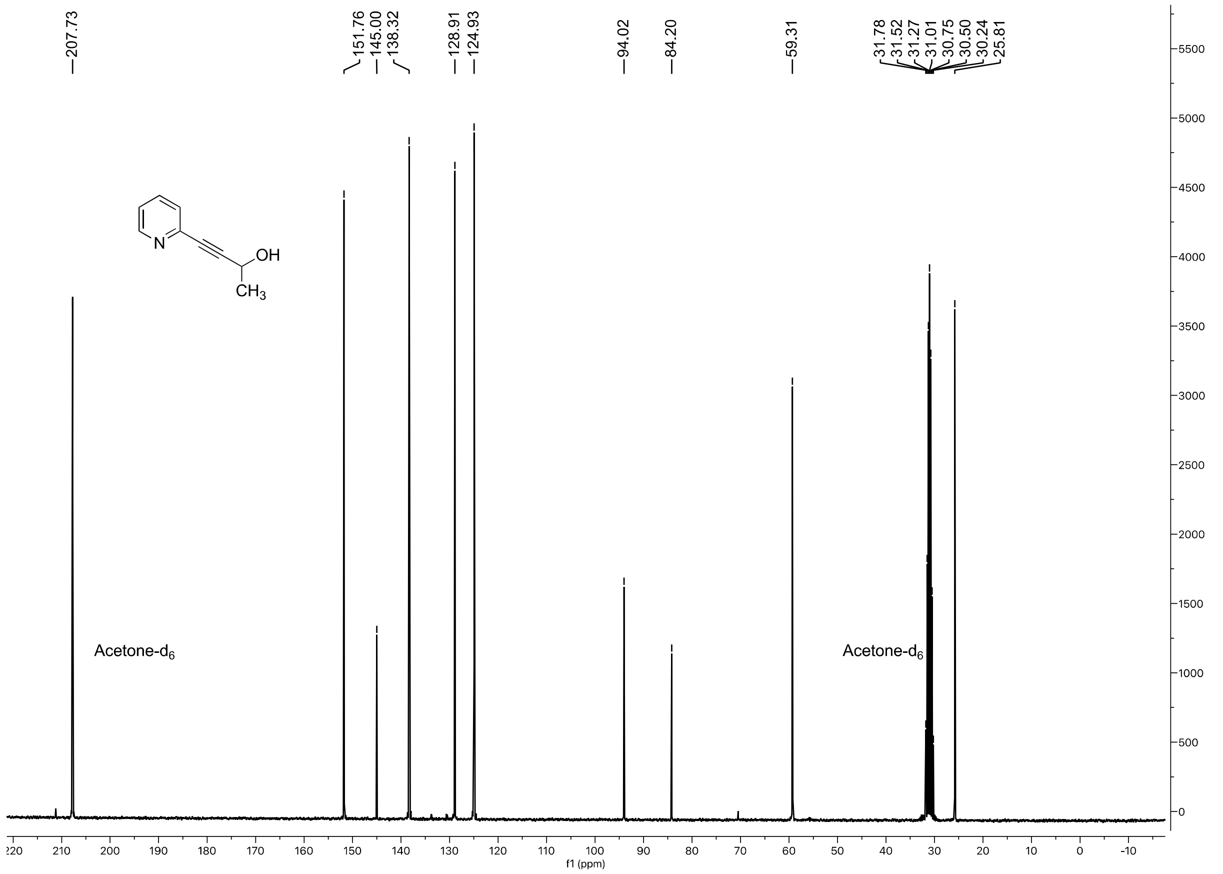

Figure S4. ^{13}C NMR spectrum of 4-(pyridine-2-yl)but-3-yn-2-ol (**3c**).

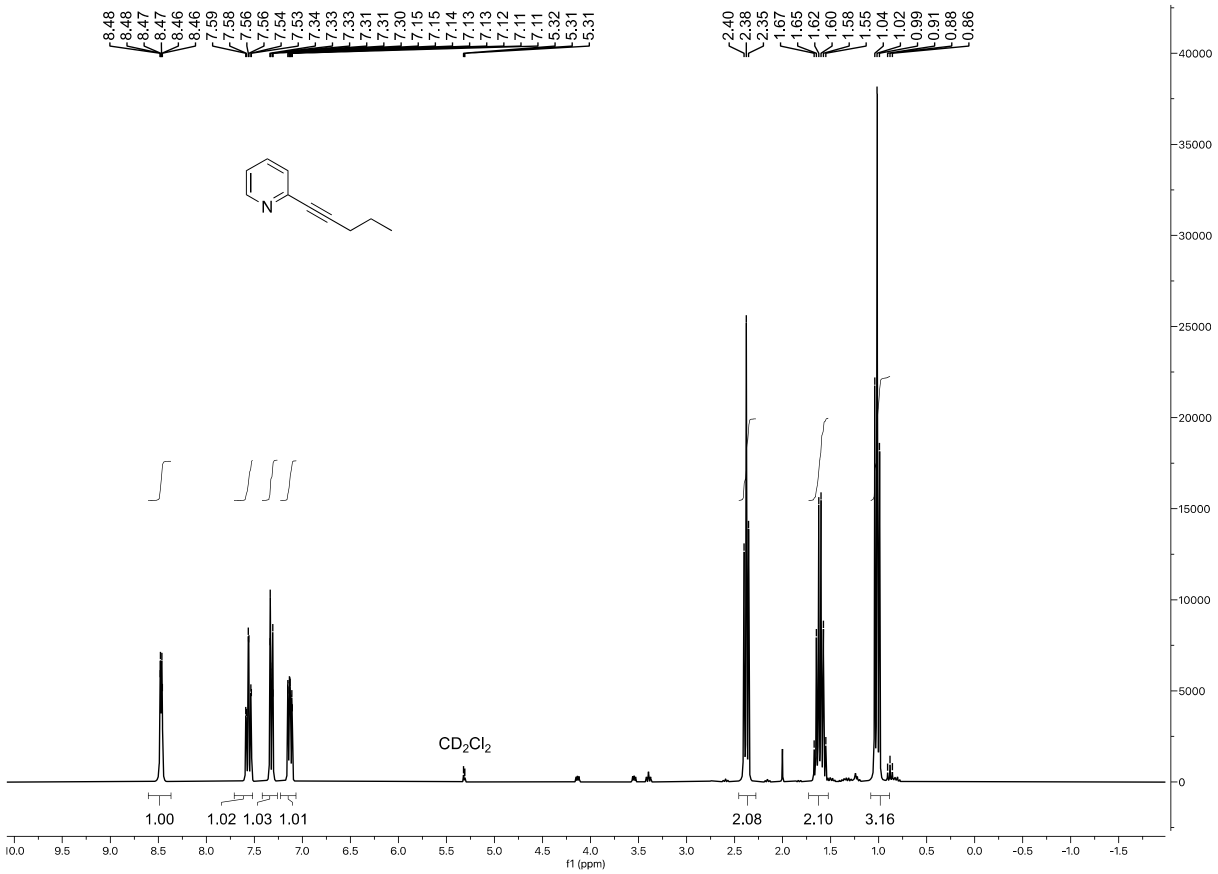

Figure S5. ^{1}H NMR spectrum of 2-(pent-1-yn-1-yl)pyridine (**4’a**).

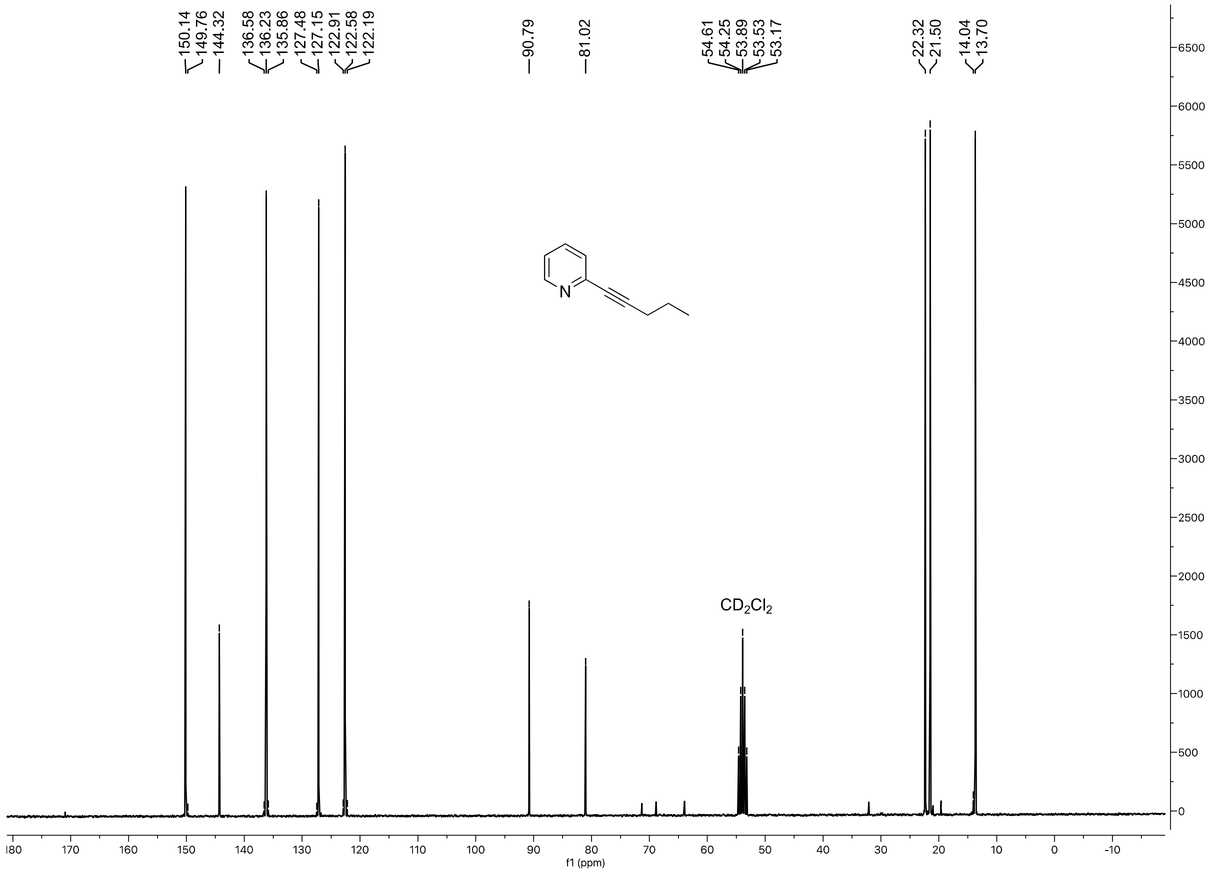

Figure S6. ^{13}C NMR spectrum of 2-(pent-1-yn-1-yl)pyridine (**4’a**).

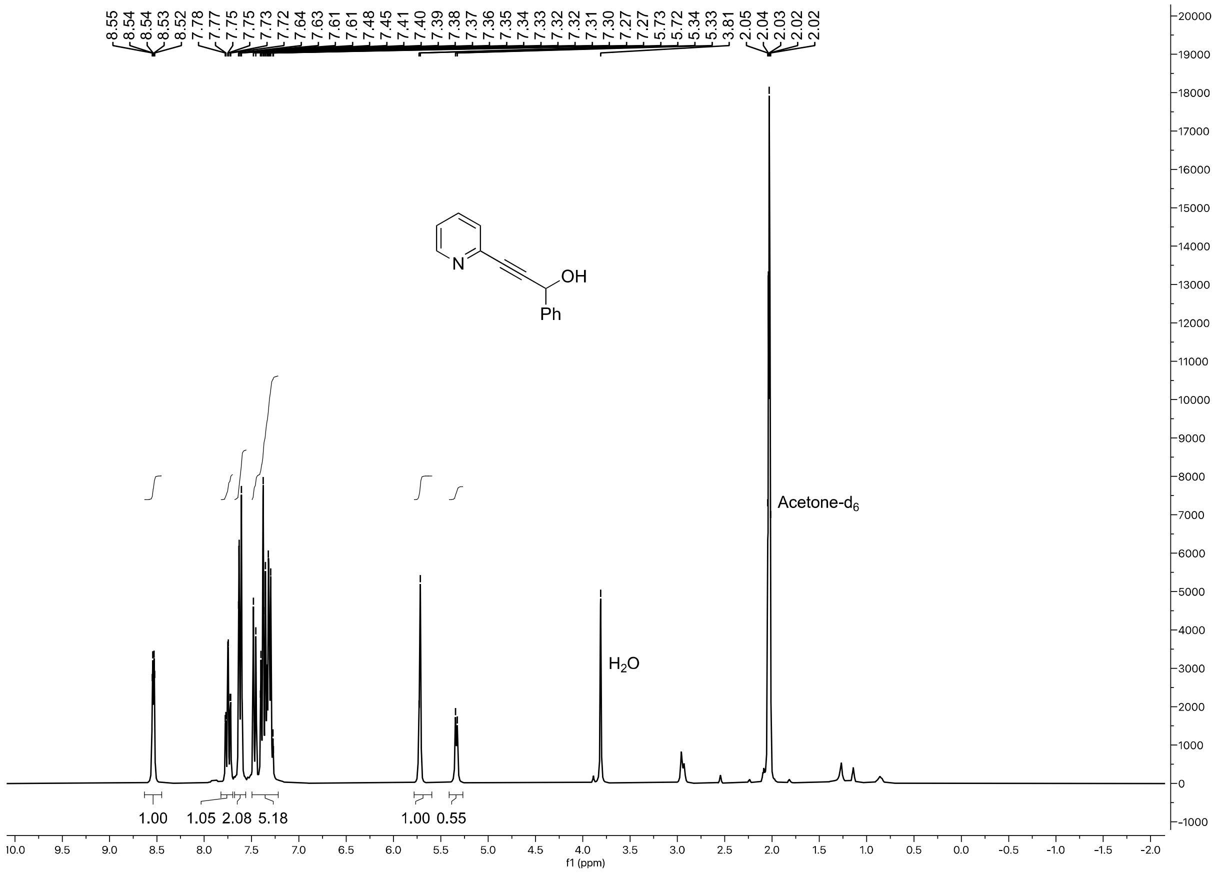

Figure S7. ^{1}H NMR spectrum of 1-phenyl-3-(pyridin-2-yl)prop-2-yn-1-ol (**3d**).

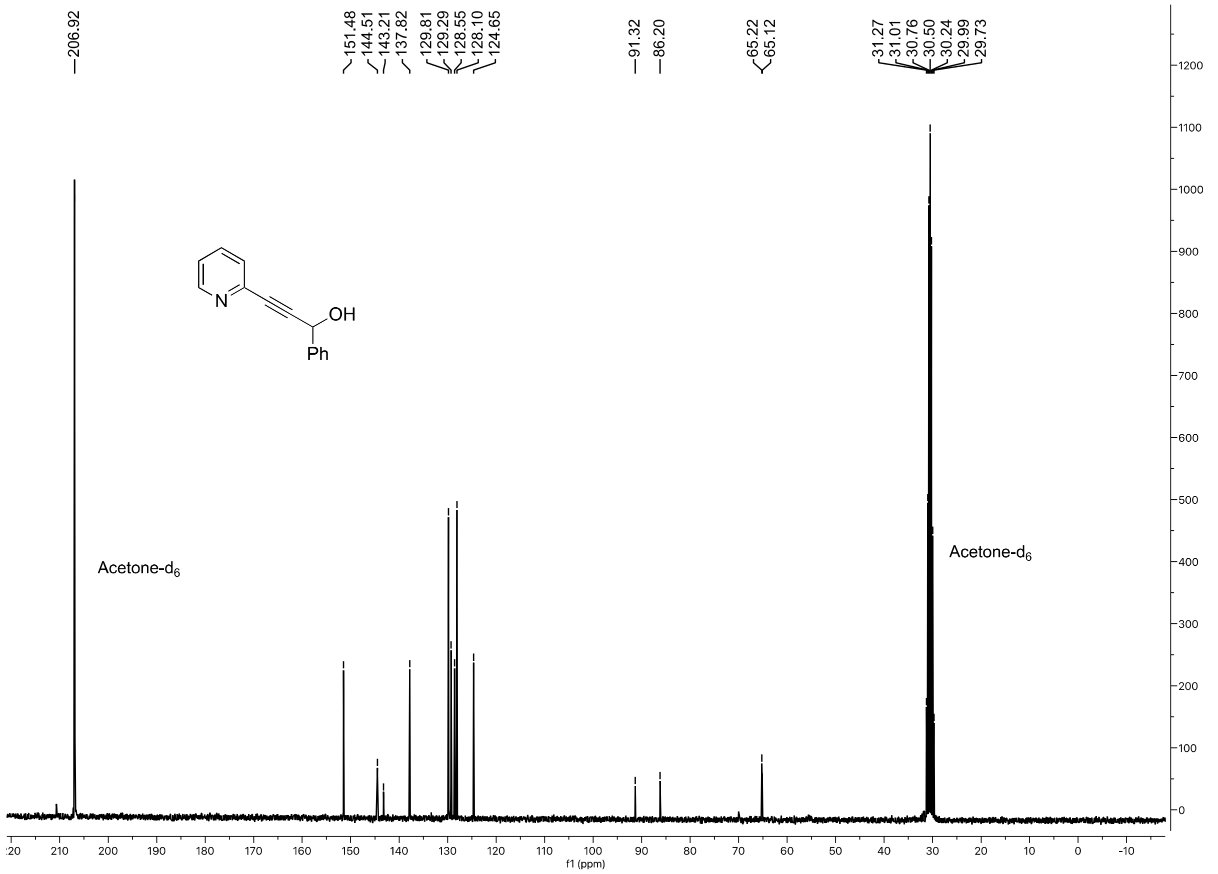

Figure S8. ^{13}C NMR spectrum of 1-phenyl-3-(pyridin-2-yl)prop-2-yn-1-ol (**3d**).

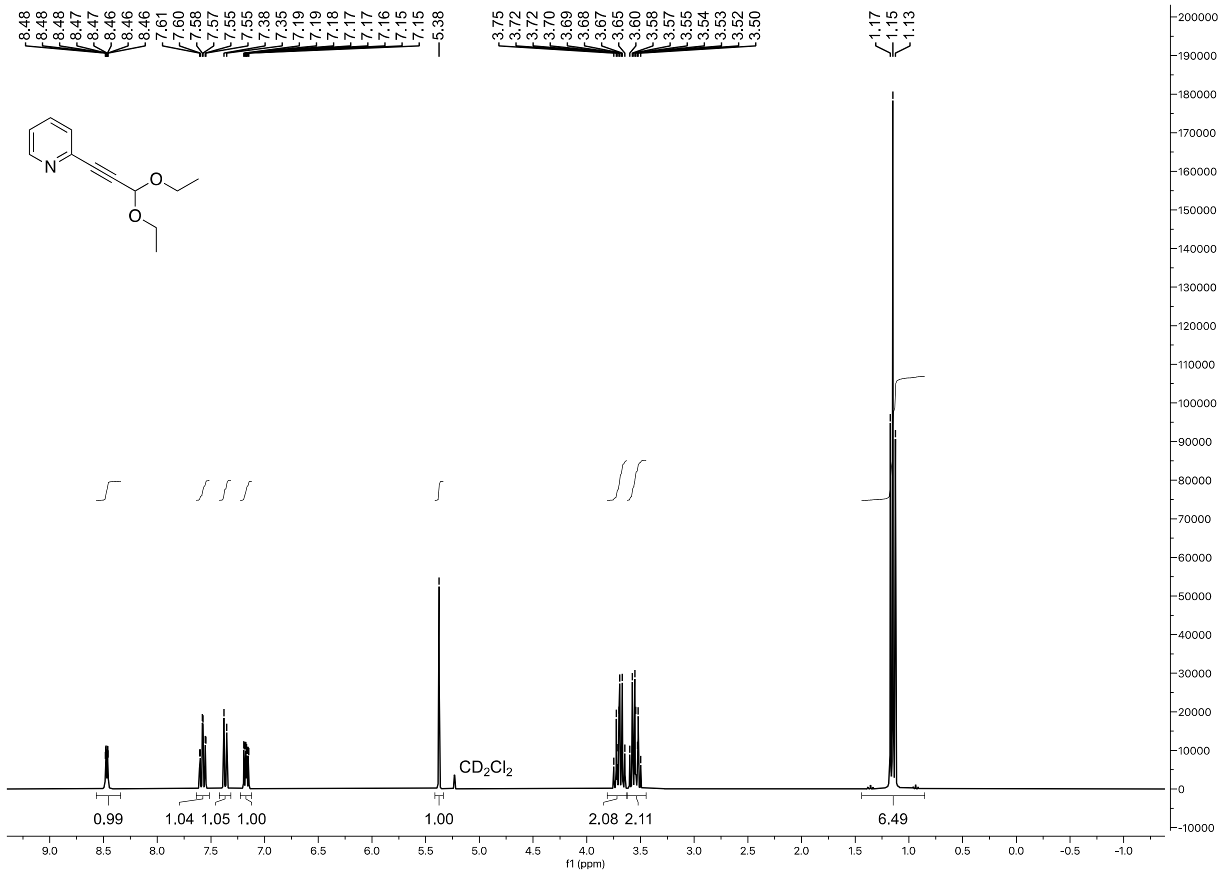

Figure S9. ^{1}H NMR spectrum of 2-(3,3-diethoxyprop-1-yn-1-yl)pyridine (**6**).

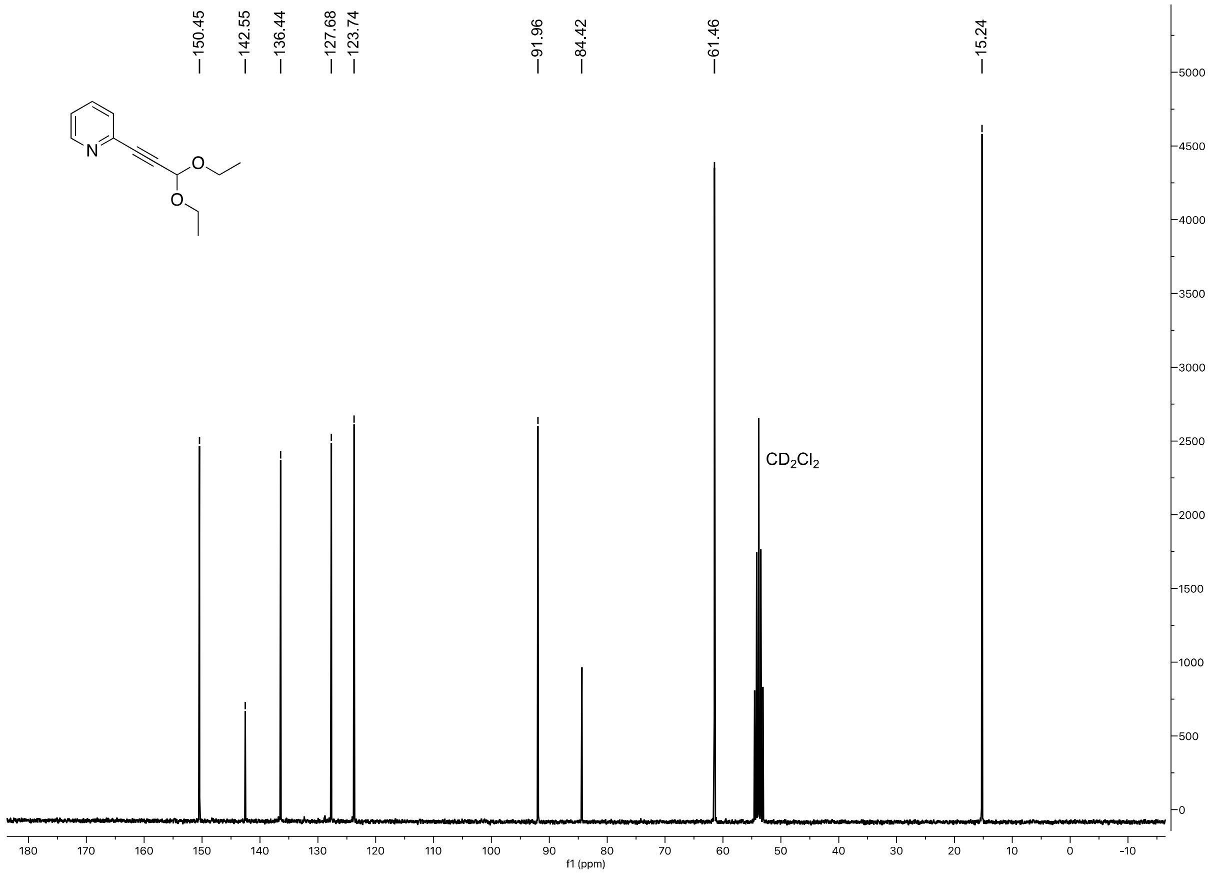

Figure S10. ^{13}C NMR spectrum of 2-(3,3-diethoxyprop-1-yn-1-yl)pyridine (**6**).

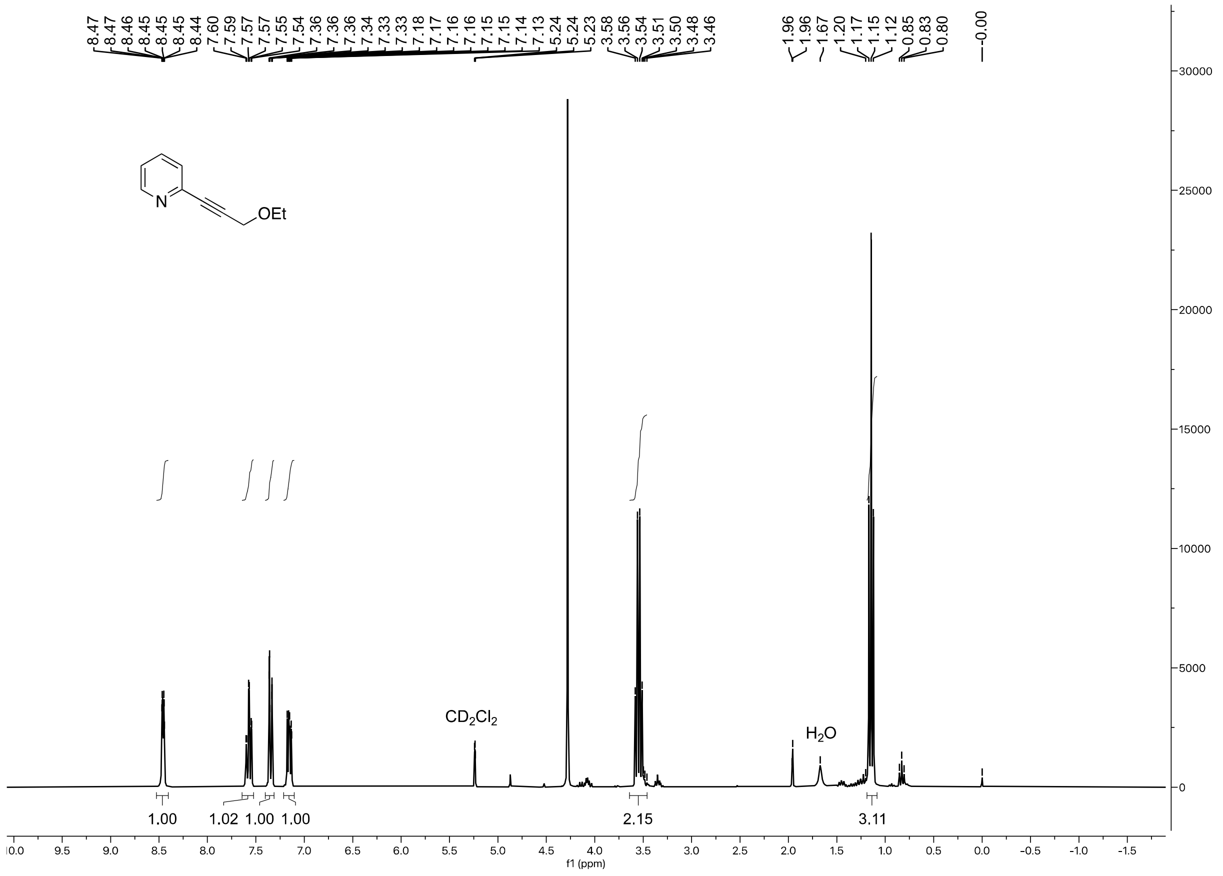

Figure S11. ^{1}H NMR spectrum of 2-(3-ethoxyprop-1-yn-1-yl)pyridine (**4b**).

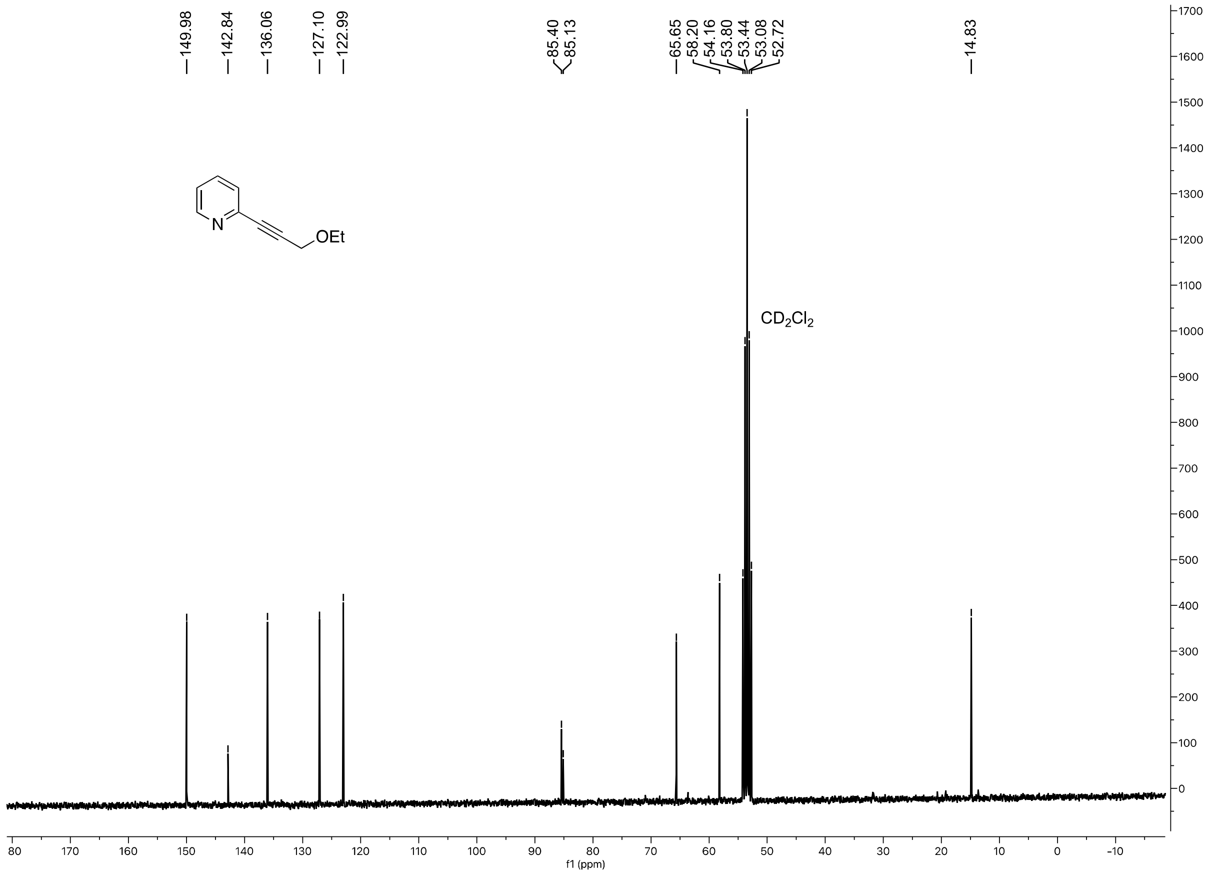

Figure S12. ^{13}C NMR spectrum of 2-(3-ethoxyprop-1-yn-1-yl)pyridine (**4b**).

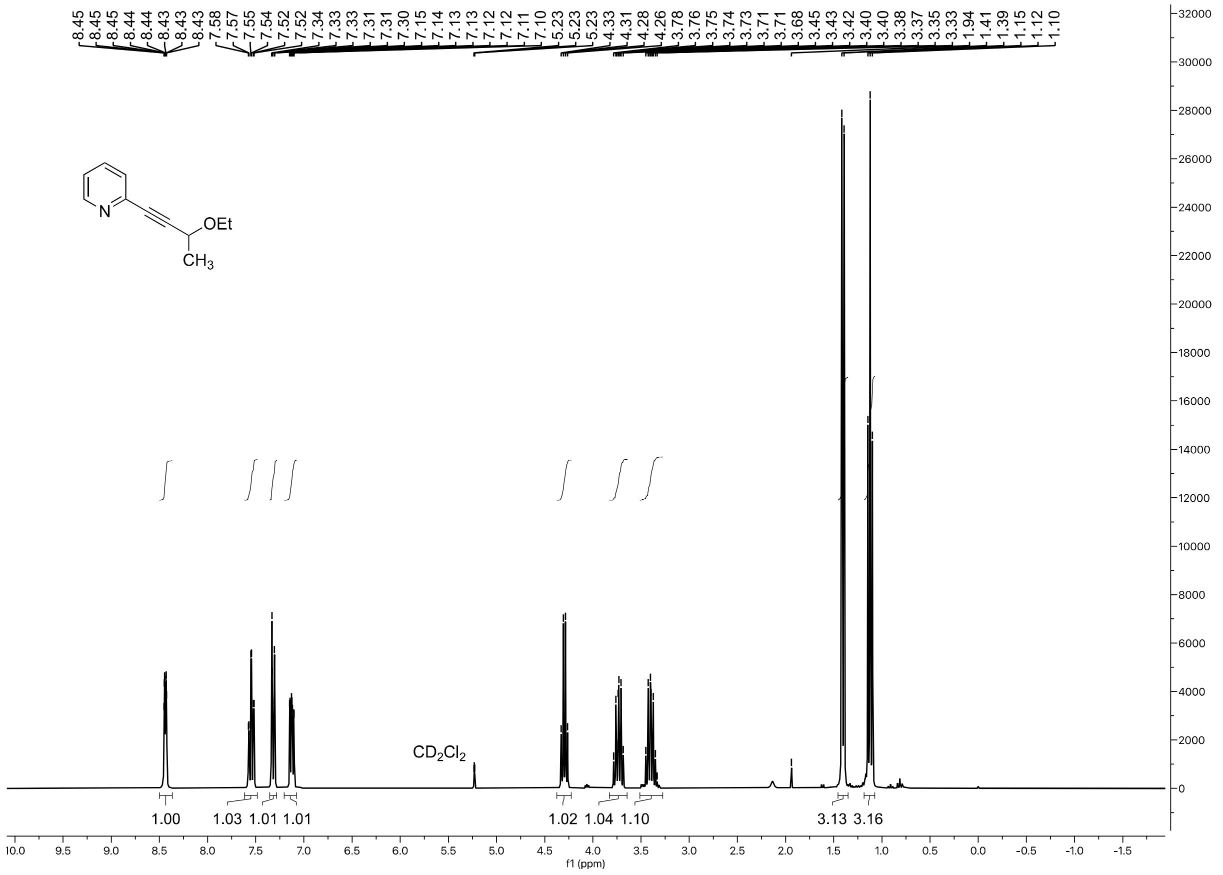

Figure S13. ^{1}H NMR spectrum of 2-(3-ethoxybut-1-yn-1-yl)pyridine (**4c**).

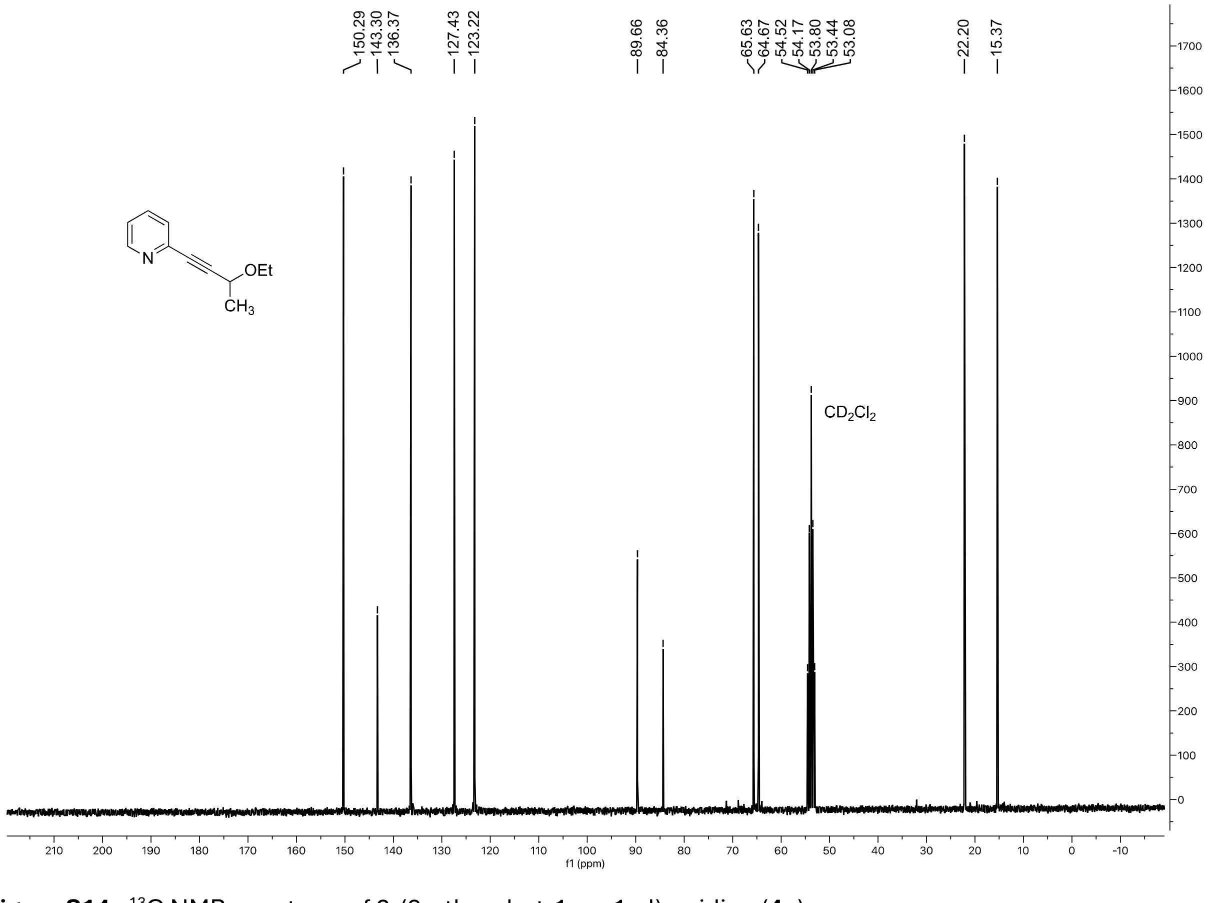

Figure S14. ^{13}C NMR spectrum of 2-(3-ethoxybut-1-yn-1-yl)pyridine (**4c**).

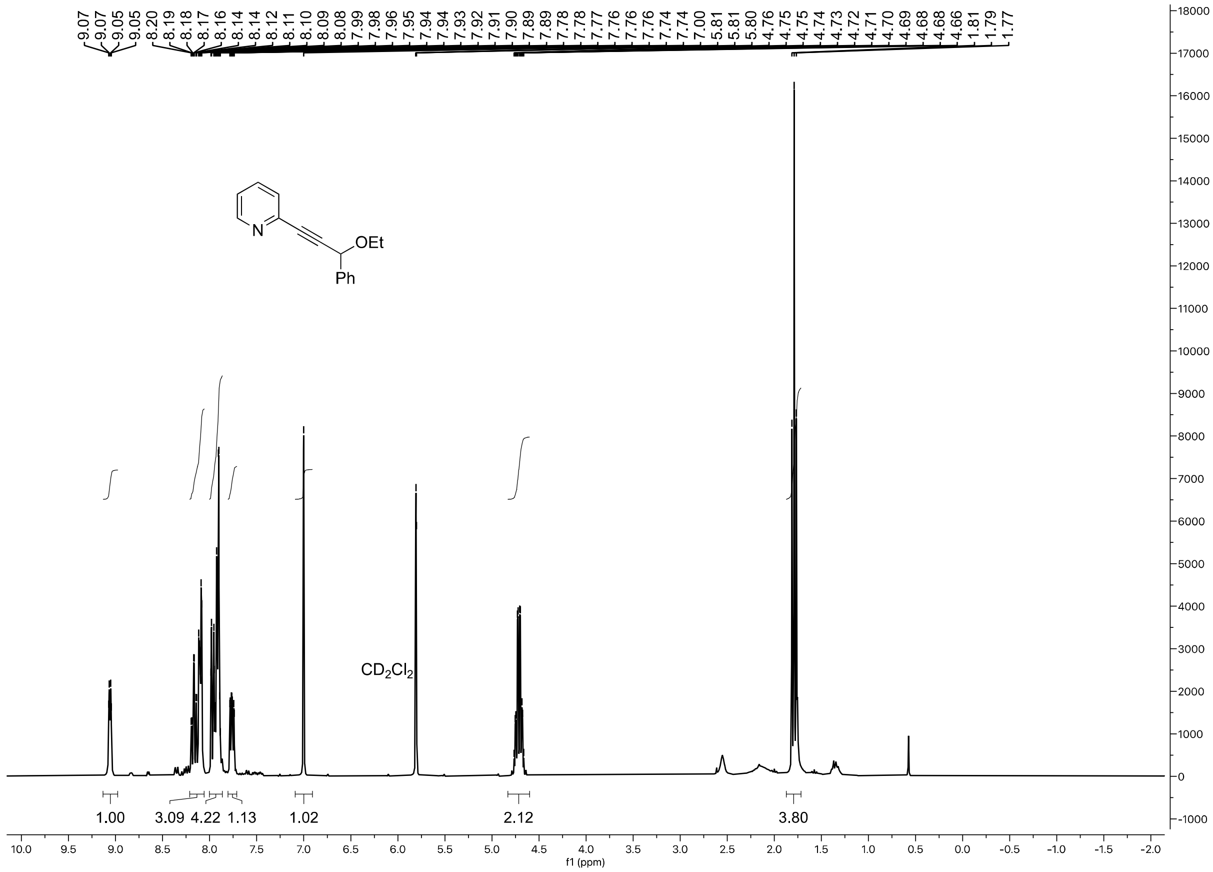

Figure S15. ^{1}H NMR spectrum of 2-(3-ethoxy-3-phenylprop-1-yn-1-yl)pyridine (**4d**).

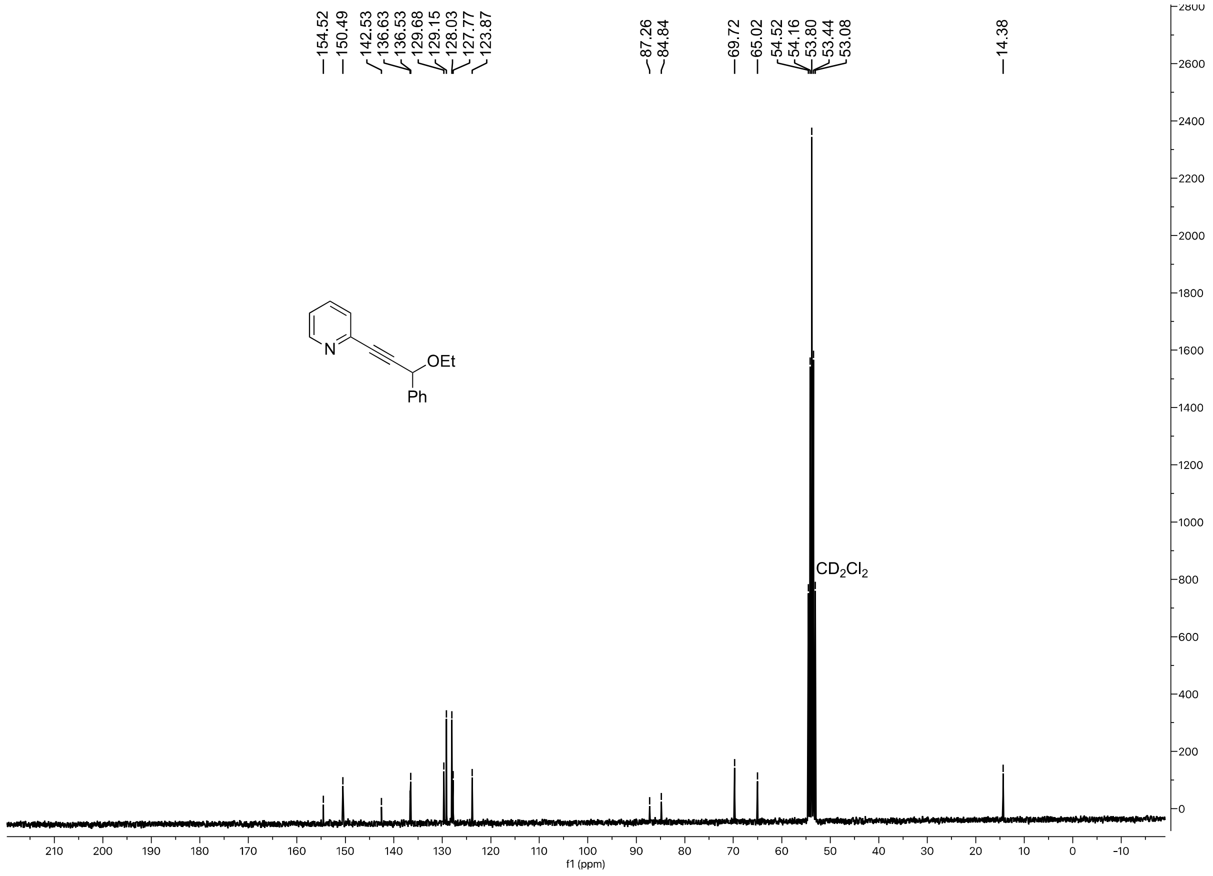

Figure S16. ^{13}C NMR spectrum of 2-(3-ethoxy-3-phenylprop-1-yn-1-yl)pyridine (**4d**).

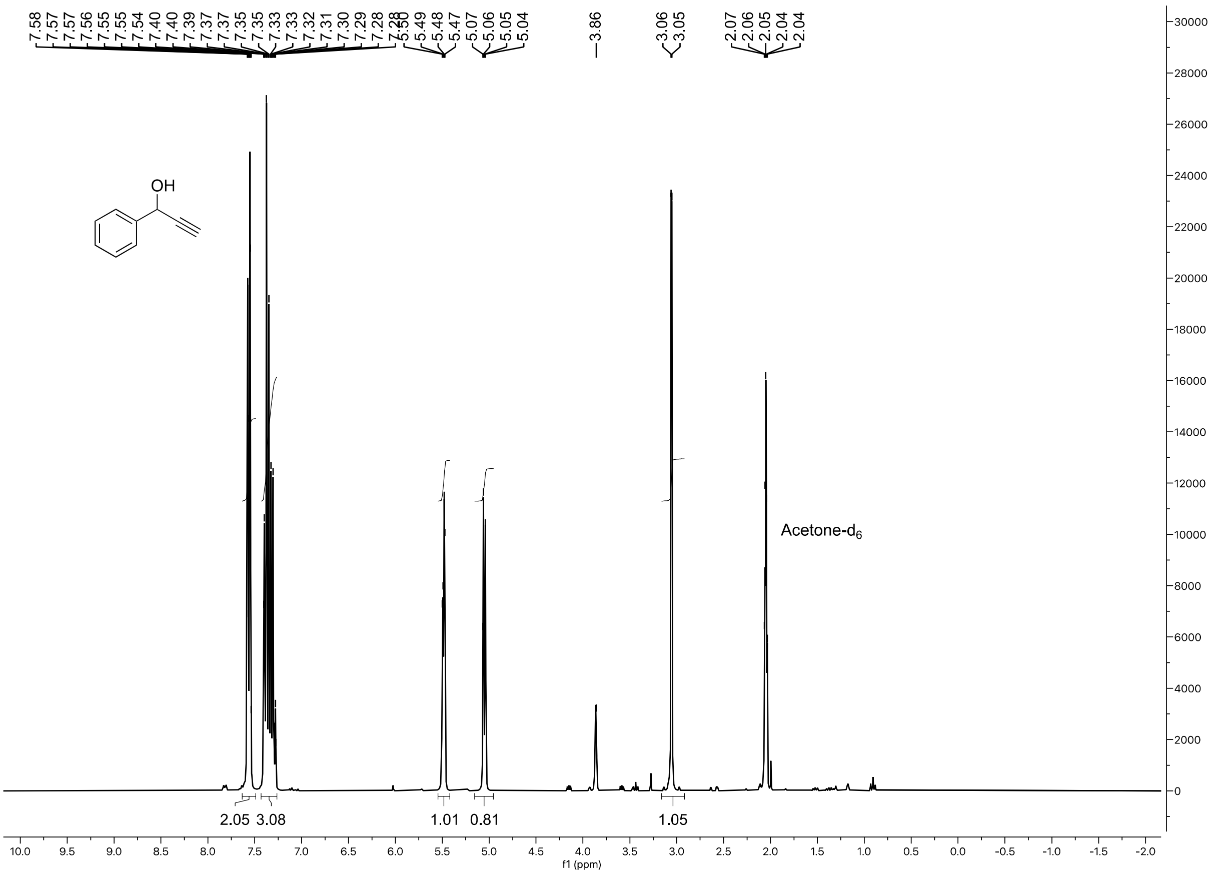

Figure S17. ^{1}H NMR spectrum of 1-phenylprop-2-yn-1-ol (**2d**).

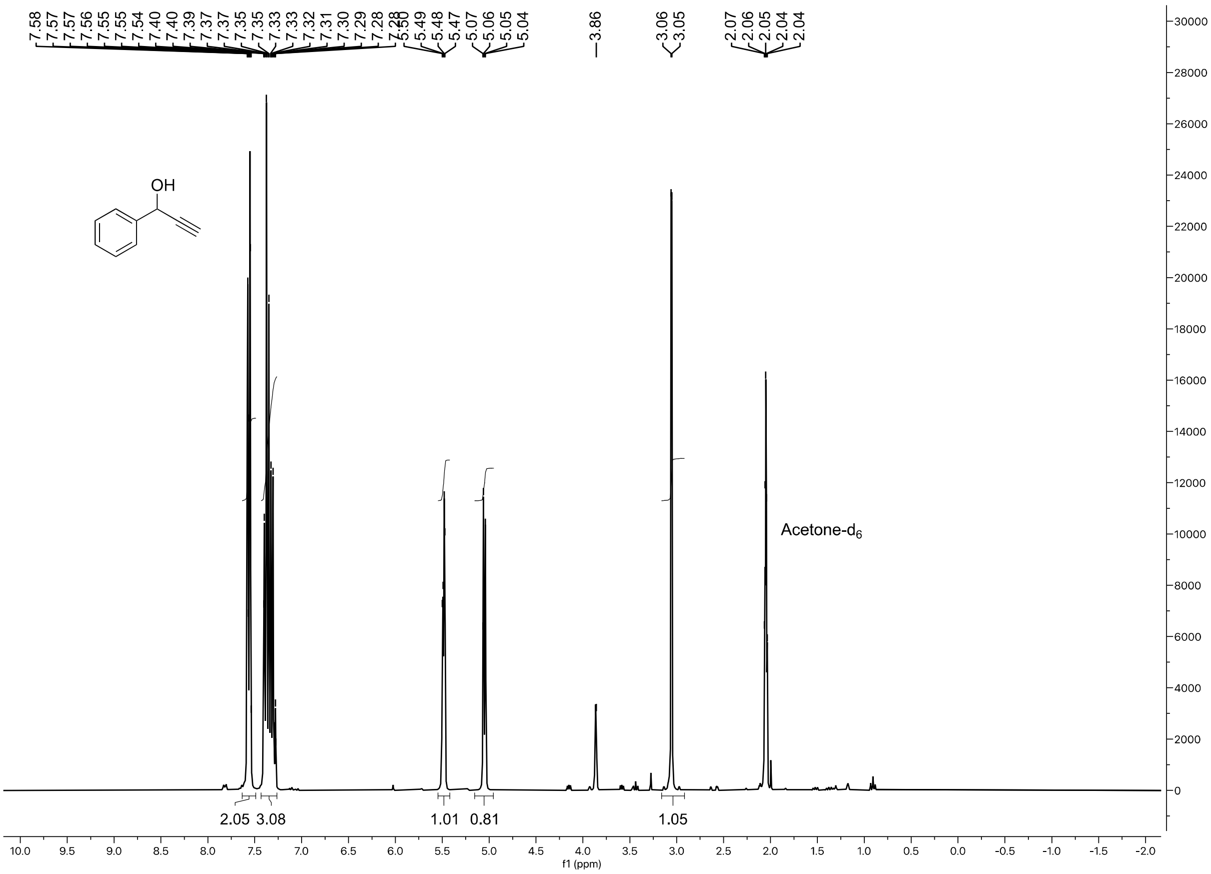

Figure S18. ^{13}C NMR spectrum of 1-phenylprop-2-yn-1-ol (**2d**).

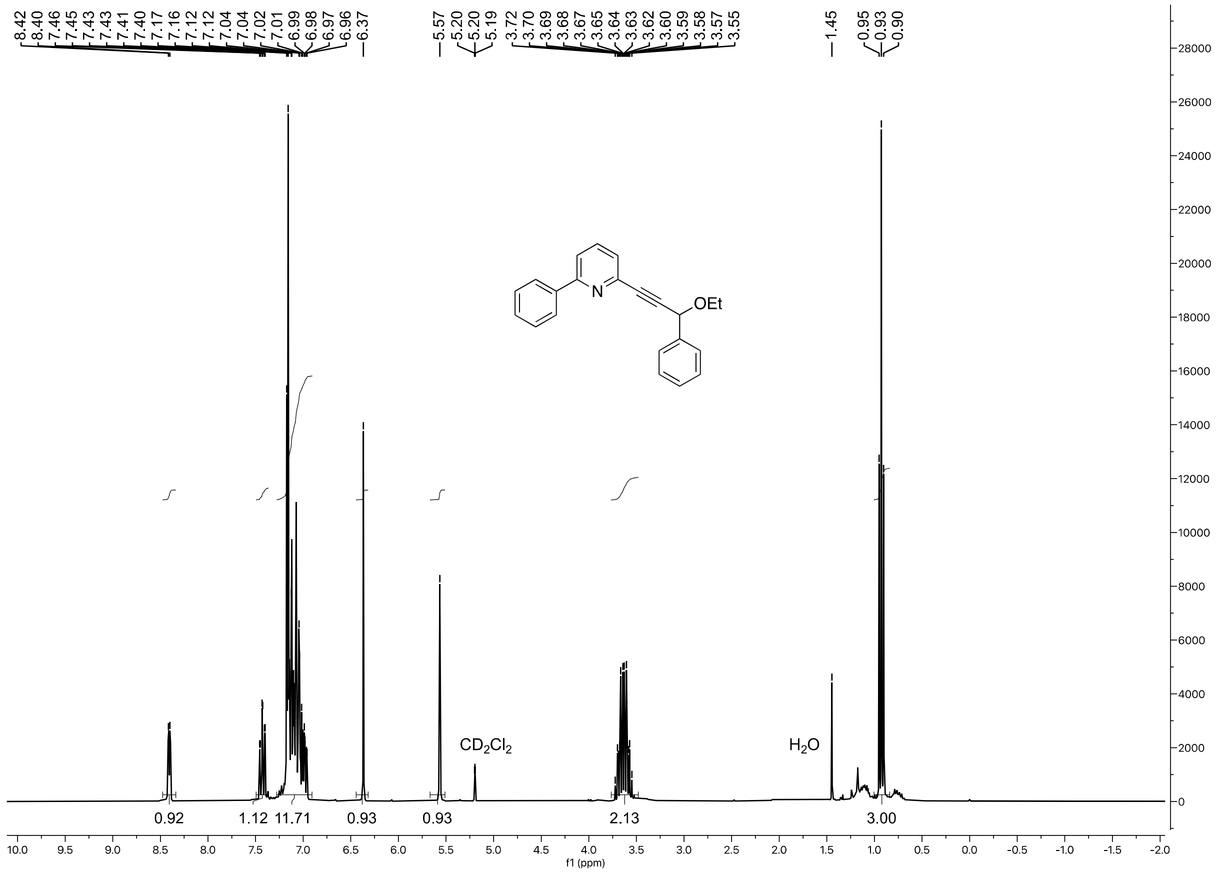

Figure S19. ^{1}H NMR spectrum of 2-(3-ethoxy-3-phenylprop-1-yn-1-yl)-6-phenylpyridine (**8**).

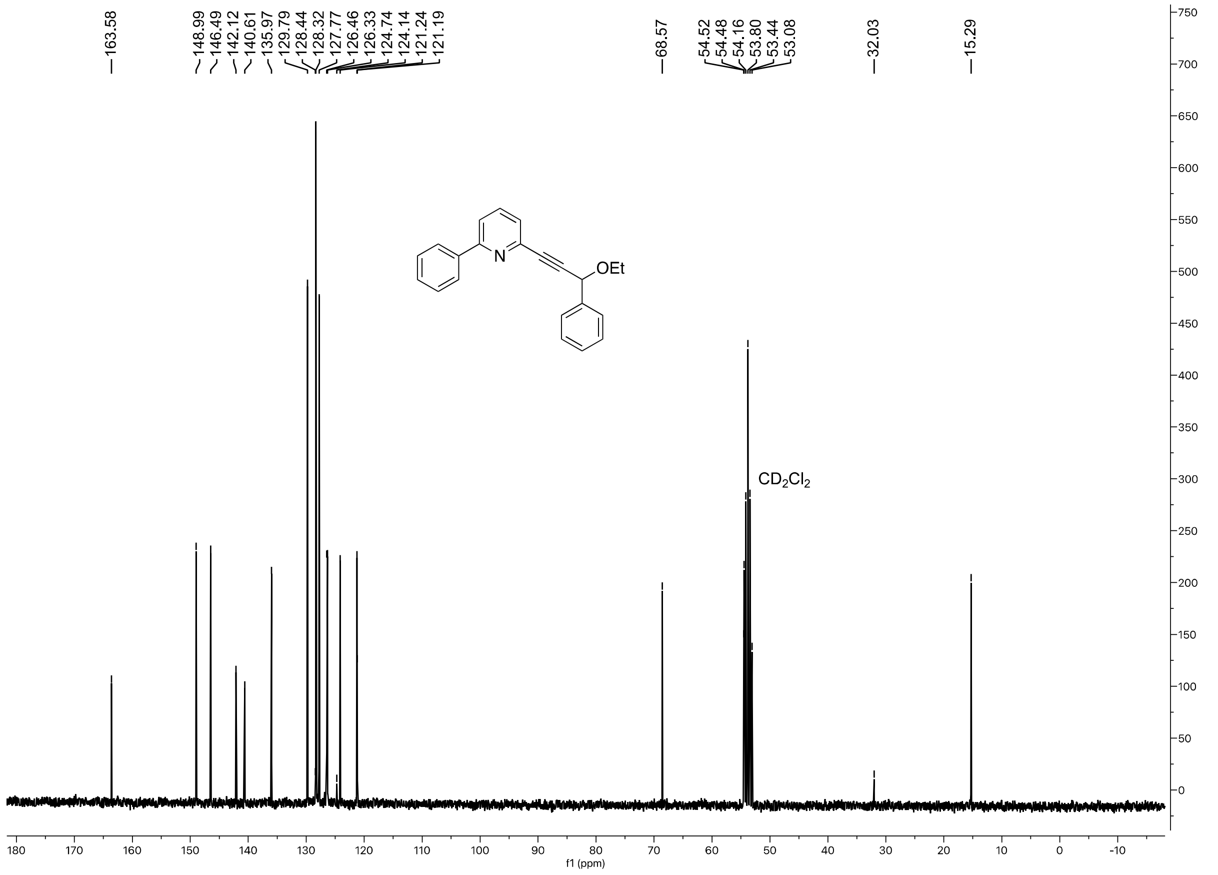

Figure S20. ^{13}C NMR spectrum of 2-(3-ethoxy-3-phenylprop-1-yn-1-yl)-6-phenylpyridine (**8**).

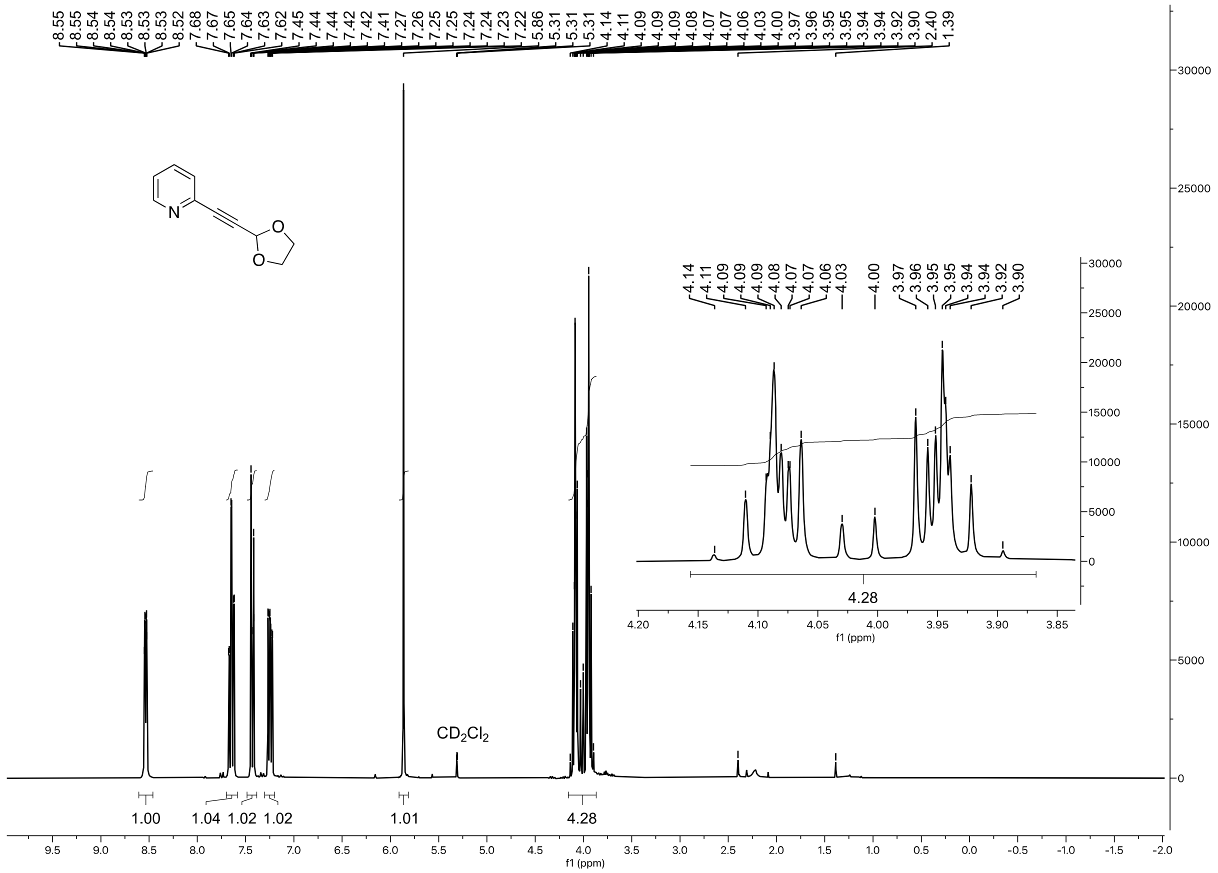

Figure S21. ^{1}H NMR spectrum of 2-((1,3-dioxolan-2-yl)ethynyl)pyridine (**4e**).

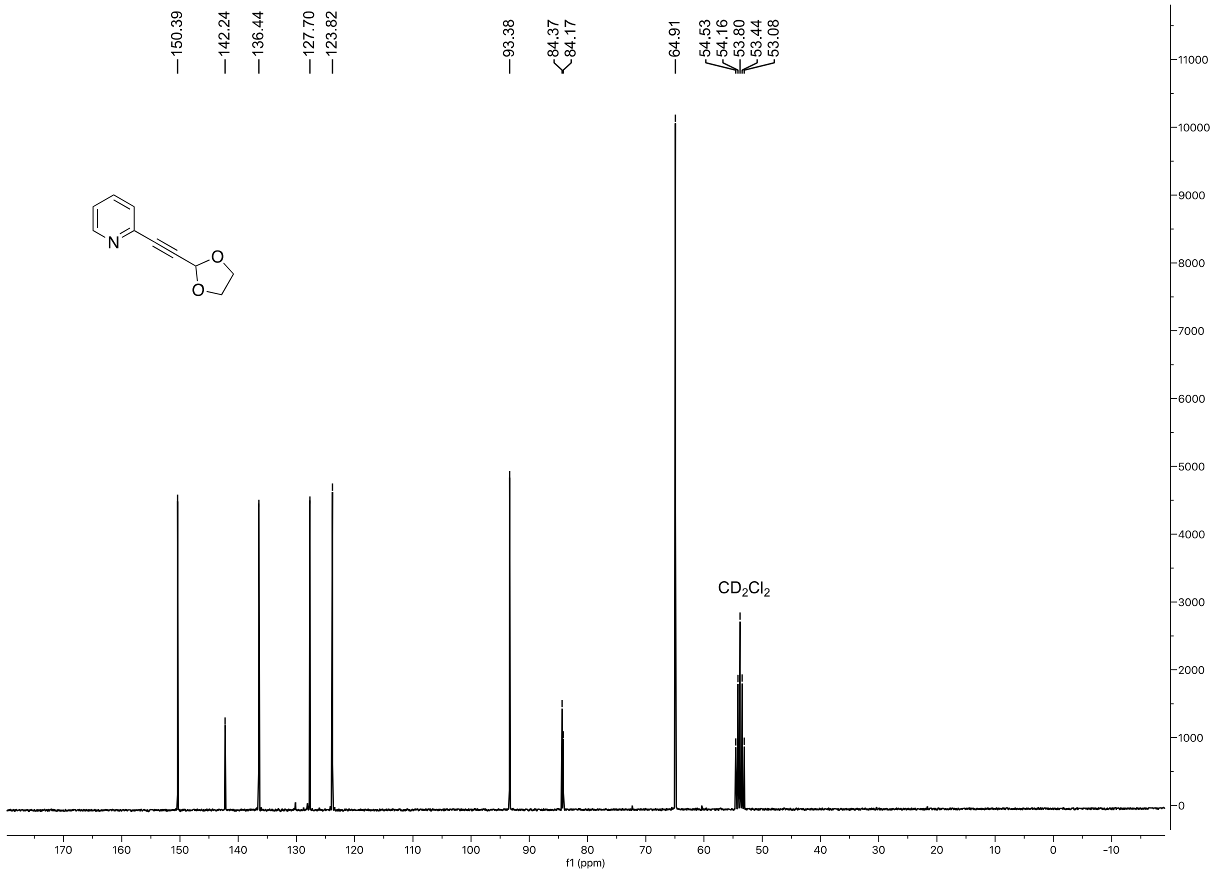

Figure S22. ^{13}C NMR spectrum of 2-((1,3-dioxolan-2-yl)ethynyl)pyridine (**4e**).

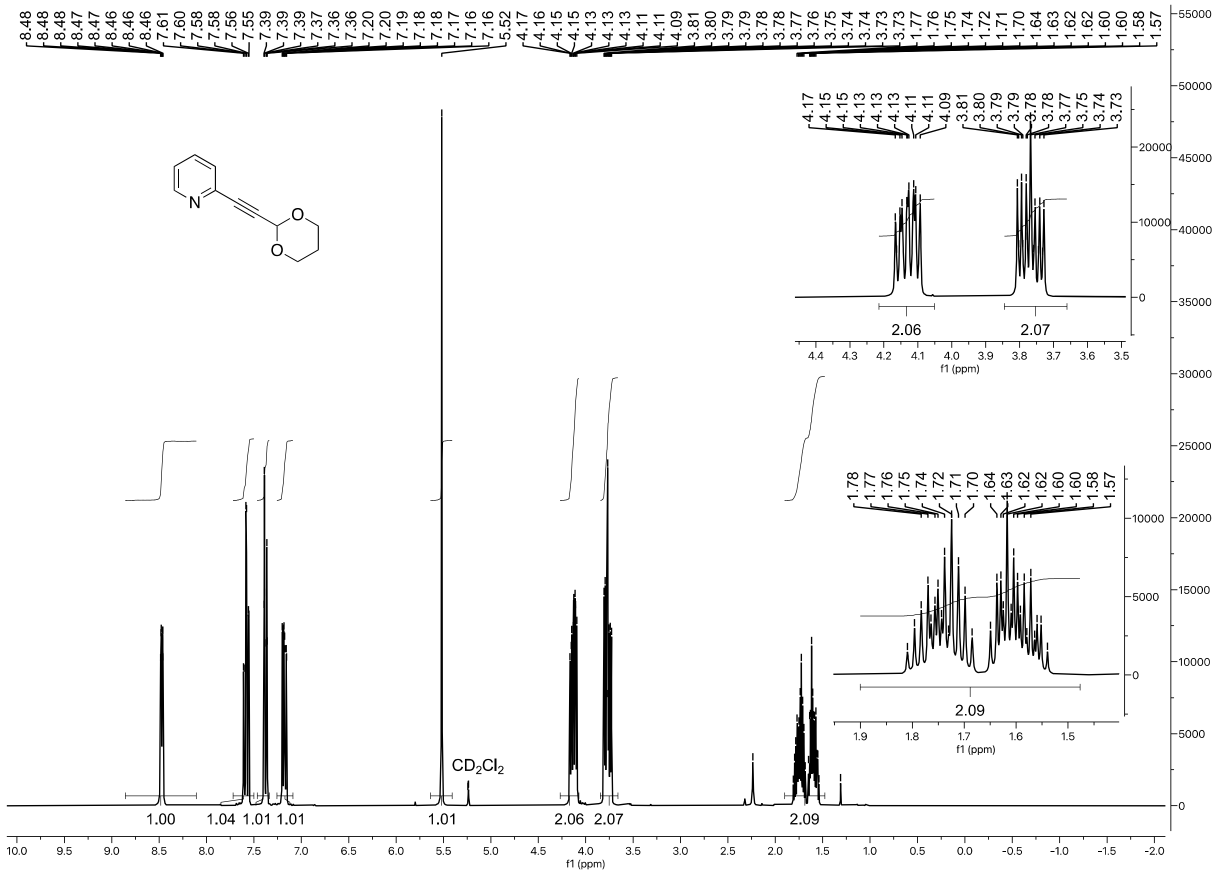

Figure S23. ^{1}H NMR spectrum of 2-((1,3-dioxan-2-yl)ethynyl)pyridine (**4f**).

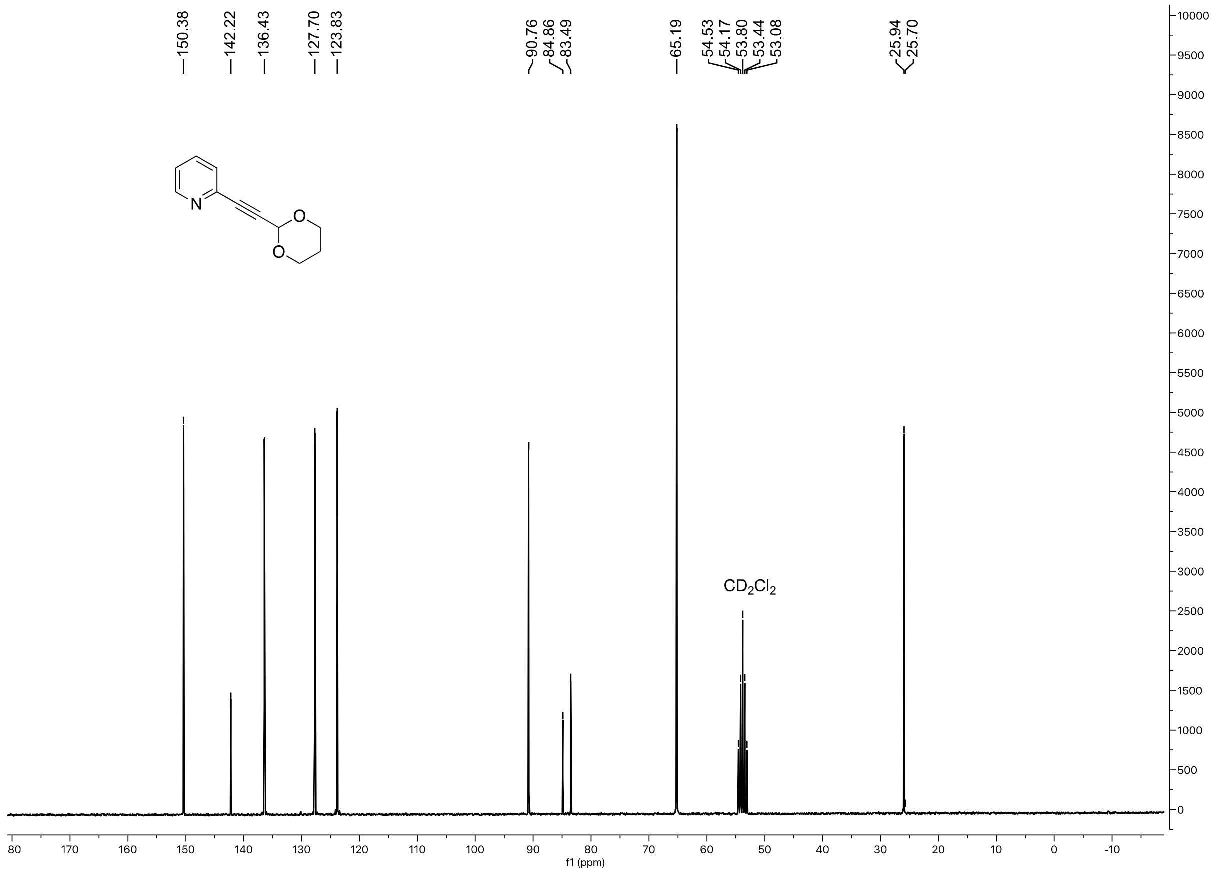

Figure S24. ^{13}C NMR spectrum of 2-((1,3-dioxan-2-yl)ethynyl)pyridine (**4f**).

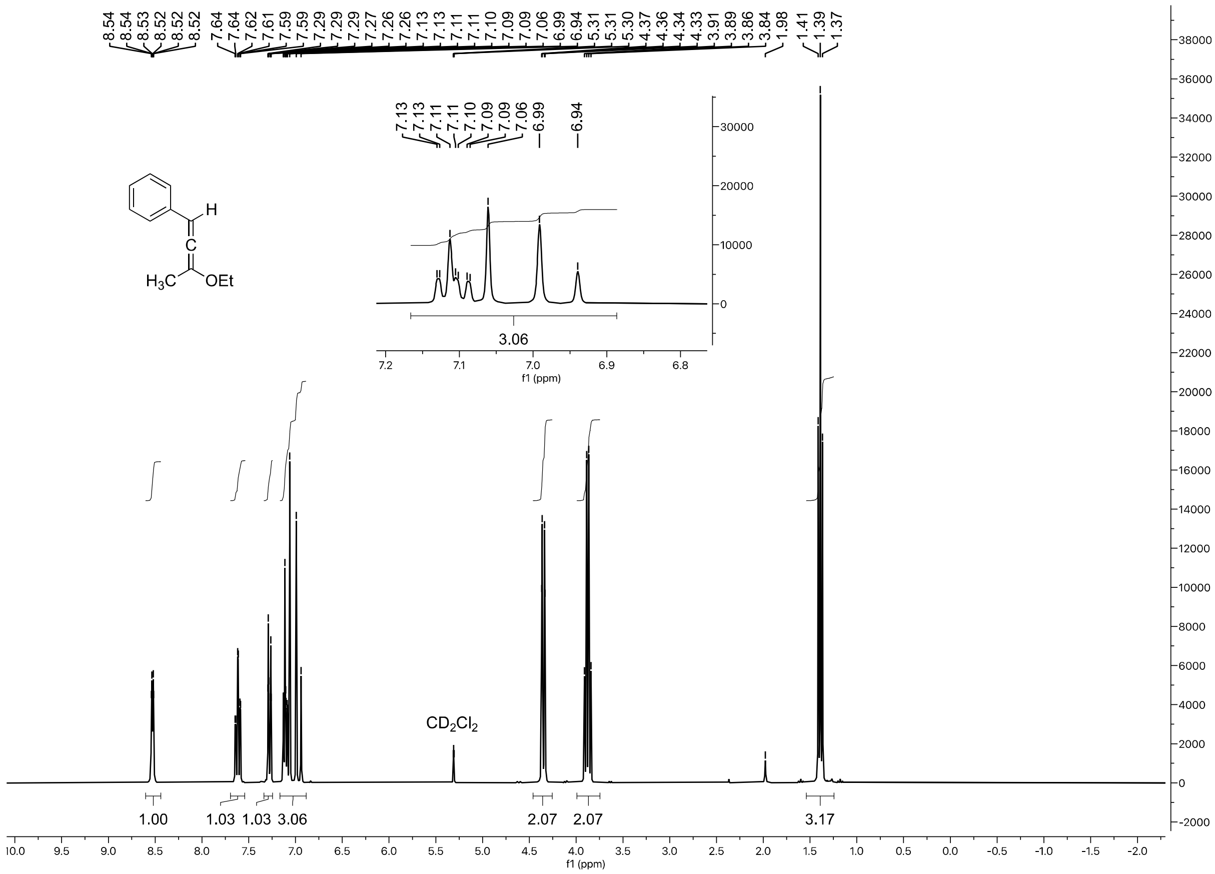

Figure S25. ^{1}H NMR spectrum of 2-(3-ethoxybuta-1,2-dien-1-yl)pyridine (**4g**).

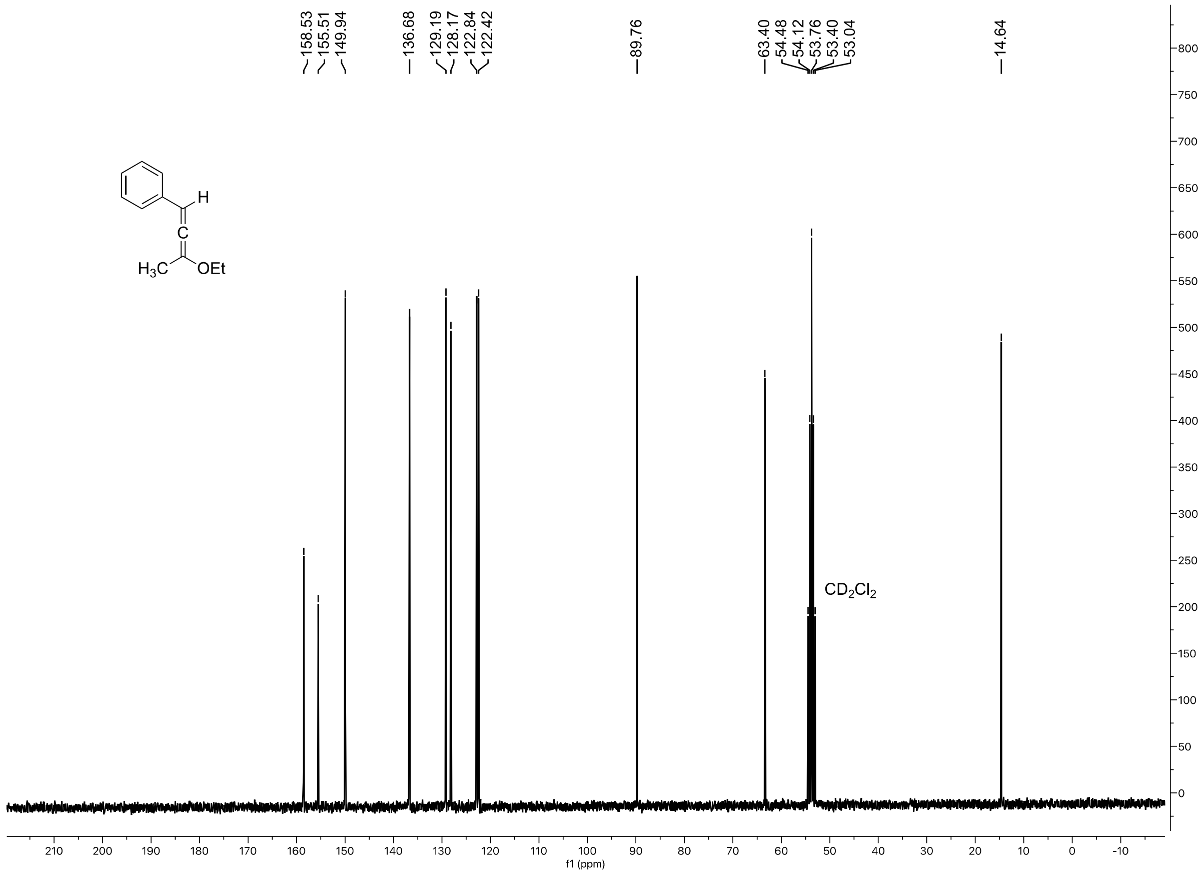

Figure S26. ^{13}C NMR spectrum of 2-(3-ethoxybuta-1,2-dien-1-yl)pyridine (**4g**).

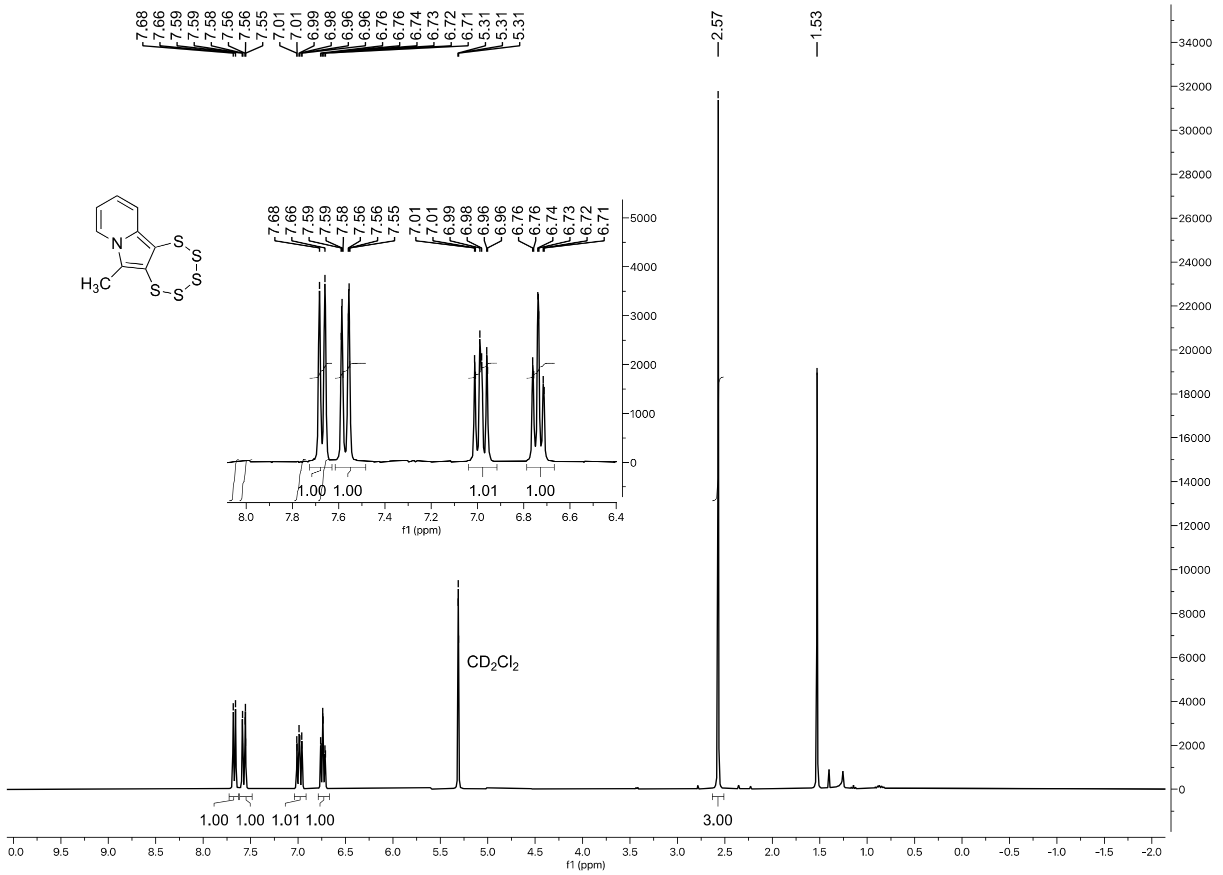

Figure S27. ^{1}H NMR spectrum of 6-methyl-[1,2,3,4,5]pentathiepino[6,7-a]indolizine (**5c**).

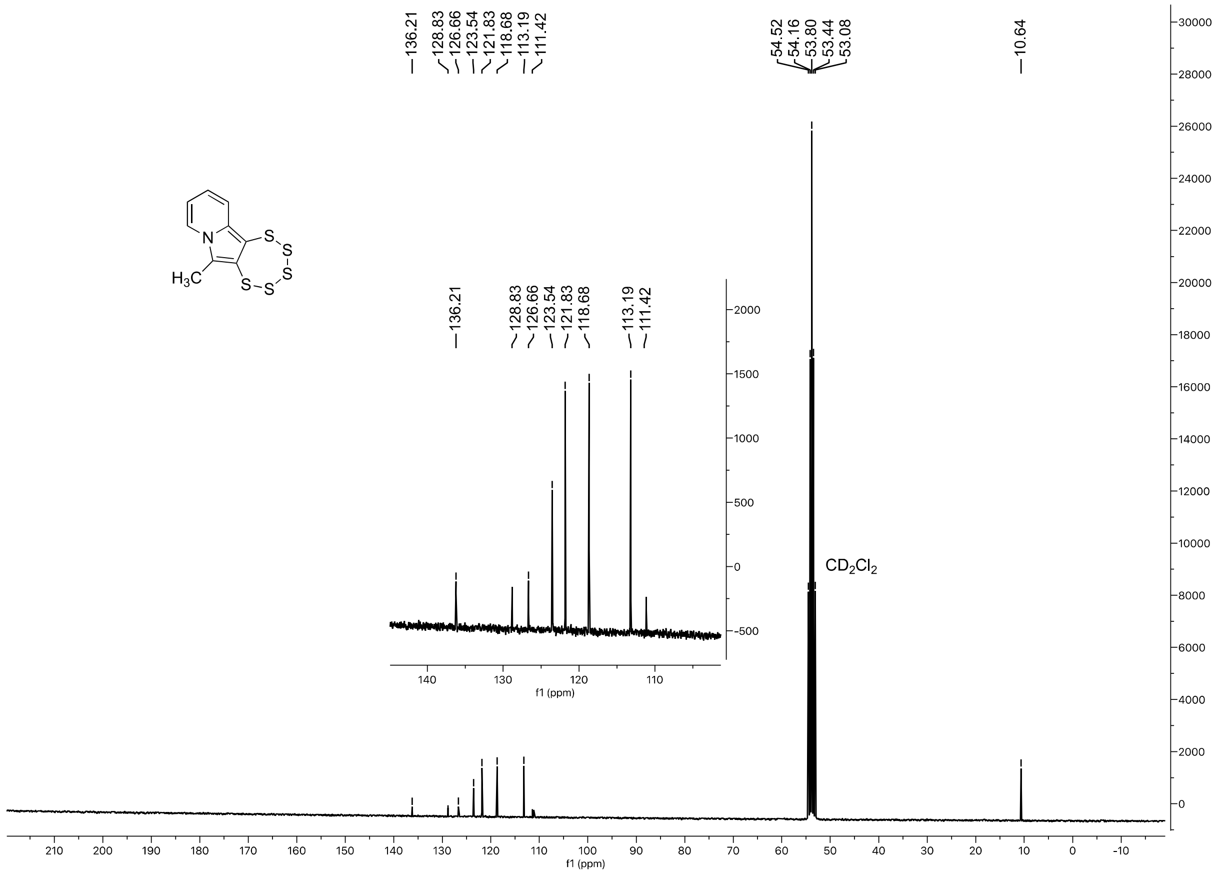

Figure S28. ^{13}C NMR spectrum of 6-methyl-[1,2,3,4,5]pentathiepino[6,7-a]indolizine (**5c**).

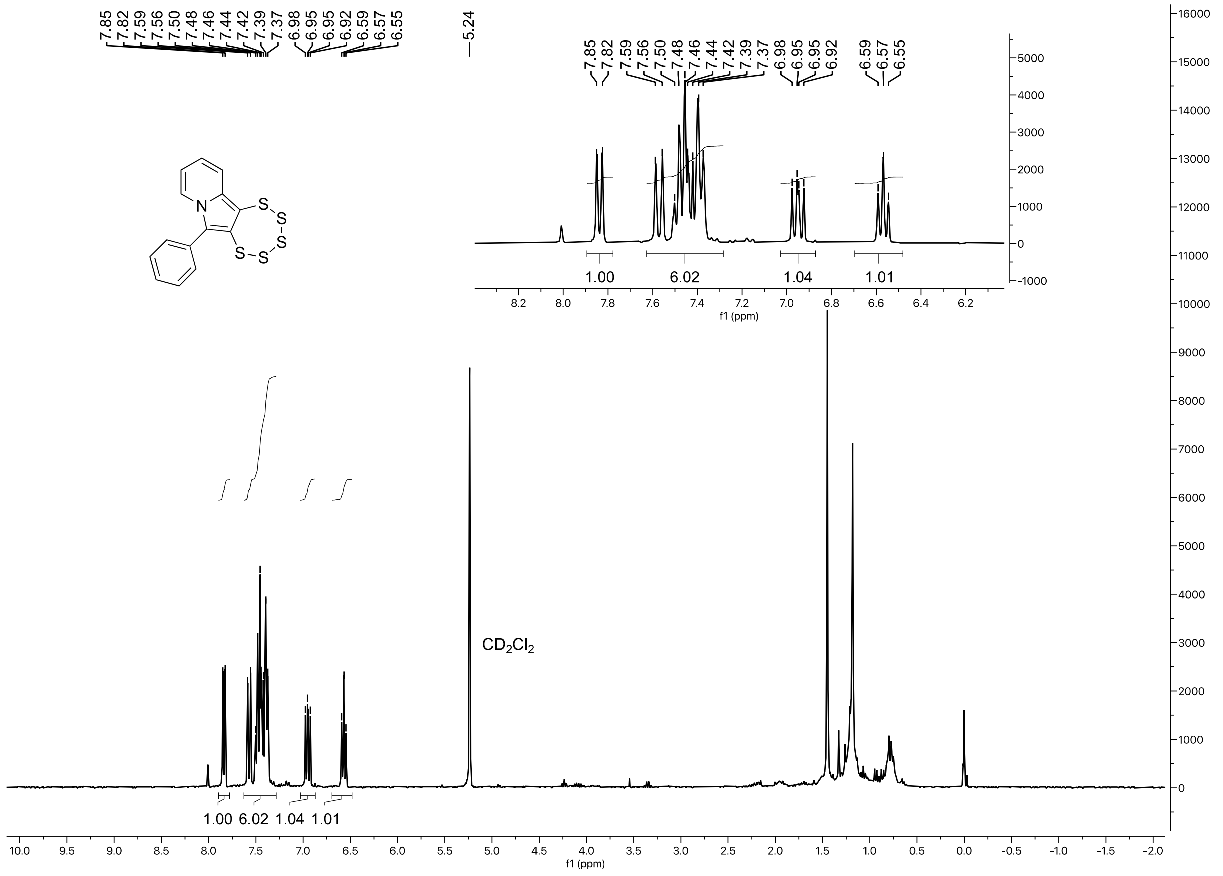

Figure S29. ^{1}H NMR spectrum of 6-phenyl-[1,2,3,4,5]pentathiepino[6,7-a]indolizine (**5d**).

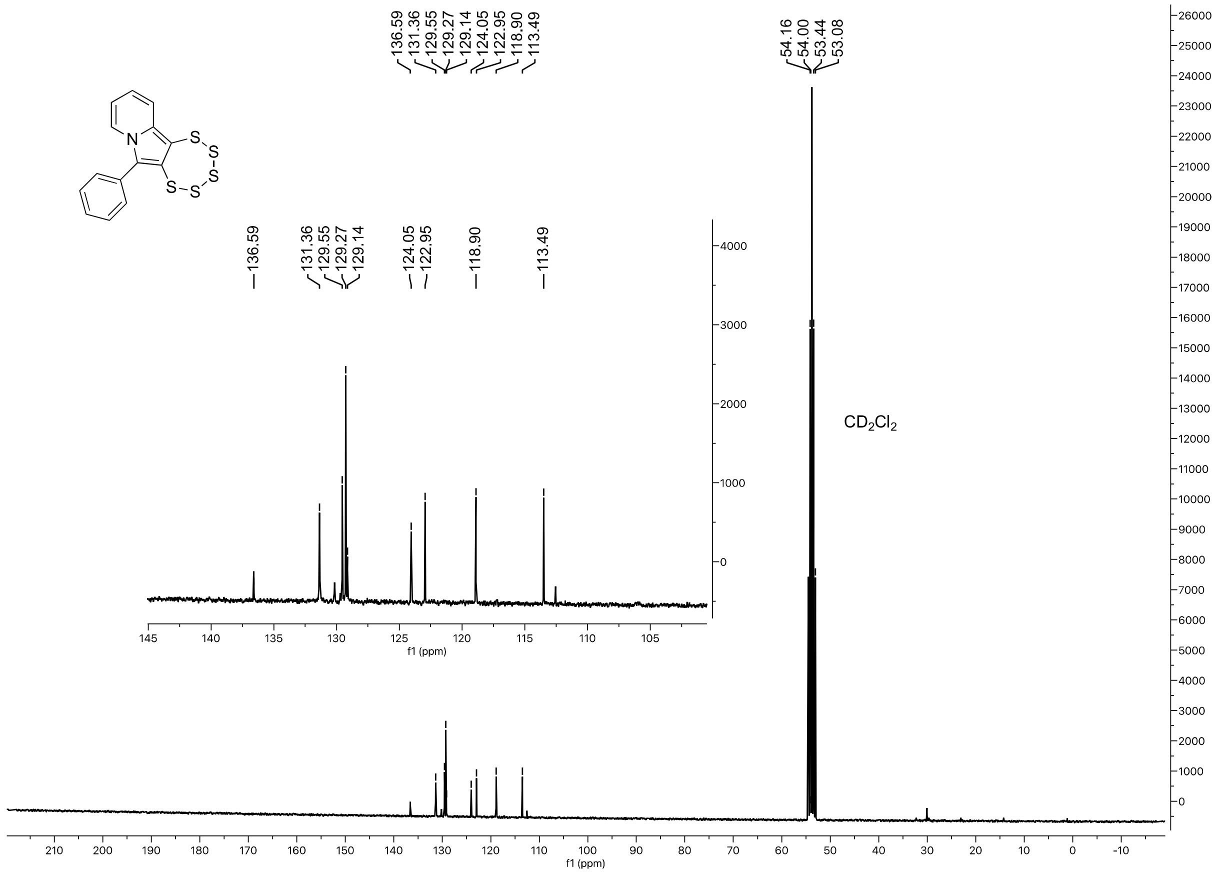

Figure S30. ^{13}C NMR spectrum of6-phenyl-[1,2,3,4,5]pentathiepino[6,7-a]indolizine (**5d**).

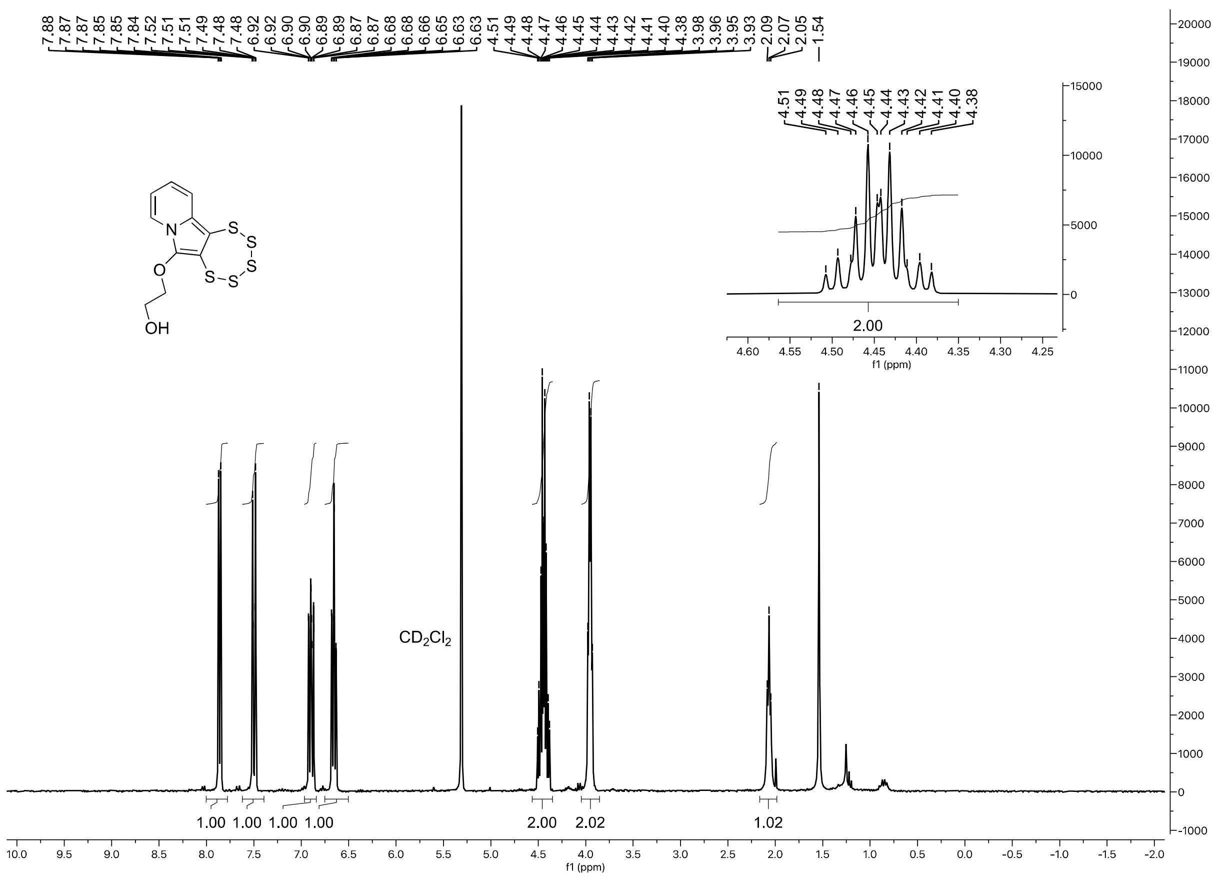

Figure S31. ^{1}H NMR spectrum of 2-([1,2,3,4,5]pentathiepino[6,7-a]indolizin-6-yloxy)ethan-1-ol (**5e**).

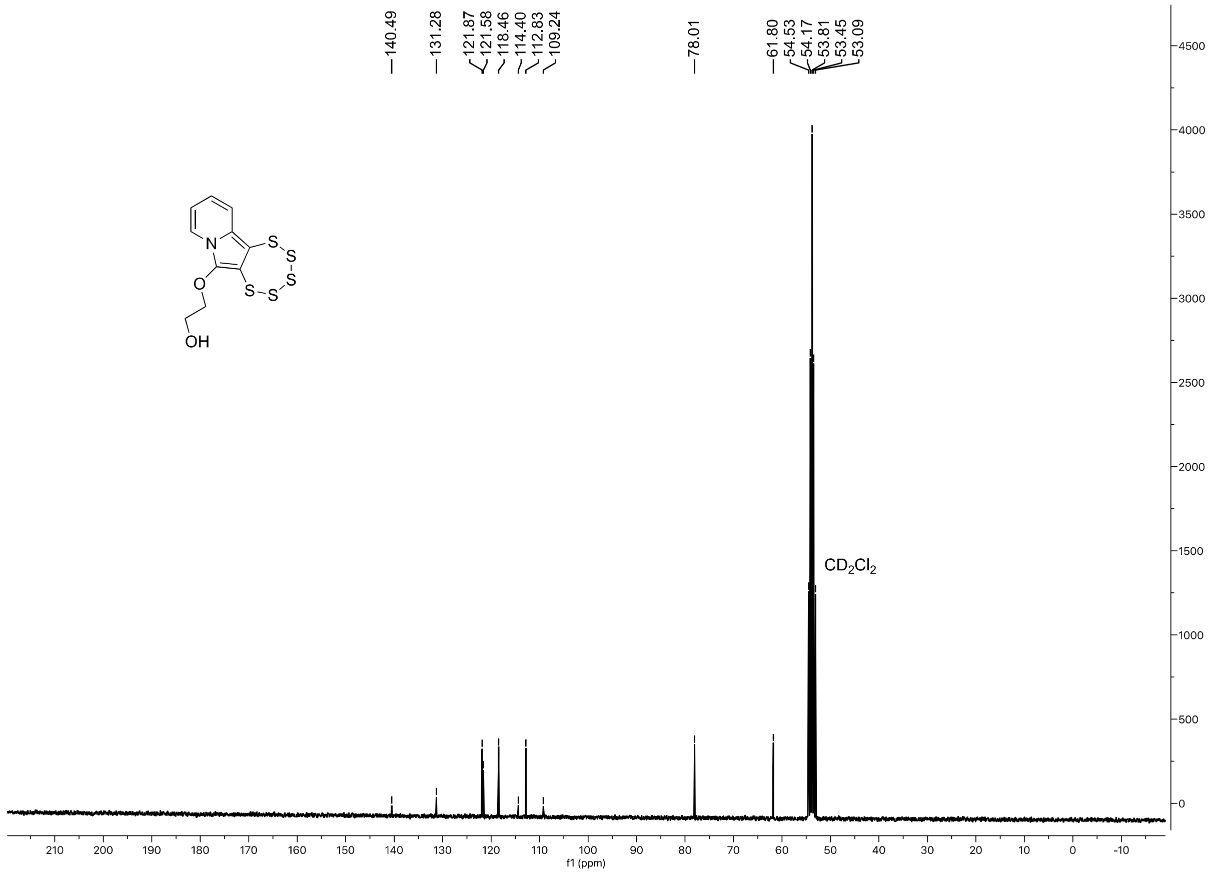

Figure S32. ^{13}C NMR spectrum of 2-([1,2,3,4,5]pentathiepino[6,7-a]indolizin-6-yloxy)ethan-1-ol (**5e**).

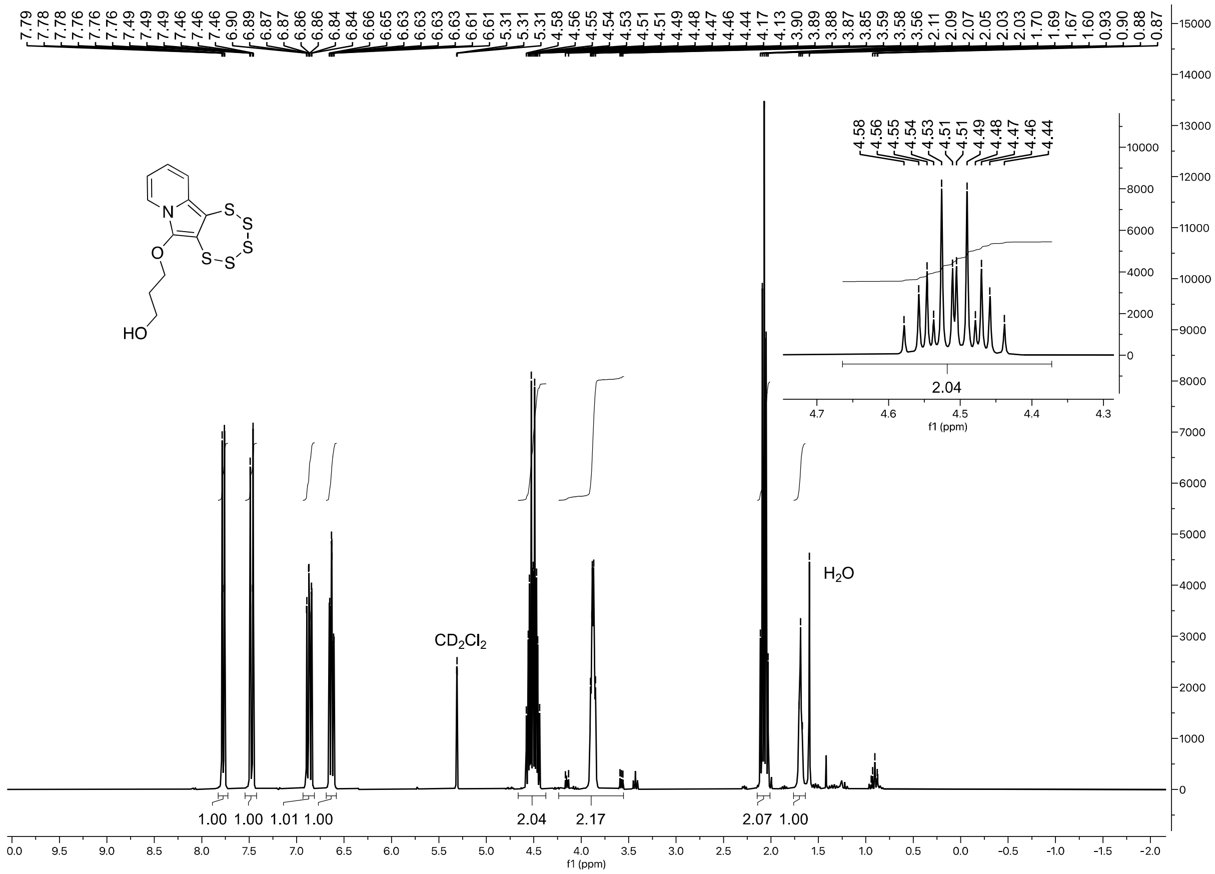

Figure S33. ^{1}H NMR spectrum of 3-([1,2,3,4,5]pentathiepino[6,7-a]indolizin-6-yloxy)propan-1-ol (**5f**).

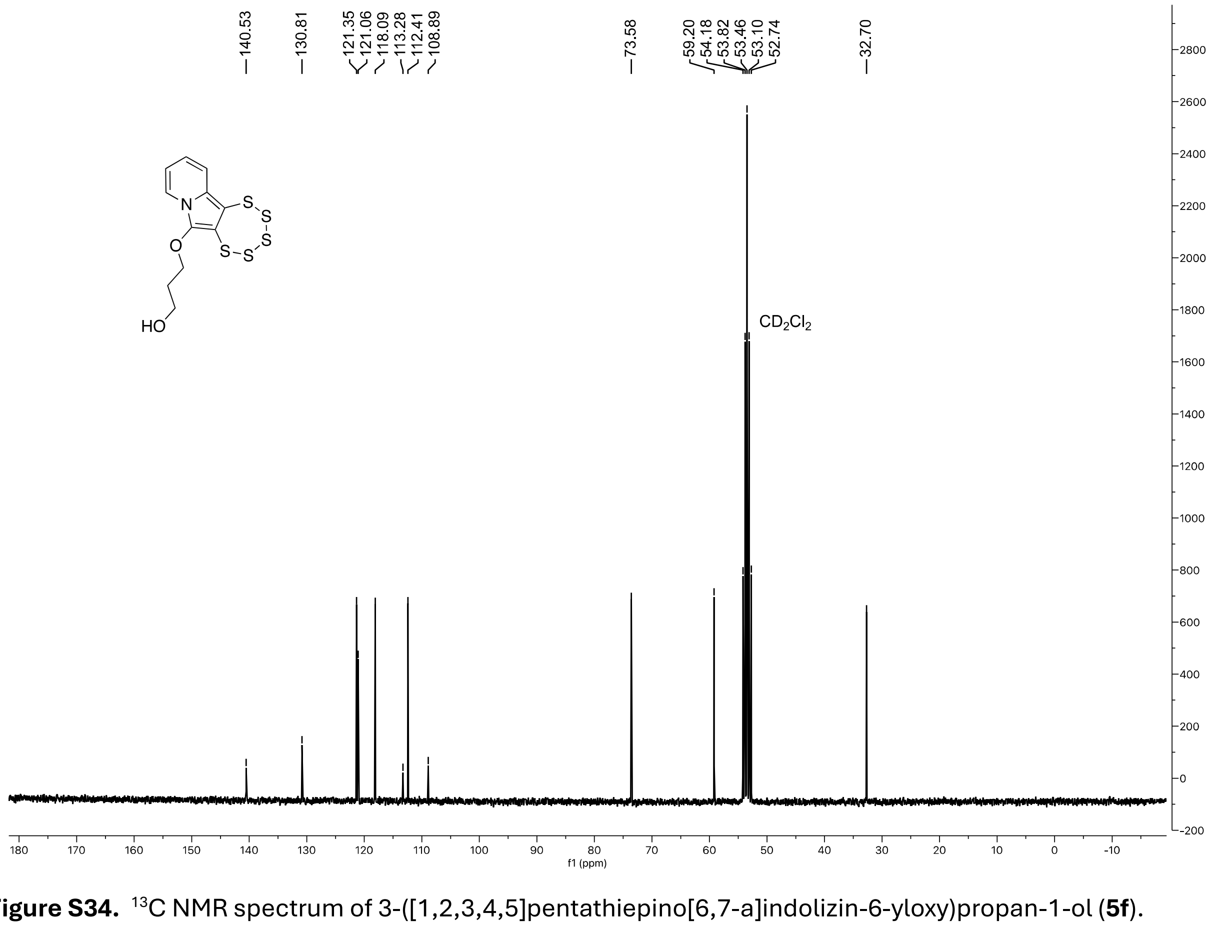

Figure S34. ^{13}C NMR spectrum of 3-([1,2,3,4,5]pentathiepino[6,7-a]indolizin-6-yloxy)propan-1-ol (**5f**).

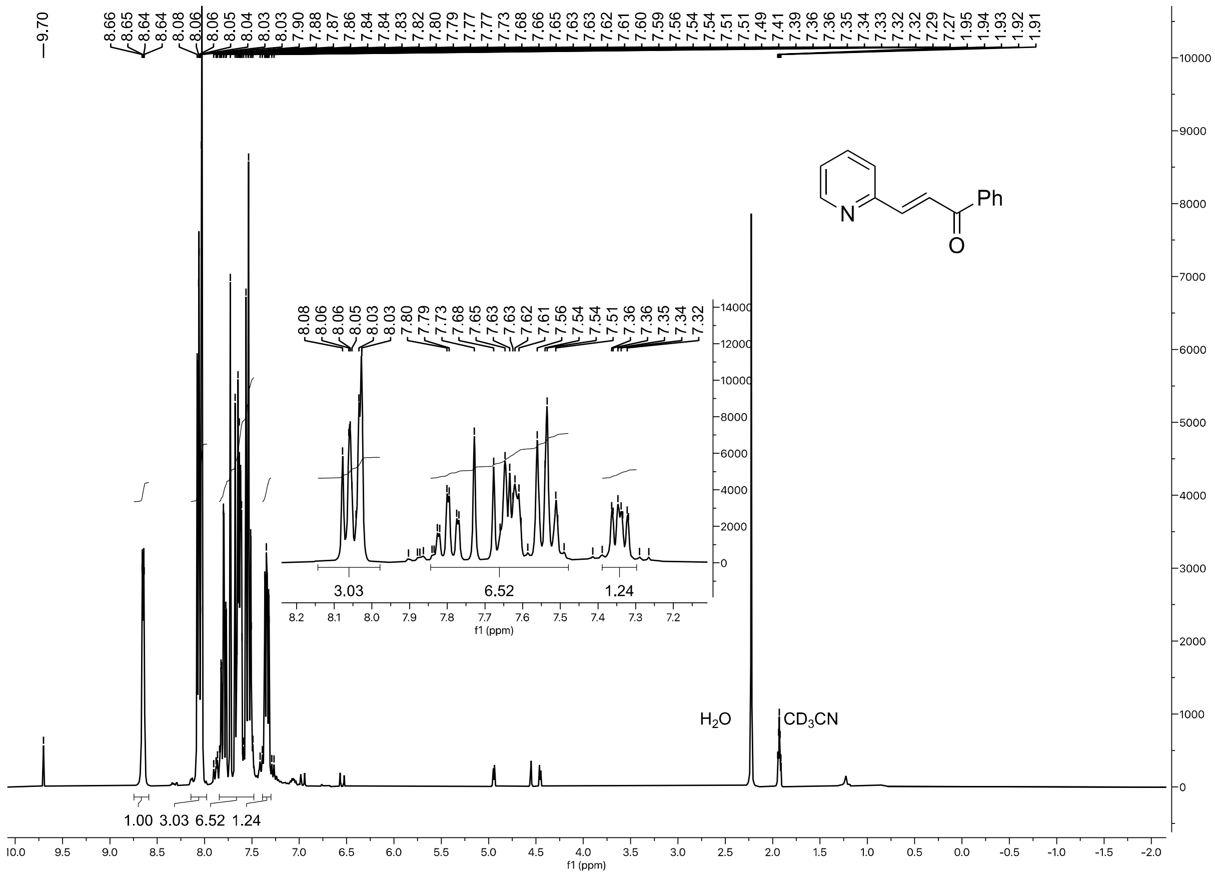

Figure S35. ^{1}H NMR spectrum of (*E*)-1-phenyl-3-(pyridin-2-yl)prop-2-en-1-one (**13**).

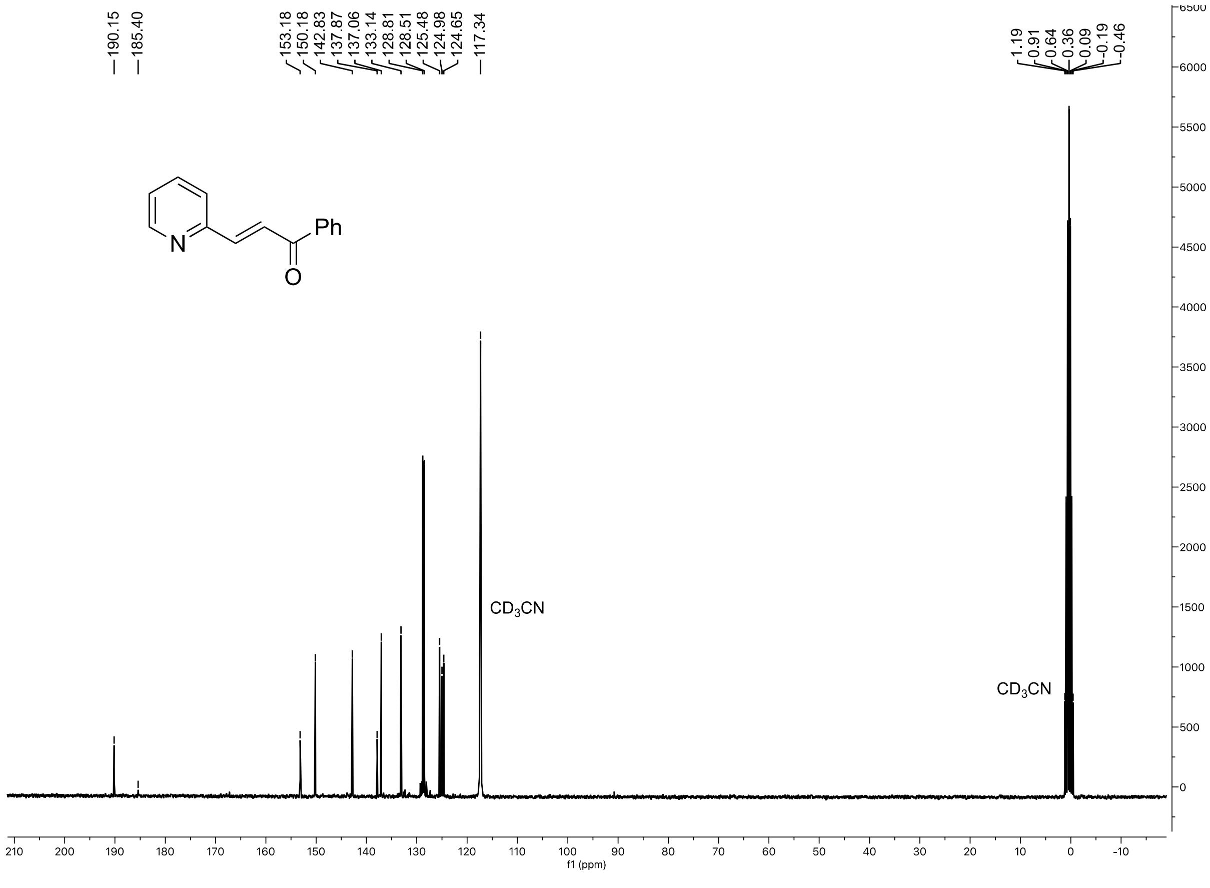

Figure S36. ^{13}C NMR spectrum of (*E*)-1-phenyl-3-(pyridin-2-yl)prop-2-en-1-one (**13**).

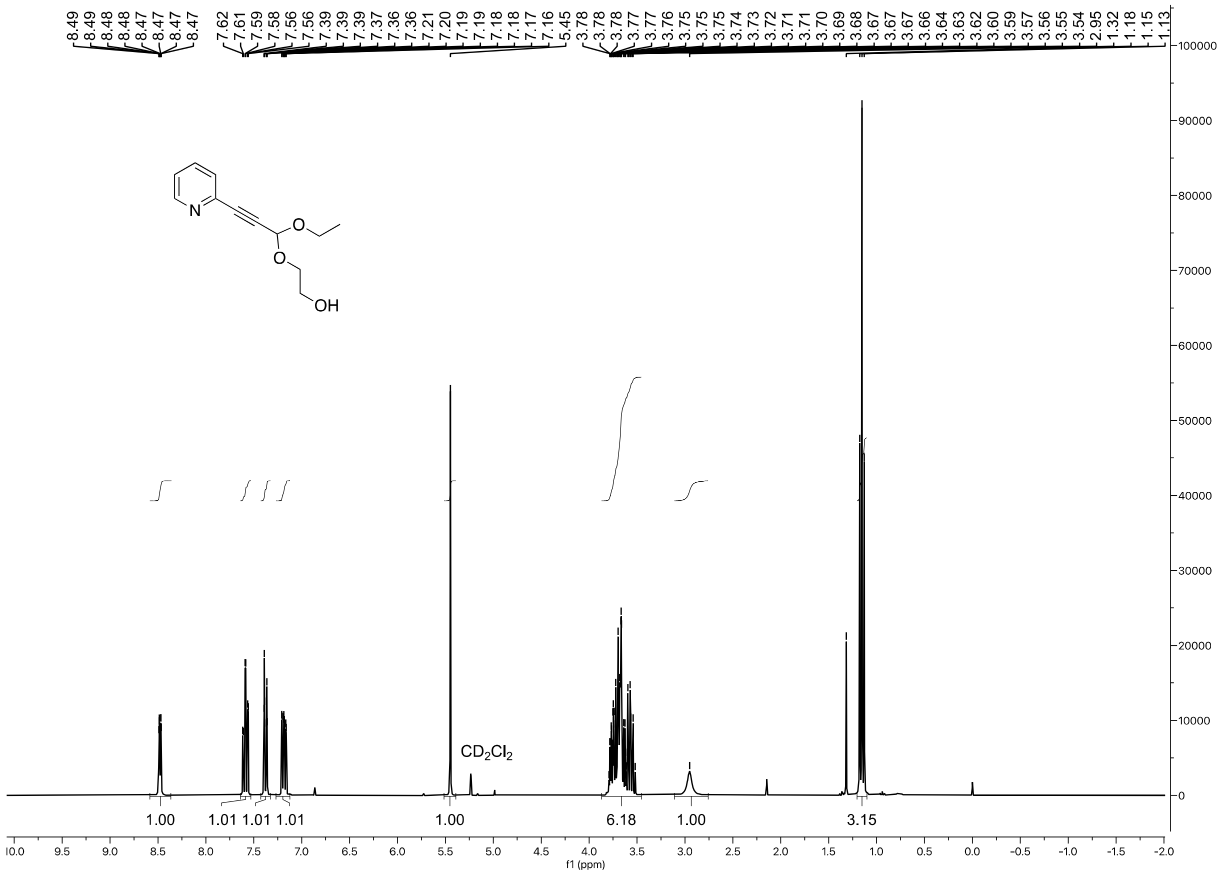

Figure S37. ^{1}H NMR spectrum of 2-((1-ethoxy-3-(pyridin-2-yl)prop-2-yn-1-yl)oxy)ethan-1-ol (**14**).

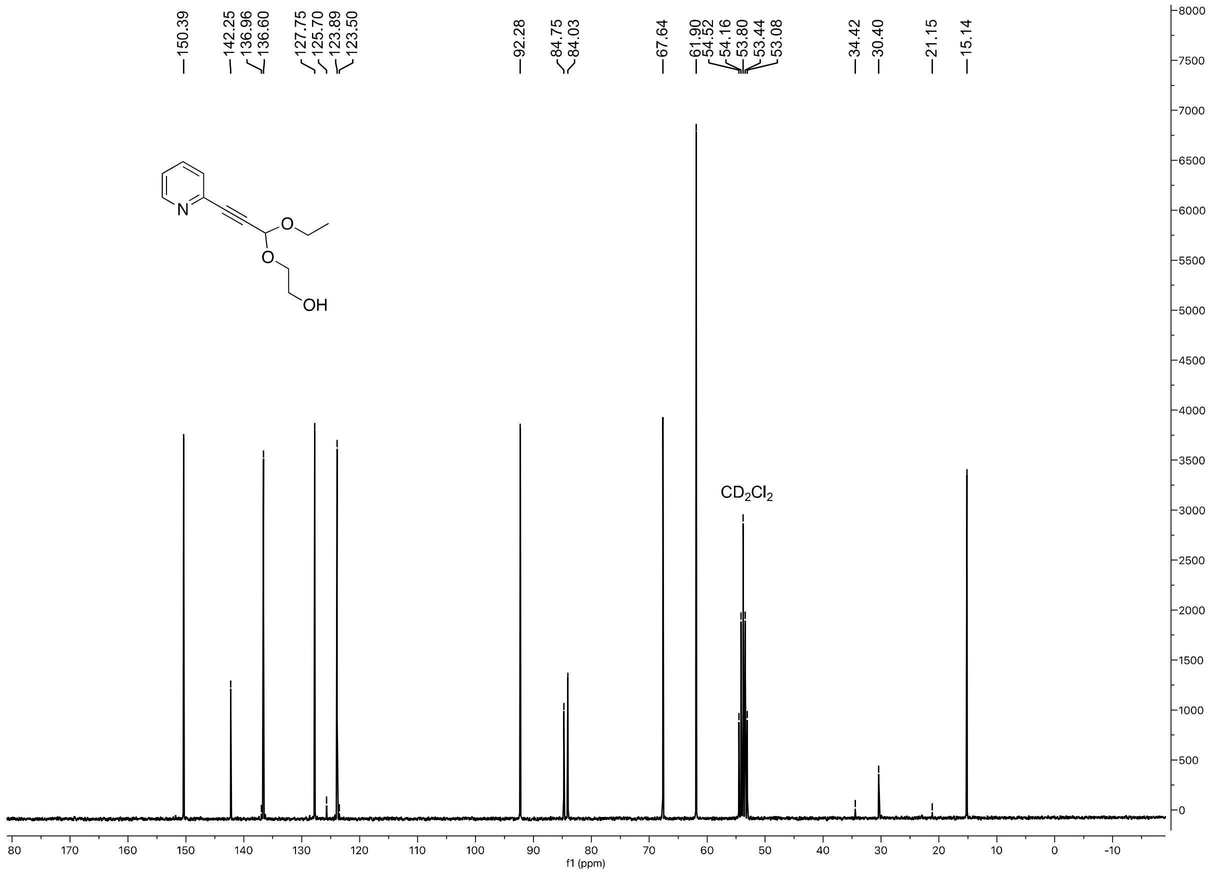

Figure S38. ^{13}C NMR spectrum of 2-((1-ethoxy-3-(pyridin-2-yl)prop-2-yn-1-yl)oxy)ethan-1-ol (**14**).

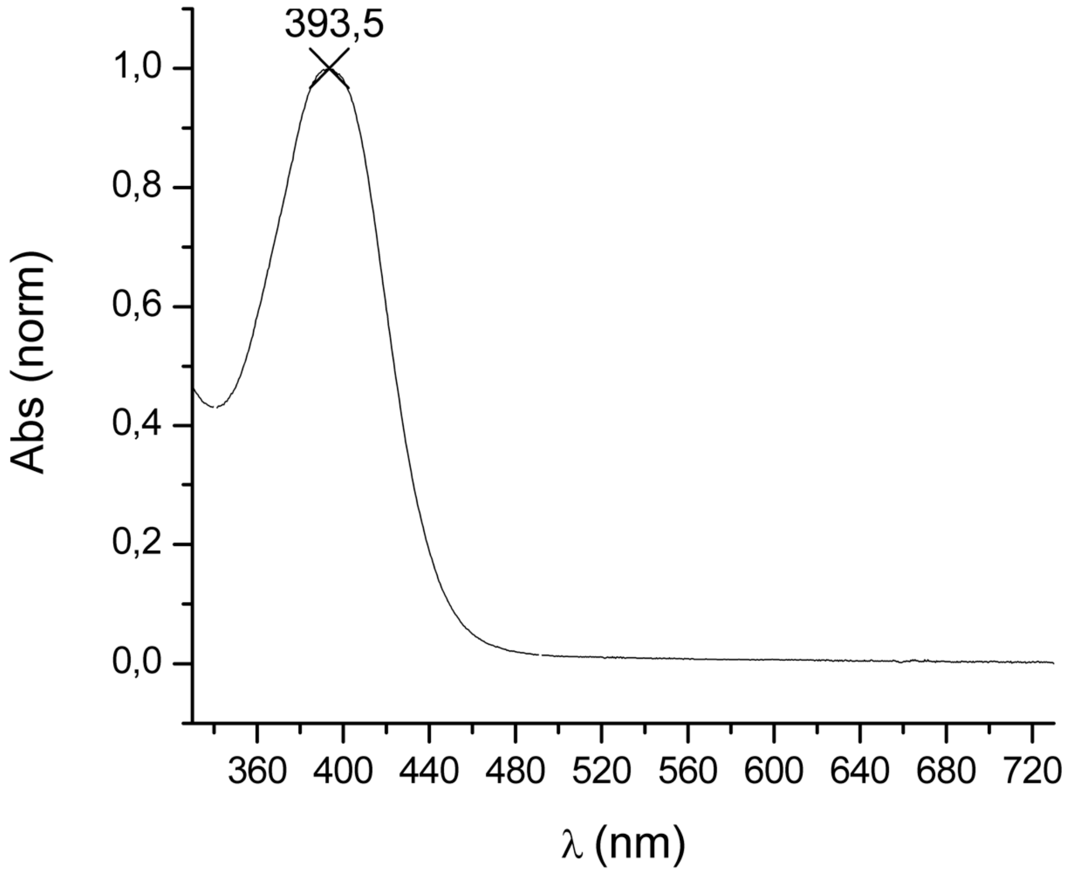

Figure S39. UV-Vis spectrum of 6-methyl-[1,2,3,4,5]pentathiepino[6,7-a]indolizine (**5c**).

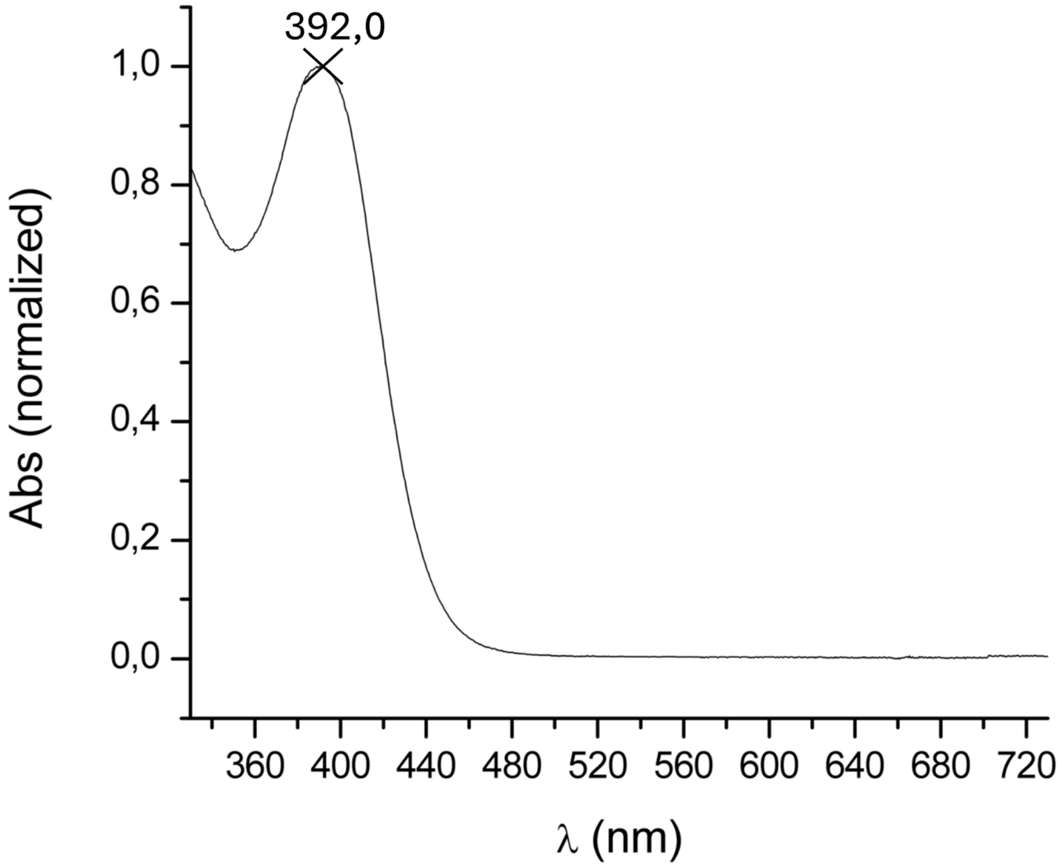

Figure S40. UV-Vis spectrum of 6-phenyl-[1,2,3,4,5]pentathiepino[6,7-a]indolizine (**5d**).

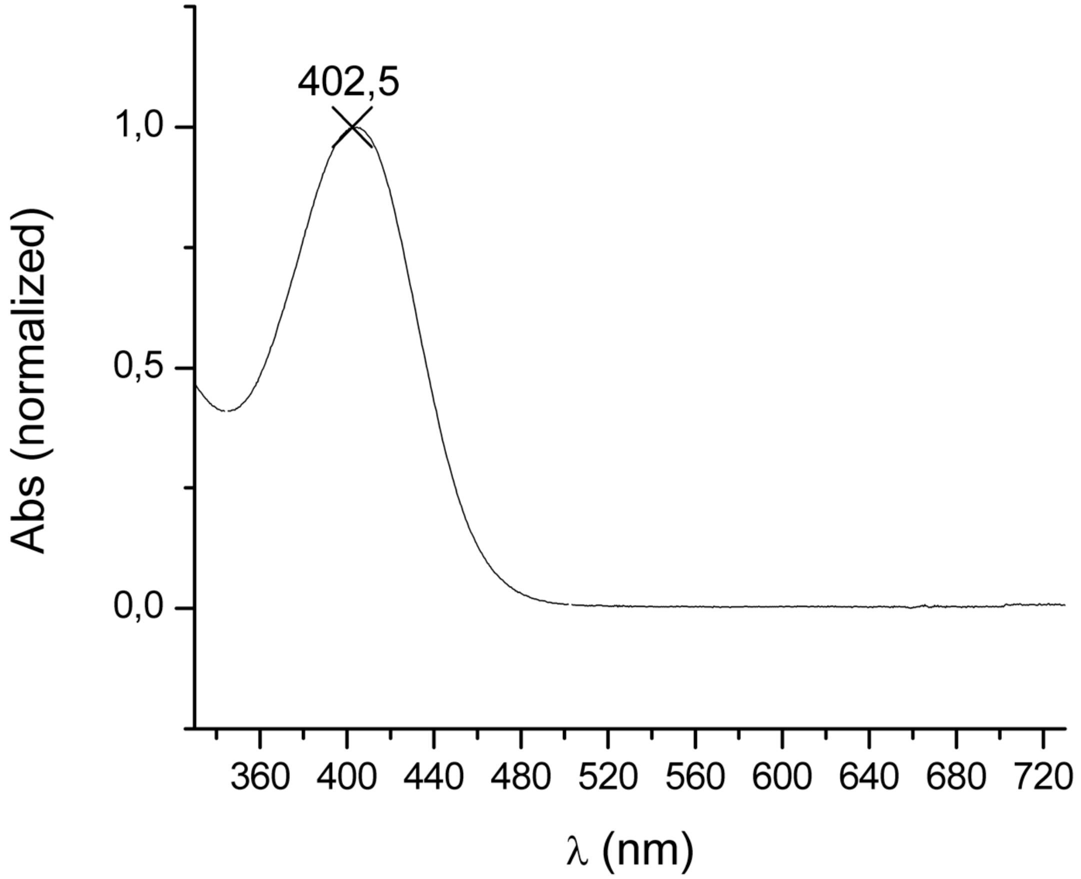

Figure S41. UV-Vis spectrum of 2-([1,2,3,4,5]pentathiepino[6,7-a]indolizin-6-yloxy)ethan-1-ol (**5e**).

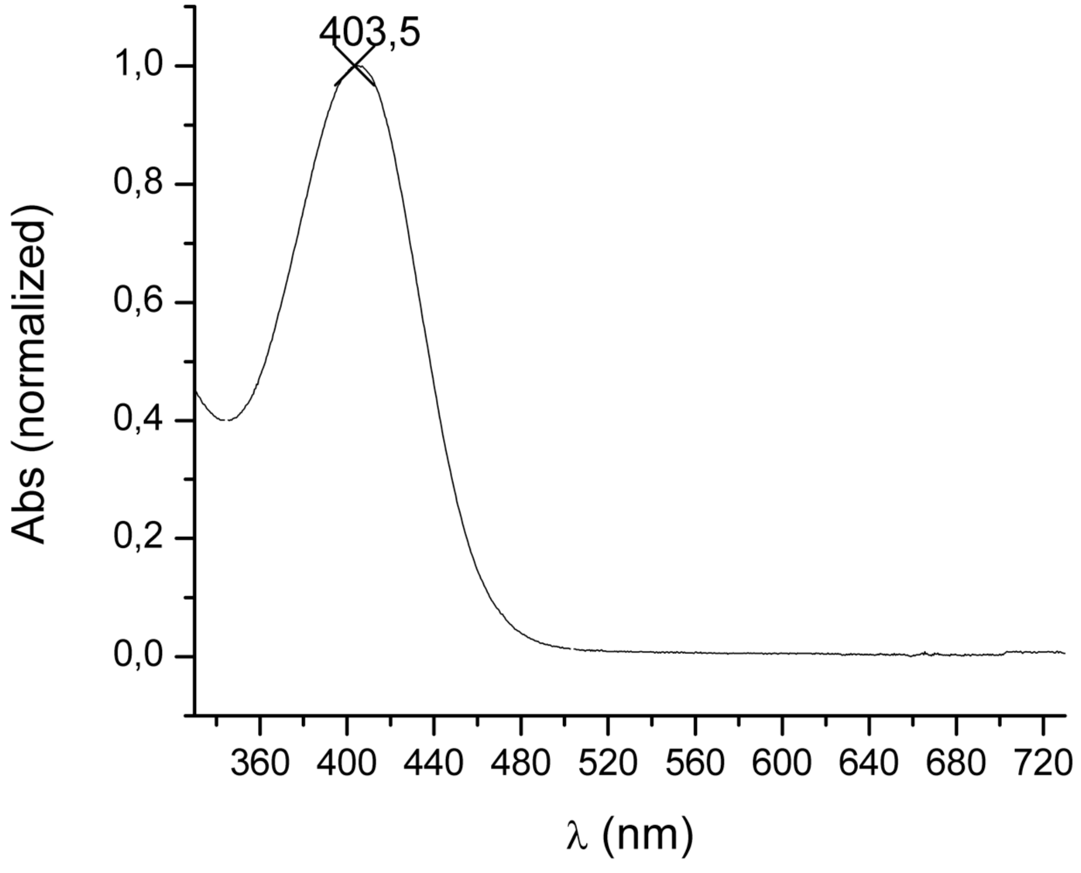

Figure S42. UV-Vis spectrum of 3-([1,2,3,4,5]pentathiepino[6,7-a]indolizin-6-yloxy)propan-1-ol (**5f**).

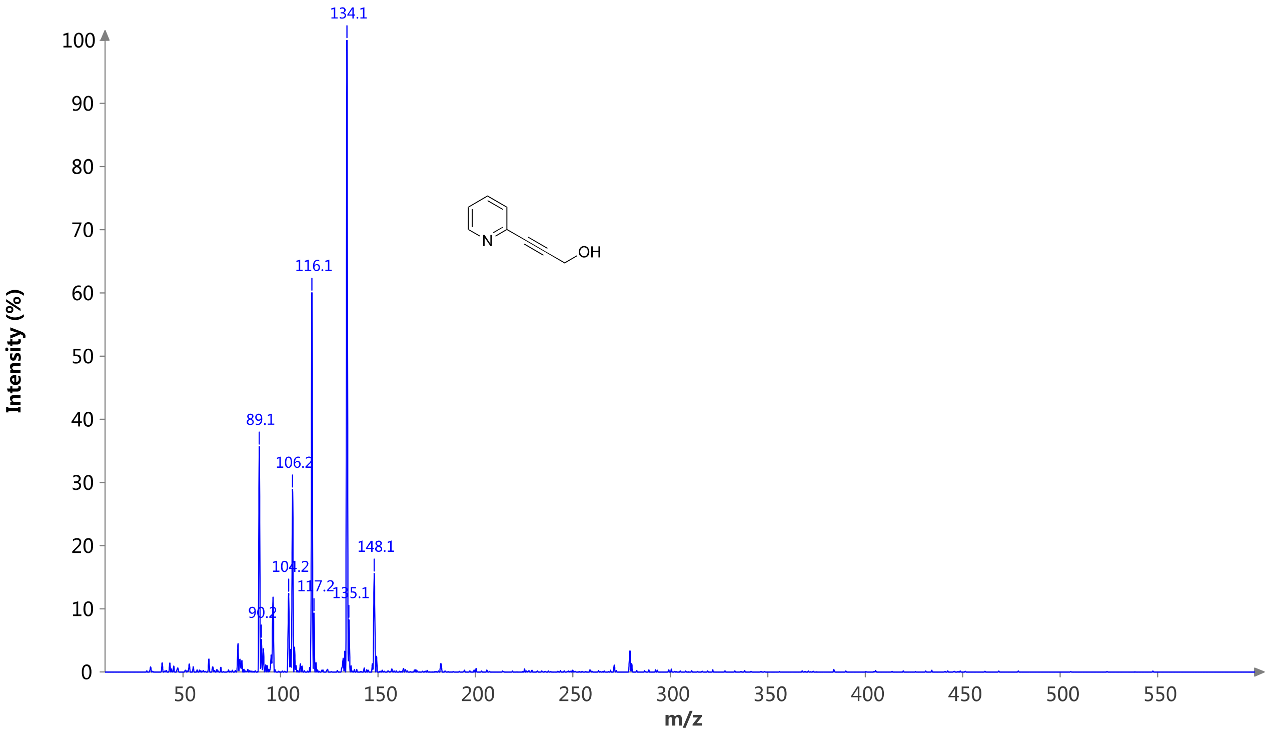

Figure S43. APCI Mass spectrum of of 3-(pyridin-2-yl)prop-2-yn-1-ol (**3a**).

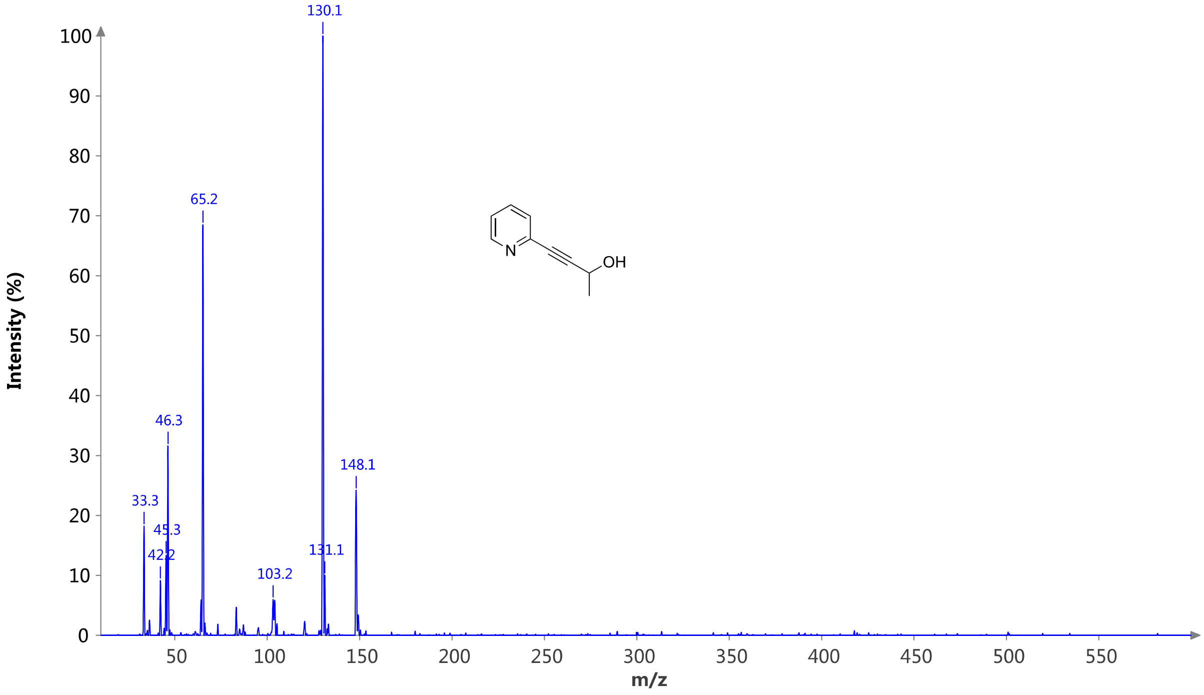

Figure S44. APCI Mass spectrum of 4-(pyridin-2-yl)but-3-yn-2-ol (**3c**).

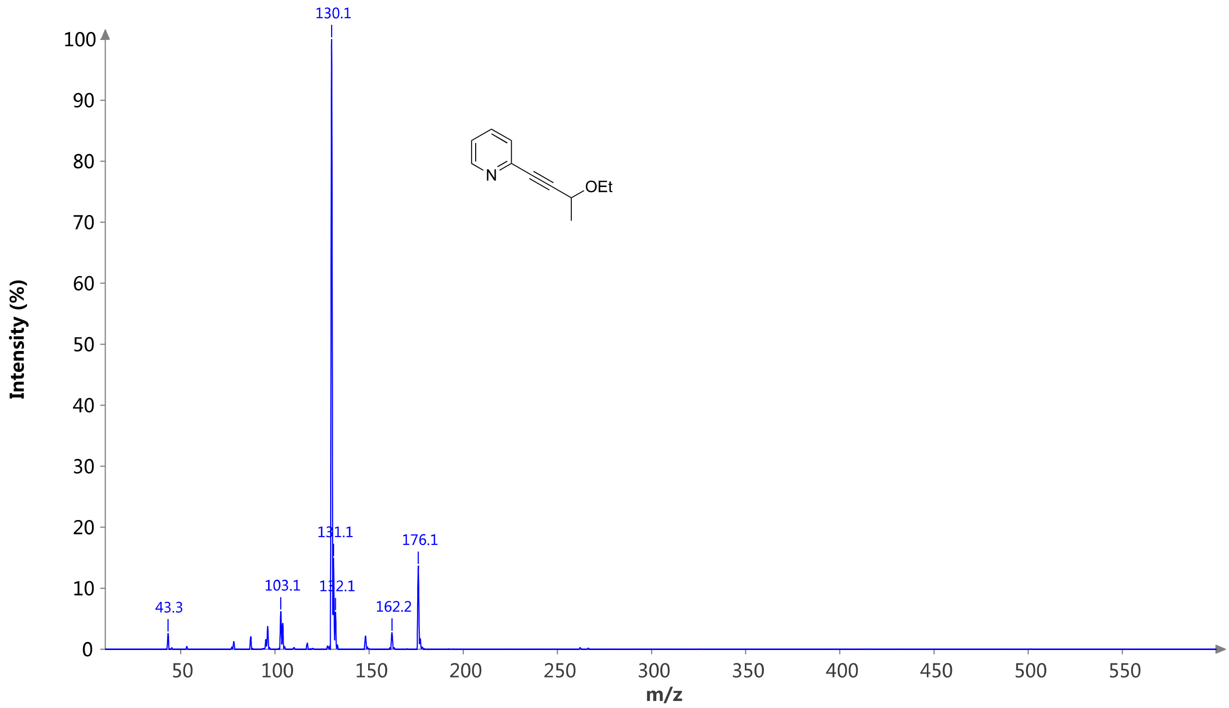

Figure S45. APCI Mass spectrum of 2-(3-ethoxybut-1-yn-1-yl)pyridine (**4c**).

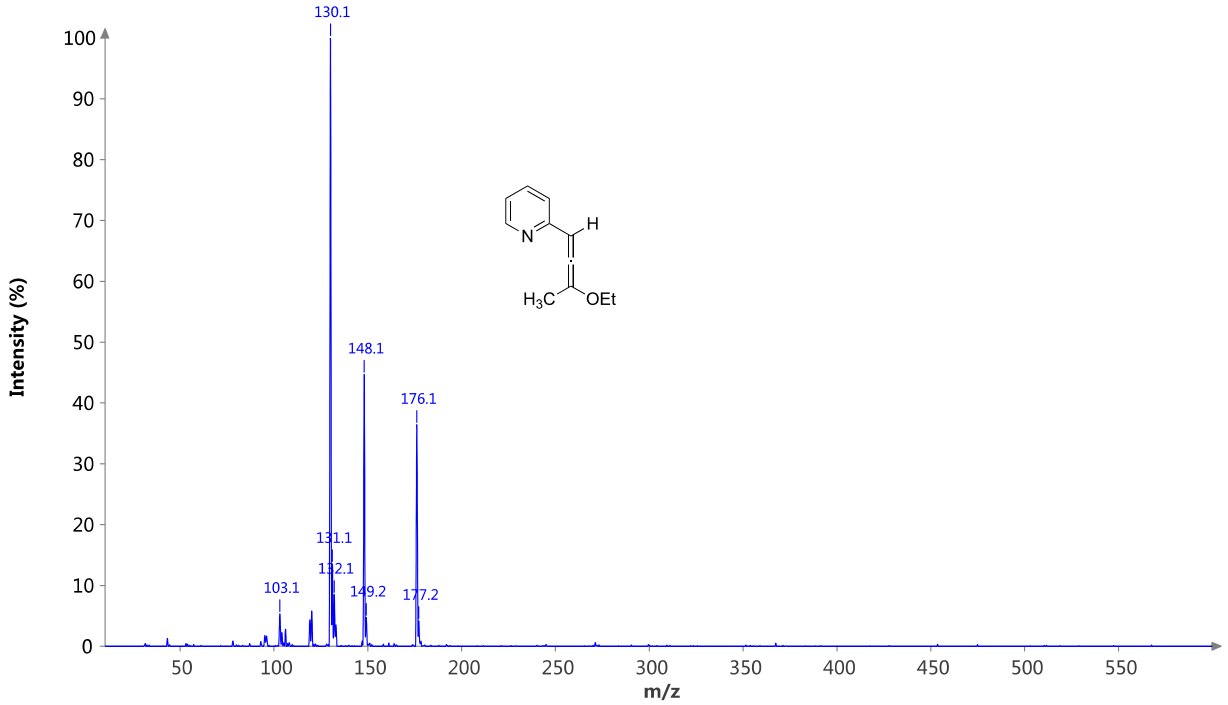

Figure S46. APCI Mass spectrum of 2-(3-ethoxybuta-1,2-dien-1-yl)pyridine (**4g**).

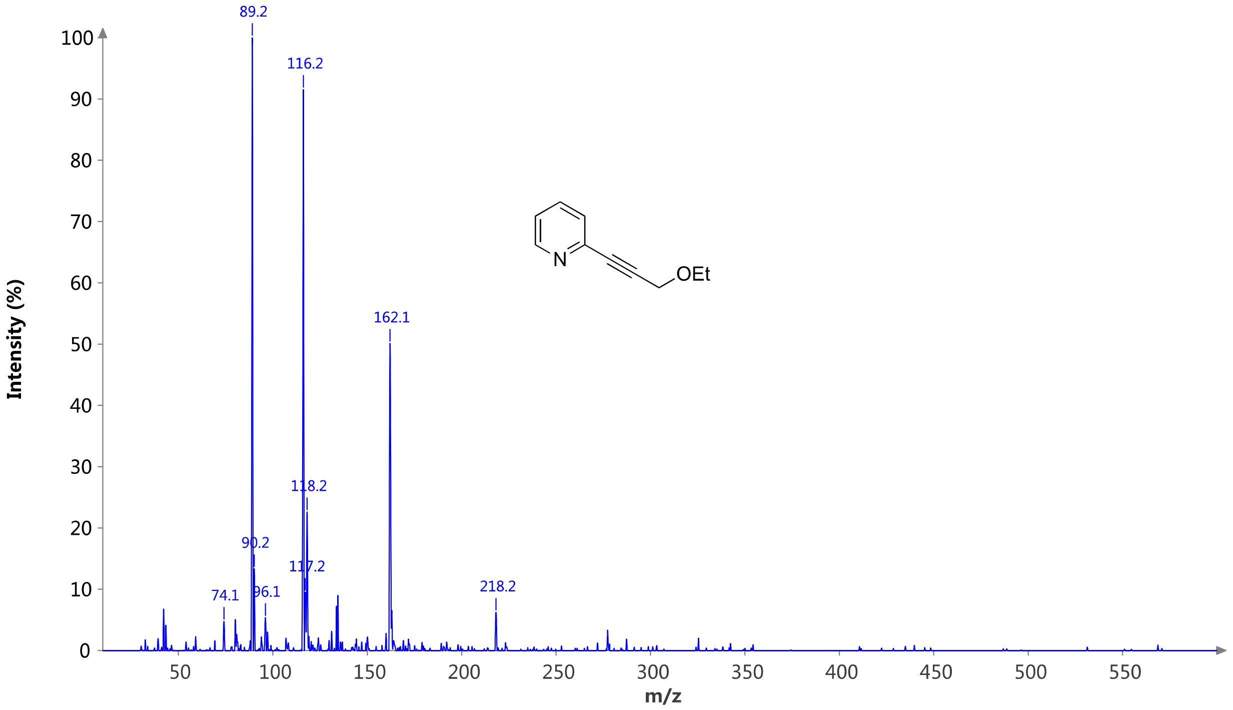

Figure S47. APCI Mass spectrum of 2-(3-ethoxyprop-1-yn-1-yl)pyridine (**4b**).

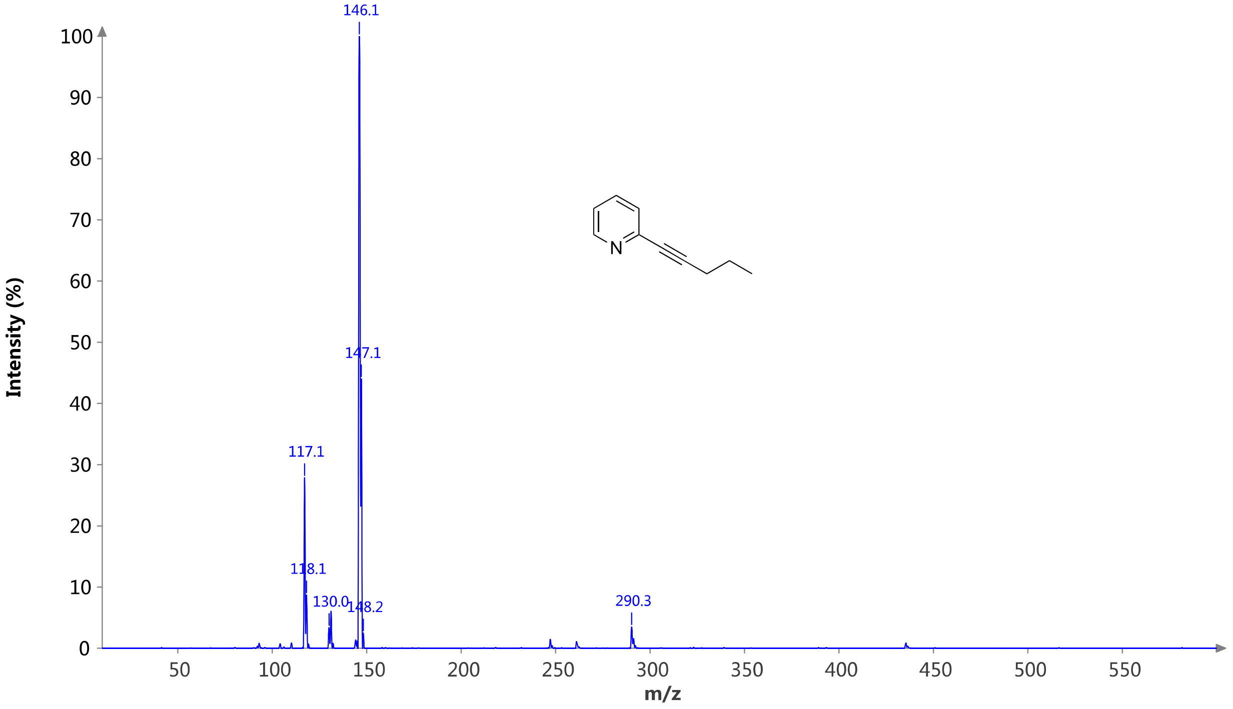

Figure S48. APCI Mass spectrum of 2-(pent-1-yn-1-yl)pyridine (**4'a**).

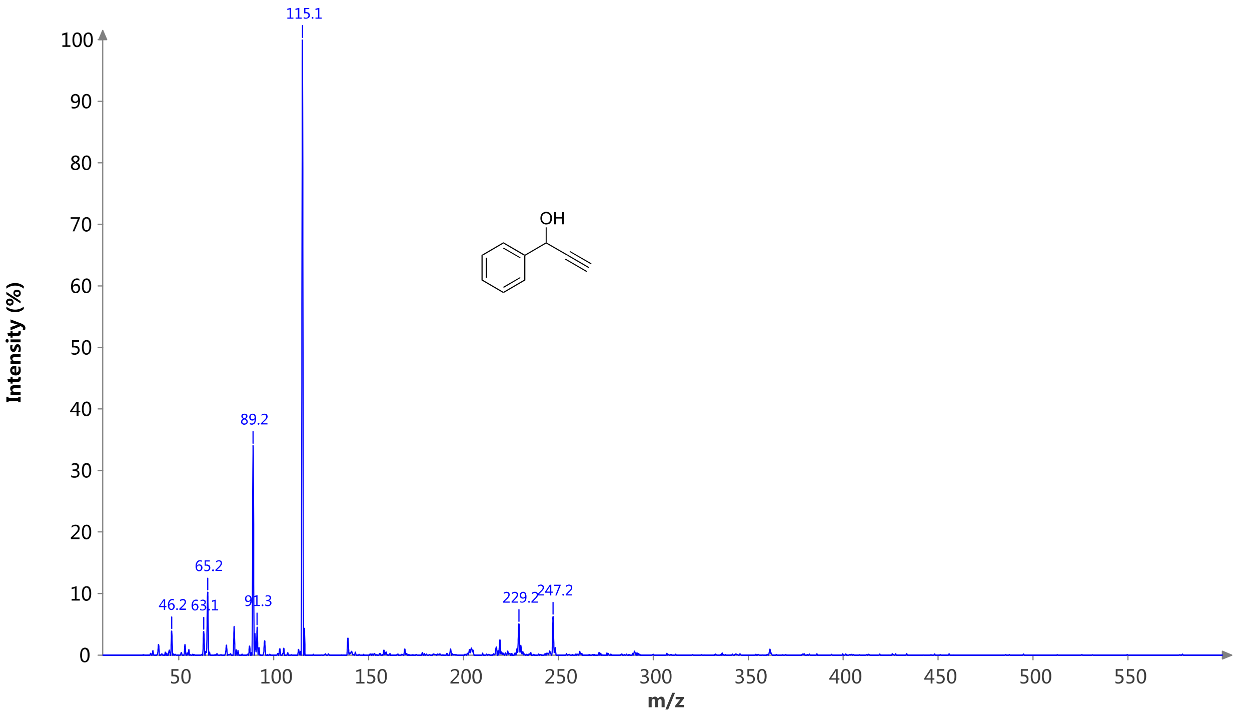

Figure S49. APCI Mass spectrum of 1-phenylprop-2-yn-1-ol (**2d**).

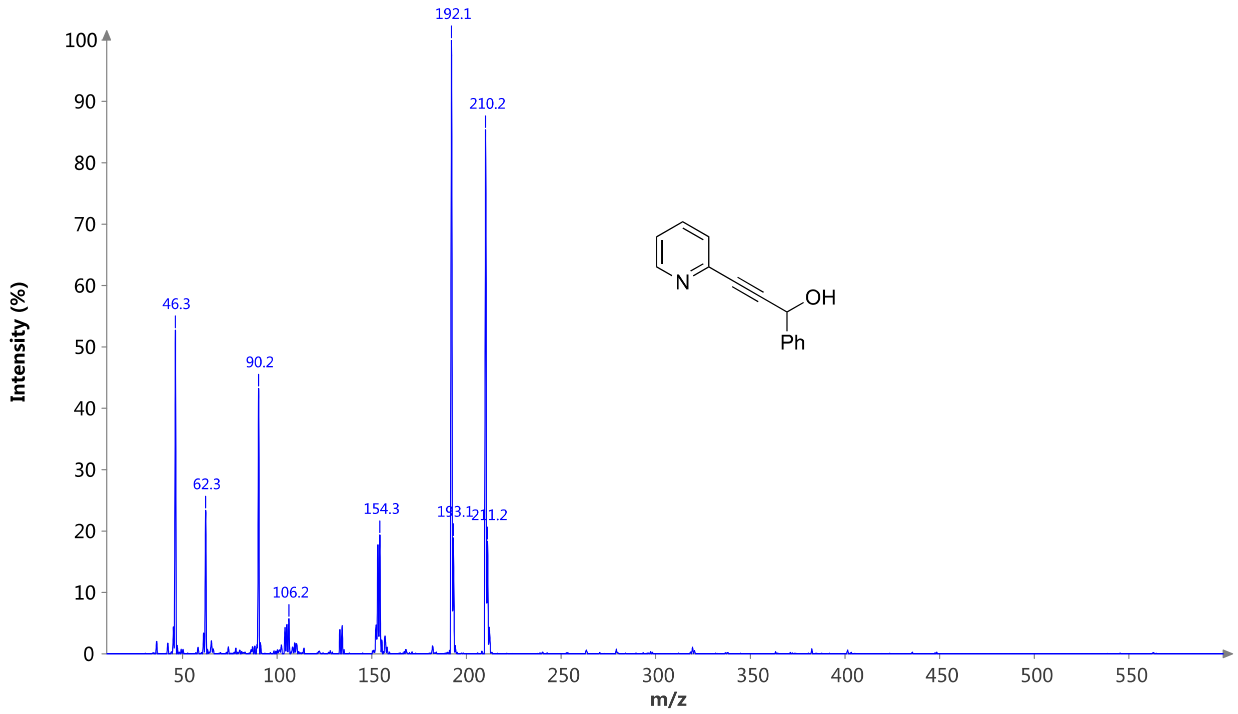

Figure S50. APCI Mass spectrum of 1-phenyl-3-(pyridin-2-yl)prop-2-yn-1-ol (**3d**).

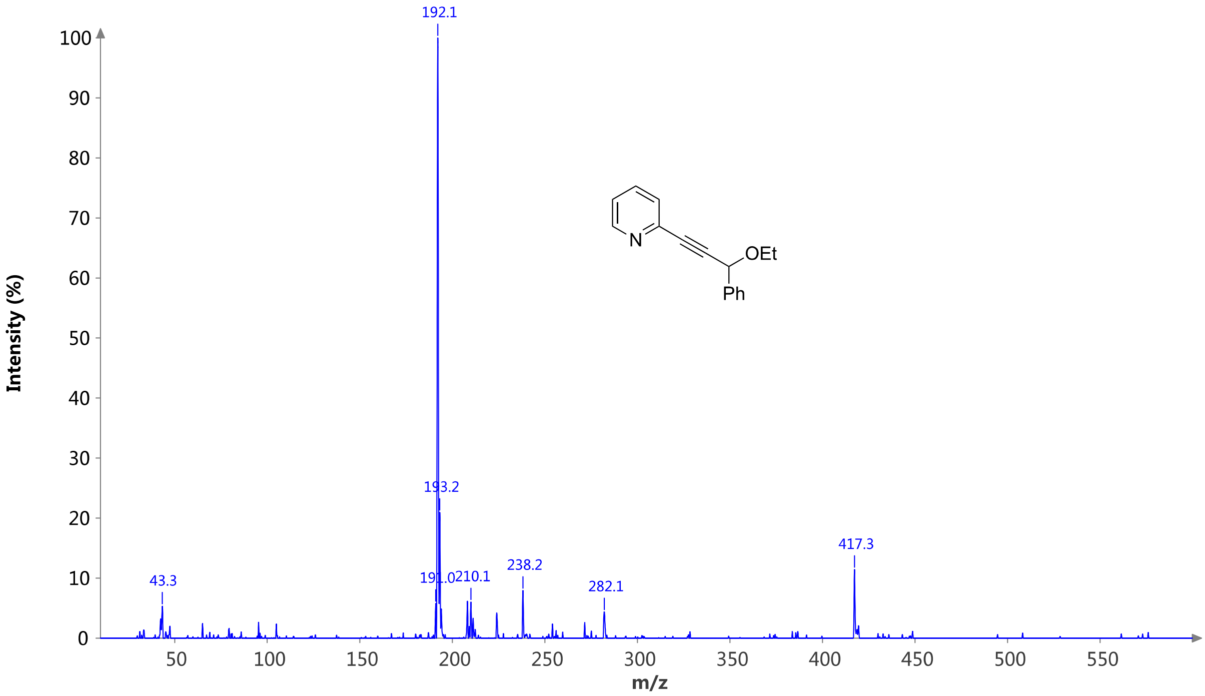

Figure S51. APCI Mass spectrum of 2-(3-ethoxy-3-phenylprop-1-yn-1-yl)pyridine (**4d**).

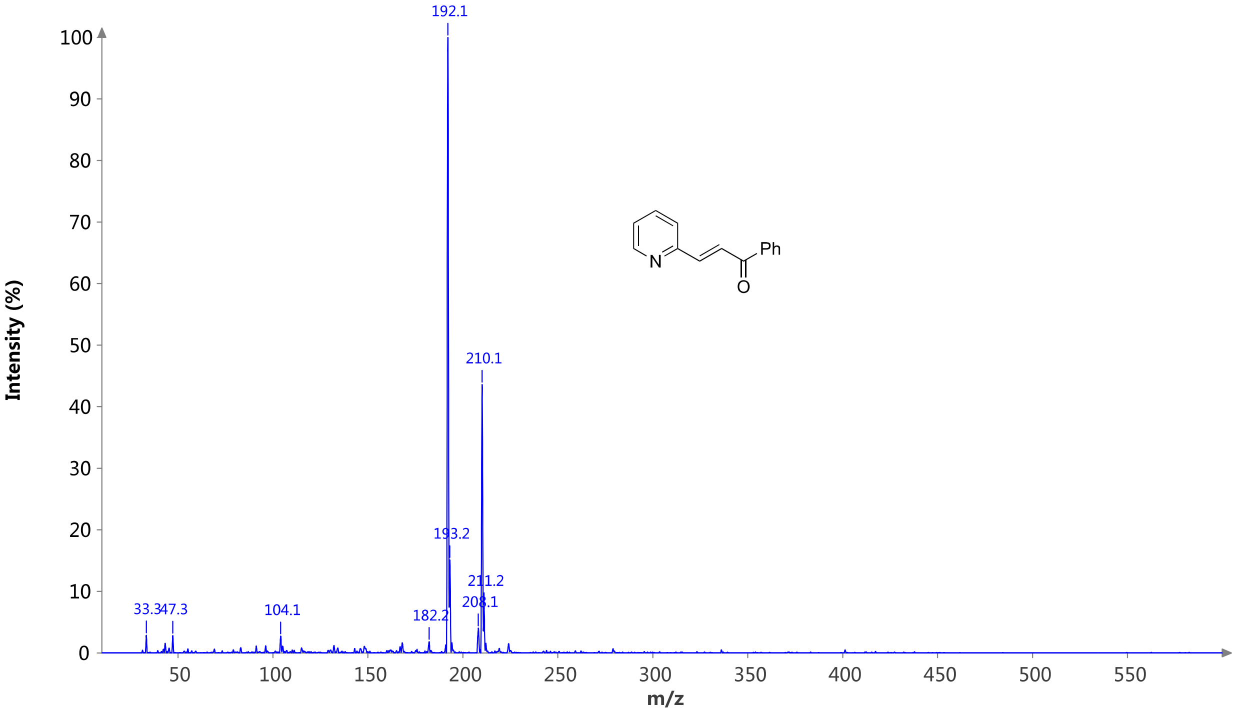

Figure S52. APCI Mass spectrum of (*E*)-1-phenyl-3-(pyridin-2-yl)prop-2-en-1-one (**13**).

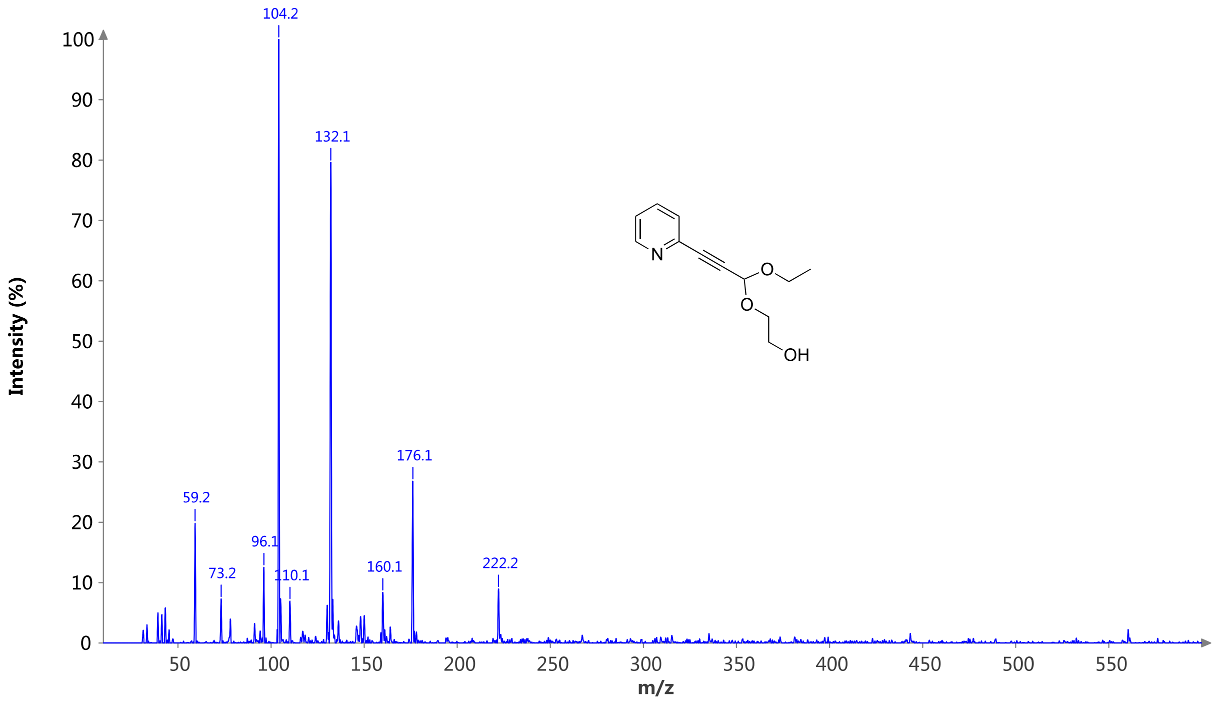

Figure S53. APCI Mass spectrum of 2-((1-ethoxy-3-(pyridin-2-yl)prop-2-yn-1-yl)oxy)ethan-1-ol (**14**).

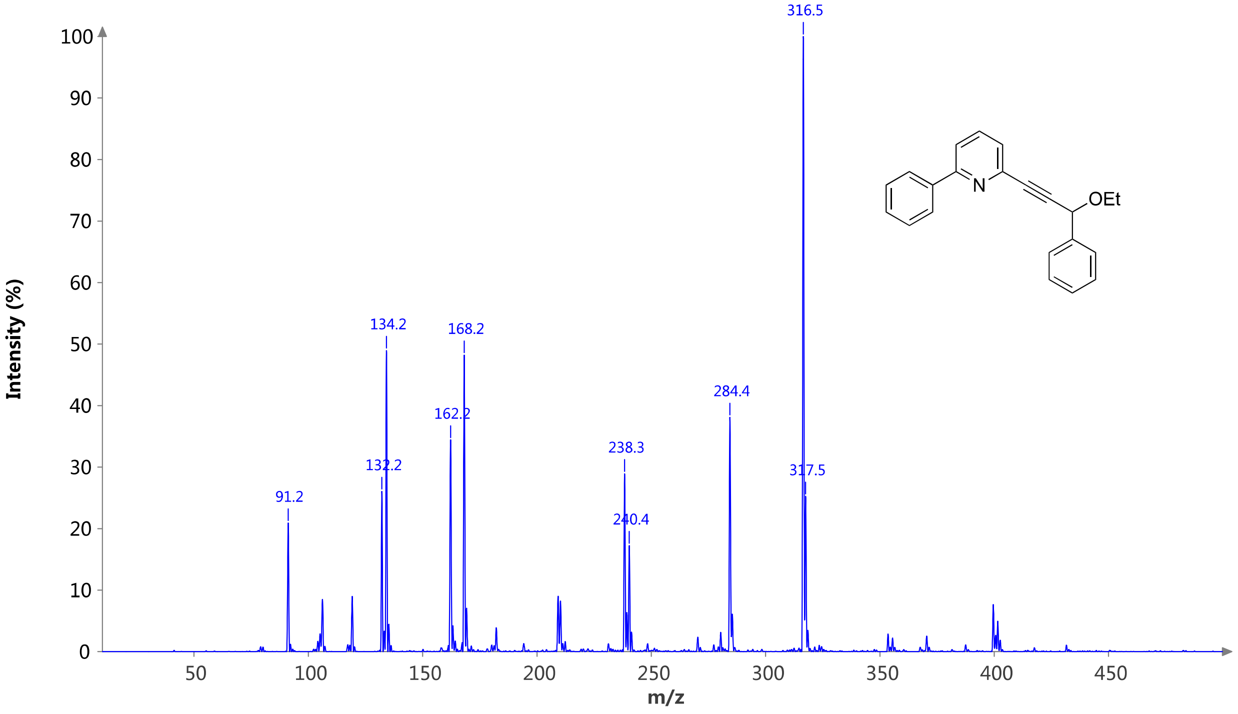

Figure S54. APCI Mass spectrum of 2-(3-ethoxy-3-phenylprop-1-yn-1-yl)-6-phenylpyridine (**8**).

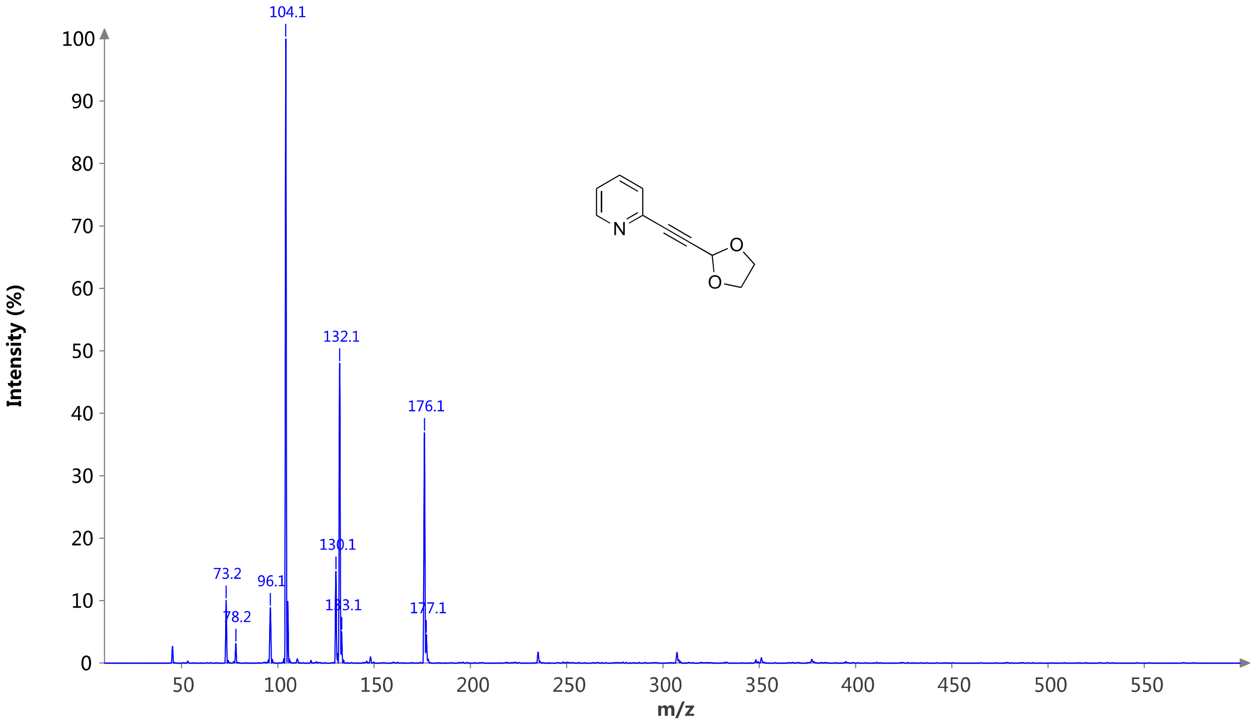

Figure S55. APCI Mass spectrum of 2-((1,3-dioxolan-2-yl)ethynyl)pyridine (**4e**).

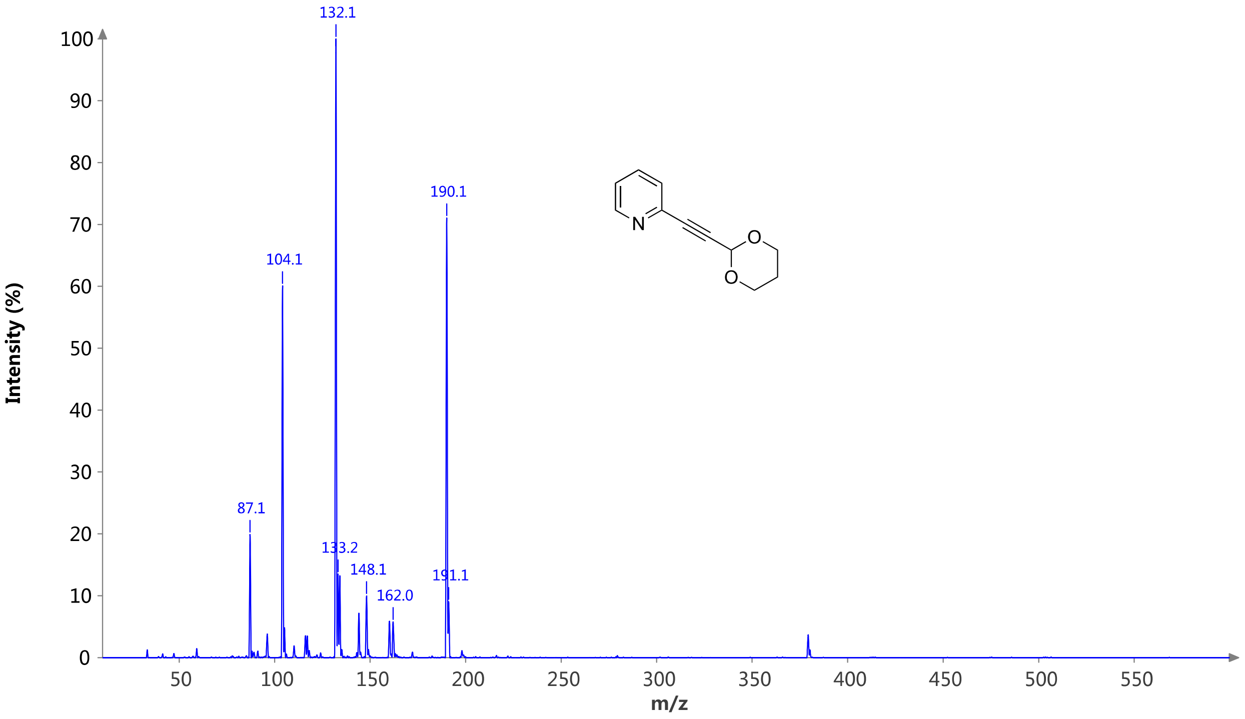

Figure S56. APCI Mass spectrum of 2-((1,3-dioxan-2-yl)ethynyl)pyridine (**4f**).

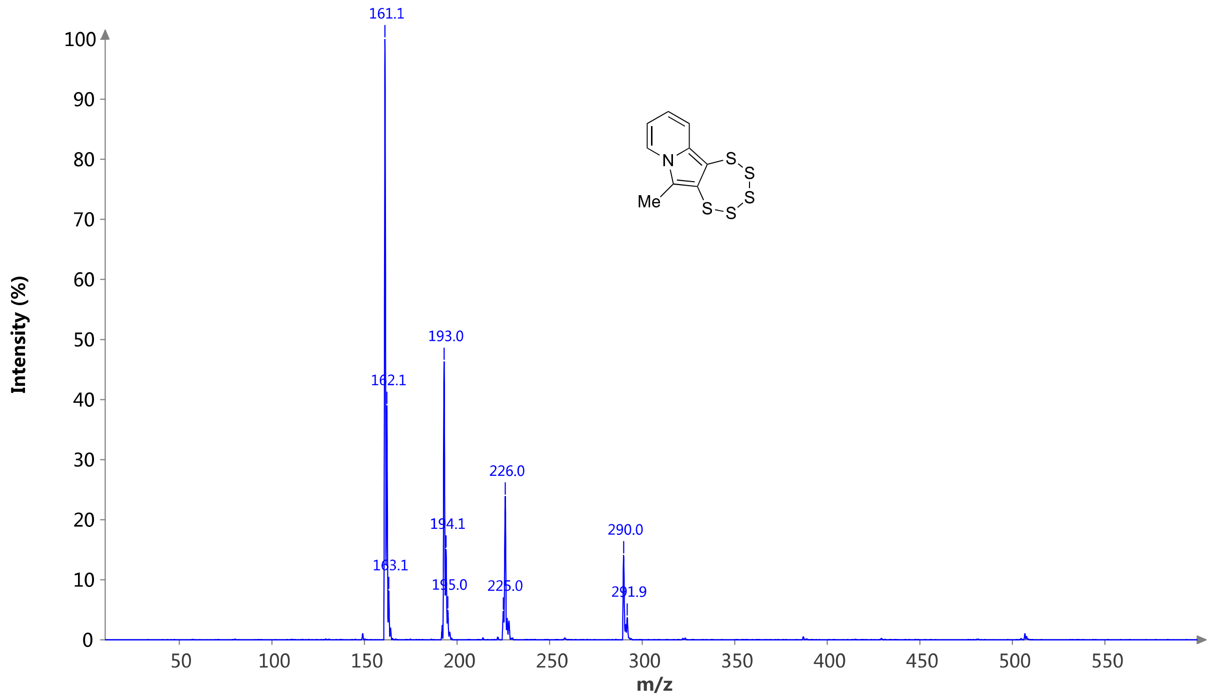

Figure S57. APCI Mass spectrum of 6-methyl-[1,2,3,4,5]pentathiepino[6,7-*a*]indolizine (**5c**).

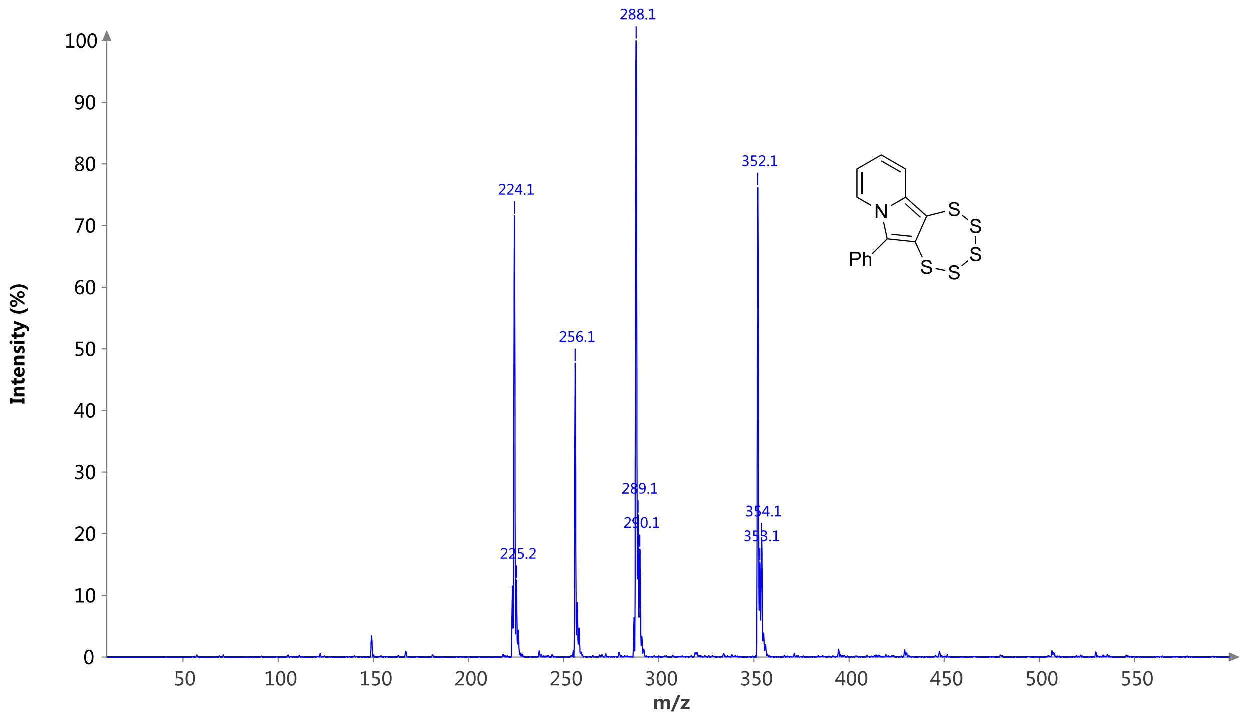

Figure S58. APCI Mass spectrum of 6-phenyl-[1,2,3,4,5]pentathiepino[6,7-*a*]indolizine (**5d**).

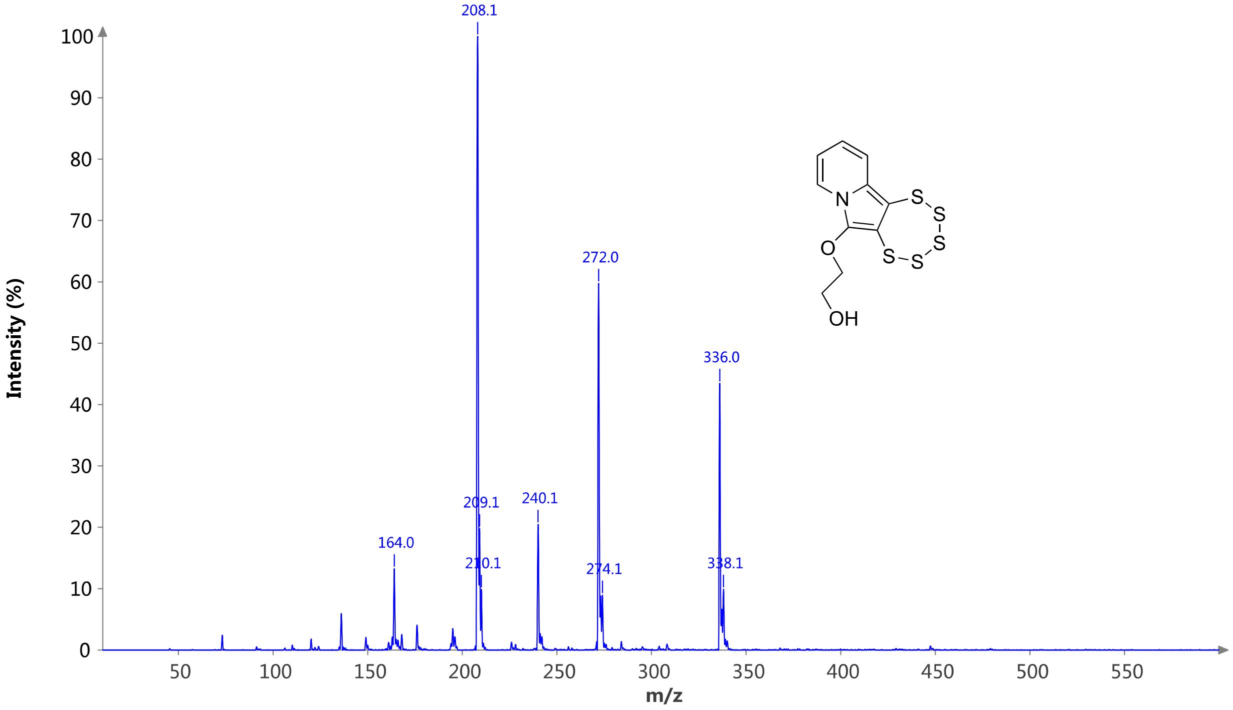

Figure S59. APCI Mass spectrum of 2-([1,2,3,4,5]pentathiepino[6,7-*a*]indolizin-6-yloxy)ethan-1-ol (**5e**).

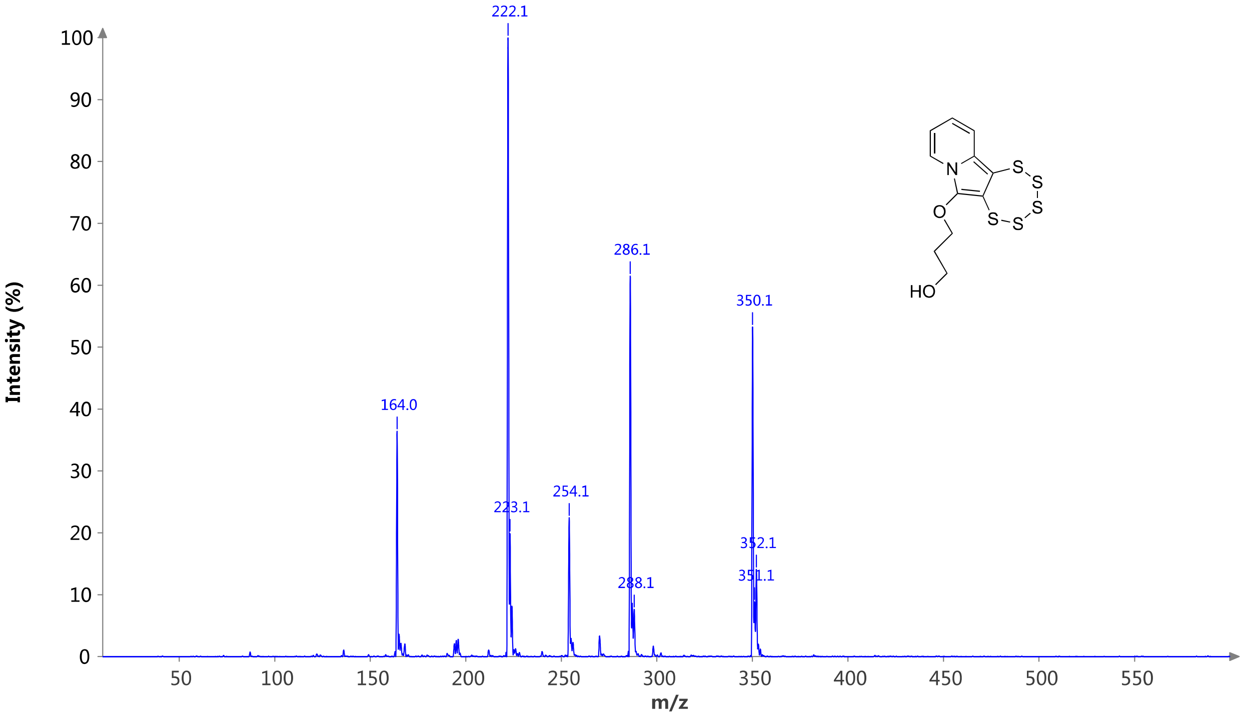

Figure S60. APCI Mass spectrum of 3-([1,2,3,4,5]pentathiepino[6,7-*a*]indolizin-6-yloxy)propan-1-ol (**5f**).

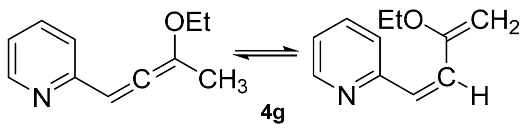

4g

Figure S61. Tautomerism of precursor **4g** (2-(3-ethoxybuta-1,2-dien-1-yl)pyridine to the left and 2-(3-ethoxybuta-1,3-dien-1-yl)pyridine to the right).

The NMR data of precursor 4g shows that its tautomeric form, namely 2-(3-ethoxybuta-1,3-dien-1-yl)pyridine (Figure S61, right), is the predominant species in solution.

Table S1-1. Crystal data and structure refinement for 3-(pyridine-2-yl)prop-2-yn-1-ol **3a**.

Identification code	RT22
Empirical formula	C8 H7 N O
Formula weight	133.15
Temperature	297(2) K
Wavelength	1.54184 A
Crystal system, space group	Triclinic, P -1
Unit cell dimensions	a = 4.07140(10) A alpha = 100.064(2) deg.
	b = 8.8863(2) A beta = 92.379(2) deg.
	c = 9.7115(2) A gamma = 98.795(2) deg.
Volume	341.043(14) A^3
Z, Calculated density	2, 1.297 Mg/m^3
Absorption coefficient	0.704 mm^-1
F(000)	140
Crystal Size	0.190 x 0.140 x 0.090 mm
Theta range for data collection	4.636 to 79.850 deg.
Limiting indices	-5<=h<=5, -10<=k<=11, -12<=l<=11
Reflections collected / unique	7707 / 1449 [R(int) = 0.0842]
Completeness to theta = 0.770	0.0 %
Absorption correction	Numerical
Max. and min. transmission	1.000 and 0.632
Refinement method	Full-matrix least-squares on F^2
Data / restraints / parameters	1449 / 0 / 103
Goodness-of-fit on F^2	1.105
Final R indices [I>2sigma(I)]	R1 = 0.0463, wR2 = 0.1322
R indices (all data)	R1 = 0.0496, wR2 = 0.1362
Extinction coefficient	n/a
Largest diff. peak and hole	0.128 and -0.219 e.A^-3

Table S1-2. Atomic coordinates (x 10^4) and equivalent isotropic

displacement parameters (A^2 x 10^3) for rt22.
U(eq) is defined as one third of the trace of the orthogonalized
Uij tensor.

	x	y	z	U(eq)
N(1)	4334(3)	1508(1)	2007(1)	54(1)
O(1)	7356(2)	1640(1)	7771(1)	53(1)
C(1)	3001(4)	1866(2)	850(1)	62(1)
C(2)	2567(3)	3341(2)	717(1)	60(1)
C(3)	3644(3)	4533(2)	1824(1)	58(1)
C(4)	5098(3)	4204(1)	3023(1)	53(1)
C(5)	5368(3)	2678(1)	3090(1)	45(1)
C(6)	6764(3)	2284(1)	4339(1)	49(1)
C(7)	7902(3)	1966(1)	5385(1)	49(1)
C(8)	9413(3)	1570(2)	6645(1)	54(1)

Table S1-3. Bond lengths [A] and angles [deg] for rt22.

N(1)-C(1)	1.3359(17)
N(1)-C(5)	1.3462(15)
O(1)-C(8)	1.4034(15)
C(1)-C(2)	1.377(2)
C(2)-C(3)	1.375(2)
C(3)-C(4)	1.3799(18)
C(4)-C(5)	1.3902(17)
C(5)-C(6)	1.4383(15)
C(6)-C(7)	1.1952(17)
C(7)-C(8)	1.4671(16)
C(1)-N(1)-C(5)	117.32(10)
N(1)-C(1)-C(2)	124.02(11)
C(3)-C(2)-C(1)	118.37(12)
C(2)-C(3)-C(4)	118.94(11)
C(3)-C(4)-C(5)	119.25(11)
N(1)-C(5)-C(4)	122.06(11)
N(1)-C(5)-C(6)	117.11(10)
C(4)-C(5)-C(6)	120.83(10)
C(7)-C(6)-C(5)	179.30(12)
C(6)-C(7)-C(8)	177.92(12)
O(1)-C(8)-C(7)	113.43(9)

Symmetry transformations used to generate equivalent atoms:

Table S1-4. Anisotropic displacement parameters (A^2 x 10^3) for rt22.
The anisotropic displacement factor exponent takes the form:
-2 pi^2 [h^2 a*^2 U11 + ... + 2 h k a* b* U12]

	U11	U22	U33	U23	U13	U12

N(1)	72(1)	41(1)	49(1)	12(1)	3(1)	4(1)
O(1)	74(1)	44(1)	44(1)	10(1)	1(1)	14(1)
C(1)	78(1)	56(1)	47(1)	11(1)	-3(1)	-4(1)
C(2)	67(1)	67(1)	49(1)	25(1)	0(1)	8(1)
C(3)	75(1)	51(1)	55(1)	23(1)	11(1)	21(1)
C(4)	74(1)	43(1)	43(1)	10(1)	8(1)	13(1)
C(5)	52(1)	44(1)	42(1)	14(1)	8(1)	9(1)
C(6)	60(1)	45(1)	46(1)	14(1)	7(1)	13(1)
C(7)	58(1)	43(1)	48(1)	12(1)	6(1)	12(1)
C(8)	56(1)	57(1)	54(1)	18(1)	0(1)	17(1)

Table S1-5. Hydrogen coordinates (x 10^4) and isotropic displacement parameters (A^2 x 10^3) for rt22.

	x	y	z	U(eq)
H(1)	2324	1069	88	74
H(2)	1570	3528	-103	72
H(3)	3398	5544	1767	69
H(4)	5888	4995	3777	63
H(1O)	6480(40)	660(20)	7768(18)	65(4)
H(8A)	10160(50)	620(30)	6470(20)	83(5)
H(8B)	11510(50)	2290(20)	6910(20)	80(5)

Table S1-6. Torsion angles [deg] for rt22.

C(5)-N(1)-C(1)-C(2)	-1.4(2)
N(1)-C(1)-C(2)-C(3)	1.8(2)
C(1)-C(2)-C(3)-C(4)	-0.3(2)
C(2)-C(3)-C(4)-C(5)	-1.40(19)
C(1)-N(1)-C(5)-C(4)	-0.42(18)
C(1)-N(1)-C(5)-C(6)	179.26(10)
C(3)-C(4)-C(5)-N(1)	1.81(18)
C(3)-C(4)-C(5)-C(6)	-177.86(10)

Symmetry transformations used to generate equivalent atoms:

Table S1-7. Hydrogen bonds for rt22 [A and deg.].

D-H...A	d(D-H)	d(H...A)	d(D...A)	<(DHA)
O(1)-H(1O)...N(1)#1	0.89(2)	1.96(2)	2.8251(13)	165.3(15)

Symmetry transformations used to generate equivalent atoms:
#1 -x+1,-y,-z+1

Table S2-1. Crystal data and structure refinement for 1-phenylprop-2-yn-1-one (**12**).

Identification code	RT131
Empirical formula	C9 H6 O
Formula weight	130.14
Temperature	100(2) K
Wavelength	1.54184 A
Crystal system, space group	Orthorhombic, P 21 21 21
Unit cell dimensions	a = 3.8091(2) A alpha = 90 deg.
	b = 10.7199(5) A beta = 90 deg.
	c = 16.6298(9) A gamma = 90 deg.
Volume	679.05(6) A^3
Z, Calculated density	4, 1.273 Mg/m^3
Absorption coefficient	0.658 mm^-1
F(000)	272
Crystal Size	0.161 x 0.093 x 0.041 mm
Theta range for data collection	4.908 to 78.838 deg.
Limiting indices	-4<=h<=4, -13<=k<=13, -21<=l<=20
Reflections collected / unique	9502 / 1453 [R(int) = 0.0564]
Completeness to theta = 0.770	0.0 %
Absorption correction	Numerical
Max. and min. transmission	1.24 and 1.00
Refinement method	Full-matrix least-squares on F^2
Data / restraints / parameters	1453 / 0 / 91
Goodness-of-fit on F^2	1.112
Final R indices [I>2sigma(I)]	R1 = 0.0436, wR2 = 0.1252
R indices (all data)	R1 = 0.0467, wR2 = 0.1312
Extinction coefficient	n/a
Largest diff. peak and hole	0.282 and -0.191 e.A^-3

Table S2-2. Atomic coordinates (x 10^4) and equivalent isotropic displacement parameters (A^2 x 10^3) for rt131. U(eq) is defined as one third of the trace of the orthogonalized Uij tensor.

	x	y	z	U(eq)
O(1)	2085(5)	5967(1)	3312(1)	37(1)
C(1)	5986(6)	3187(2)	2825(1)	34(1)
C(2)	4873(6)	4044(2)	3204(1)	31(1)
C(3)	3479(5)	5094(2)	3663(1)	29(1)
C(4)	3777(5)	5036(2)	4549(1)	26(1)
C(5)	5215(5)	3999(2)	4938(1)	28(1)
C(6)	5389(6)	3971(2)	5771(1)	30(1)
C(7)	4164(6)	4976(2)	6220(1)	32(1)
C(8)	2760(6)	6013(2)	5832(1)	32(1)
C(9)	2549(6)	6041(2)	5003(1)	29(1)

Table S2-3. Bond lengths [A] and angles [deg] for rt131.

O(1)-C(3)	1.224(2)
C(1)-C(2)	1.193(3)
C(2)-C(3)	1.460(3)
C(3)-C(4)	1.478(3)
C(4)-C(9)	1.396(3)
C(4)-C(5)	1.398(3)
C(5)-C(6)	1.387(3)
C(6)-C(7)	1.391(3)
C(7)-C(8)	1.393(3)
C(8)-C(9)	1.381(3)
C(1)-C(2)-C(3)	179.4(2)
O(1)-C(3)-C(2)	119.87(18)
O(1)-C(3)-C(4)	122.74(17)
C(2)-C(3)-C(4)	117.38(17)
C(9)-C(4)-C(5)	119.62(18)
C(9)-C(4)-C(3)	118.71(17)
C(5)-C(4)-C(3)	121.66(16)
C(6)-C(5)-C(4)	119.88(18)
C(5)-C(6)-C(7)	120.25(18)
C(6)-C(7)-C(8)	119.80(19)
C(9)-C(8)-C(7)	120.20(18)
C(8)-C(9)-C(4)	120.23(18)

Symmetry transformations used to generate equivalent atoms:

Table S2-4. Anisotropic displacement parameters (A^2 x 10^3) for rt131.
The anisotropic displacement factor exponent takes the form:
-2 pi^2 [h^2 a*^2 U11 + ... + 2 h k a* b* U12]

U11	U22	U33	U23	U13	U12

O(1)	46(1)	29(1)	36(1)	6(1)	-2(1)	5(1)
C(1)	38(1)	34(1)	31(1)	-1(1)	0(1)	2(1)
C(2)	33(1)	31(1)	28(1)	3(1)	0(1)	0(1)
C(3)	28(1)	24(1)	33(1)	2(1)	1(1)	-2(1)
C(4)	23(1)	25(1)	31(1)	0(1)	2(1)	-3(1)
C(5)	26(1)	23(1)	33(1)	-1(1)	2(1)	0(1)
C(6)	29(1)	27(1)	34(1)	4(1)	-2(1)	-2(1)
C(7)	31(1)	37(1)	28(1)	0(1)	2(1)	-4(1)
C(8)	31(1)	28(1)	36(1)	-5(1)	5(1)	-3(1)
C(9)	27(1)	23(1)	37(1)	1(1)	2(1)	0(1)

Table S2-5. Torsion angles [deg] for rt131.

O(1)-C(3)-C(4)-C(9)	-2.7(3)
C(2)-C(3)-C(4)-C(9)	178.39(19)
O(1)-C(3)-C(4)-C(5)	176.6(2)
C(2)-C(3)-C(4)-C(5)	-2.3(3)
C(9)-C(4)-C(5)-C(6)	0.5(3)
C(3)-C(4)-C(5)-C(6)	-178.82(17)
C(4)-C(5)-C(6)-C(7)	-0.5(3)
C(5)-C(6)-C(7)-C(8)	0.0(3)
C(6)-C(7)-C(8)-C(9)	0.7(3)
C(7)-C(8)-C(9)-C(4)	-0.7(3)
C(5)-C(4)-C(9)-C(8)	0.2(3)
C(3)-C(4)-C(9)-C(8)	179.46(18)

Symmetry transformations used to generate equivalent atoms:

Table S3-1. Crystal data and structure refinement for 6-methyl-[1,2,3,4,5]pentathiepino[6,7-*a*]indolizine (**5c**)

Identification code	RT36_1
Empirical formula	C9 H7 N S5
Formula weight	289.46
Temperature	170(2) K
Wavelength	0.71073 A
Crystal system, space group	Triclinic, P -1
Unit cell dimensions	a = 7.4387(15) A alpha = 101.45(3) deg.
	b = 8.3743(17) A beta = 93.68(3) deg.
	c = 9.4594(19) A gamma = 90.50(3) deg.
Volume	576.2(2) A^3
Z, Calculated density	2, 1.668 Mg/m^3
Absorption coefficient	0.968 mm^-1
F(000)	296
Crystal Size	0.161 x 0.110 x 0.107 mm
Theta range for data collection	2.202 to 29.487 deg.
Limiting indices	-10<=h<=10, -11<=k<=11, -13<=l<=13
Reflections collected / unique	6595 / 3175 [R(int) = 0.0298]
Completeness to theta = 25.242	99.9 %
Absorption correction	Numerical
Max. and min. transmission	0.9154 and 0.8125
Refinement method	Full-matrix least-squares on F^2
Data / restraints / parameters	3175 / 0 / 164
Goodness-of-fit on F^2	1.024
Final R indices [I>2sigma(I)]	R1 = 0.0311, wR2 = 0.0696
R indices (all data)	R1 = 0.0480, wR2 = 0.0763
Extinction coefficient	n/a
Largest diff. peak and hole	0.362 and -0.263 e.A^-3

Table S3-2. Atomic coordinates (x 10^4) and equivalent isotropic displacement parameters (A^2 x 10^3) for rt36_1.
U(eq) is defined as one third of the trace of the orthogonalized Uij tensor.

	x	y	z	U(eq)
S(1)	5550(1)	8804(1)	7443(1)	29(1)
S(2)	7675(1)	9859(1)	8807(1)	34(1)
S(3)	7531(1)	8855(1)	10608(1)	37(1)
S(4)	8609(1)	6582(1)	10055(1)	35(1)
S(5)	6580(1)	5212(1)	8752(1)	31(1)
N(1)	7483(2)	5203(2)	4717(2)	22(1)
C(1)	6845(2)	6761(2)	5074(2)	24(1)
C(2)	6469(2)	7015(2)	6506(2)	24(1)
C(3)	6889(2)	5592(2)	7054(2)	23(1)
C(4)	7544(2)	4469(2)	5923(2)	23(1)
C(5)	8191(2)	2869(2)	5769(2)	28(1)
C(6)	8740(2)	2096(2)	4469(2)	33(1)
C(7)	8662(2)	2874(2)	3274(2)	33(1)
C(8)	8039(2)	4400(2)	3405(2)	27(1)
C(9)	6662(3)	7827(2)	4001(2)	32(1)

Table S3-3. Bond lengths [A] and angles [deg] for rt36_1.

S(1)-C(2)	1.7506(18)
S(1)-S(2)	2.0507(10)
S(2)-S(3)	2.0514(9)
S(3)-S(4)	2.0561(9)
S(4)-S(5)	2.0692(11)
S(5)-C(3)	1.7271(18)
N(1)-C(1)	1.378(2)
N(1)-C(8)	1.380(2)
N(1)-C(4)	1.397(2)
C(1)-C(2)	1.376(2)
C(1)-C(9)	1.480(3)
C(2)-C(3)	1.420(2)
C(3)-C(4)	1.394(2)
C(4)-C(5)	1.411(2)
C(5)-C(6)	1.360(3)
C(6)-C(7)	1.411(3)
C(7)-C(8)	1.348(3)
C(2)-S(1)-S(2)	102.85(6)
S(1)-S(2)-S(3)	104.47(4)
S(2)-S(3)-S(4)	105.03(4)
S(3)-S(4)-S(5)	103.98(4)
C(3)-S(5)-S(4)	103.75(7)
C(1)-N(1)-C(8)	128.37(15)
C(1)-N(1)-C(4)	110.09(14)
C(8)-N(1)-C(4)	121.53(15)
C(2)-C(1)-N(1)	106.99(15)
C(2)-C(1)-C(9)	131.44(16)

N(1)-C(1)-C(9)	121.57(16)
C(1)-C(2)-C(3)	109.07(15)
C(1)-C(2)-S(1)	124.08(14)
C(3)-C(2)-S(1)	126.82(13)
C(4)-C(3)-C(2)	106.83(15)
C(4)-C(3)-S(5)	124.61(13)
C(2)-C(3)-S(5)	128.49(13)
C(3)-C(4)-N(1)	107.00(14)
C(3)-C(4)-C(5)	134.76(17)
N(1)-C(4)-C(5)	118.24(16)
C(6)-C(5)-C(4)	119.55(18)
C(5)-C(6)-C(7)	120.69(17)
C(8)-C(7)-C(6)	120.36(18)
C(7)-C(8)-N(1)	119.62(18)

Symmetry transformations used to generate equivalent atoms:

Table S3-4. Anisotropic displacement parameters (A^2 x 10^3) for rt36_1.
The anisotropic displacement factor exponent takes the form:
-2 pi^2 [h^2 a*^2 U11 + ... + 2 h k a* b* U12]

	U11	U22	U33	U23	U13	U12
S(1)	27(1)	26(1)	33(1)	1(1)	0(1)	8(1)
S(2)	31(1)	26(1)	42(1)	-1(1)	0(1)	0(1)
S(3)	38(1)	40(1)	29(1)	-5(1)	0(1)	11(1)
S(4)	38(1)	40(1)	25(1)	4(1)	-1(1)	13(1)
S(5)	38(1)	33(1)	26(1)	9(1)	8(1)	1(1)
N(1)	23(1)	22(1)	21(1)	3(1)	1(1)	0(1)
C(1)	23(1)	23(1)	25(1)	5(1)	1(1)	1(1)
C(2)	22(1)	22(1)	26(1)	4(1)	1(1)	2(1)
C(3)	23(1)	24(1)	23(1)	6(1)	3(1)	1(1)
C(4)	20(1)	23(1)	25(1)	5(1)	1(1)	-2(1)
C(5)	28(1)	23(1)	35(1)	8(1)	3(1)	2(1)
C(6)	29(1)	22(1)	44(1)	-1(1)	4(1)	1(1)
C(7)	29(1)	31(1)	34(1)	-4(1)	7(1)	-2(1)
C(8)	28(1)	29(1)	23(1)	2(1)	4(1)	-2(1)
C(9)	36(1)	29(1)	31(1)	11(1)	-1(1)	0(1)

Table S3-5. Torsion angles [deg] for rt36_1.

C(8)-N(1)-C(1)-C(2)	-179.84(16)
C(4)-N(1)-C(1)-C(2)	-0.98(18)
C(8)-N(1)-C(1)-C(9)	0.4(3)
C(4)-N(1)-C(1)-C(9)	179.28(16)
N(1)-C(1)-C(2)-C(3)	0.33(19)
C(9)-C(1)-C(2)-C(3)	-179.98(18)
N(1)-C(1)-C(2)-S(1)	-177.78(12)
C(9)-C(1)-C(2)-S(1)	1.9(3)
S(2)-S(1)-C(2)-C(1)	-108.80(14)
S(2)-S(1)-C(2)-C(3)	73.43(15)

C(1)-C(2)-C(3)-C(4)	0.44(19)
S(1)-C(2)-C(3)-C(4)	178.48(12)
C(1)-C(2)-C(3)-S(5)	-176.68(13)
S(1)-C(2)-C(3)-S(5)	1.4(2)
S(4)-S(5)-C(3)-C(4)	108.38(14)
S(4)-S(5)-C(3)-C(2)	-74.97(16)
C(2)-C(3)-C(4)-N(1)	-1.02(18)
S(5)-C(3)-C(4)-N(1)	176.24(12)
C(2)-C(3)-C(4)-C(5)	179.37(18)
S(5)-C(3)-C(4)-C(5)	-3.4(3)
C(1)-N(1)-C(4)-C(3)	1.26(17)
C(8)-N(1)-C(4)-C(3)	-179.79(15)
C(1)-N(1)-C(4)-C(5)	-179.05(15)
C(8)-N(1)-C(4)-C(5)	-0.1(2)
C(3)-C(4)-C(5)-C(6)	179.89(18)
N(1)-C(4)-C(5)-C(6)	0.3(2)
C(4)-C(5)-C(6)-C(7)	-0.3(3)
C(5)-C(6)-C(7)-C(8)	0.0(3)
C(6)-C(7)-C(8)-N(1)	0.2(3)
C(1)-N(1)-C(8)-C(7)	178.59(16)
C(4)-N(1)-C(8)-C(7)	-0.2(2)

Table S4-1. Crystal data and structure refinement for 6-phenyl-[1,2,3,4,5]pentathiepino[6,7-*a*]indolizine (**5d**)

Identification code	RT153
Empirical formula	C14 H9 N S5
Formula weight	351.52
Temperature	100(2) K
Wavelength	1.54184 A
Crystal system, space group	Monoclinic, P 21/c
Unit cell dimensions	a = 8.82170(10) A alpha = 90 deg.
	b = 17.9450(2) A beta = 92.1550(10) deg.
	c = 9.15640(10) A gamma = 90 deg.
Volume	1448.48(3) A^3
Z, Calculated density	4, 1.612 Mg/m^3
Absorption coefficient	7.259 mm^-1
F(000)	720
Crystal Size	0.268 x 0.092 x 0.082 mm
Theta range for data collection	6.912 to 70.067 deg.
Limiting indices	-10<=h<=10, -21<=k<=21, -10<=l<=11
Reflections collected / unique	37091 / 2747 [R(int) = 0.0421]
Completeness to theta = 0.770	0.0 %
Absorption correction	Numerical
Max. and min. transmission	0.99 and 0.33
Refinement method	Full-matrix least-squares on F^2
Data / restraints / parameters	2747/ 0 / 181
Goodness-of-fit on F^2	1.039
Final R indices [I>2sigma(I)]	R1 = 0.0220, wR2 = 0.0578
R indices (all data)	R1 = 0.0224, wR2 = 0.0581
Extinction coefficient	n/a
Largest diff. peak and hole	0.322 and -0.285 e.A^-3

Table S4-2. Atomic coordinates (x 10^4) and equivalent isotropic displacement parameters (A^2 x 10^3) for rt153.
U(eq) is defined as one third of the trace of the orthogonalized Uij tensor.

	x	y	z	U(eq)
S(1)	6211(1)	3498(1)	7555(1)	20(1)
S(2)	6427(1)	3563(1)	5324(1)	20(1)
S(3)	8276(1)	4246(1)	5041(1)	21(1)
S(4)	7461(1)	5304(1)	5319(1)	20(1)
S(5)	7287(1)	5405(1)	7561(1)	20(1)
N(1)	3051(1)	4957(1)	8455(1)	15(1)
C(1)	3554(2)	4229(1)	8288(2)	16(1)
C(2)	5068(2)	4270(1)	7932(2)	16(1)
C(3)	5507(2)	5032(1)	7876(1)	17(1)
C(4)	4236(2)	5456(1)	8185(2)	17(1)
C(5)	3957(2)	6230(1)	8287(2)	20(1)
C(6)	2579(2)	6477(1)	8694(2)	23(1)
C(7)	1403(2)	5958(1)	8989(2)	22(1)
C(8)	1648(2)	5216(1)	8864(2)	19(1)
C(9)	2588(2)	3573(1)	8474(2)	16(1)
C(10)	1134(2)	3526(1)	7823(2)	20(1)
C(11)	276(2)	2885(1)	7974(2)	27(1)
C(12)	846(2)	2283(1)	8765(2)	31(1)
C(13)	2287(2)	2323(1)	9422(2)	28(1)
C(14)	3153(2)	2962(1)	9279(2)	21(1)

Table S4-3. Bond lengths [A] and angles [deg] for rt153.

S(1)-C(2)	1.7559(14)
S(1)-S(2)	2.0622(5)
S(2)-S(3)	2.0640(5)
S(3)-S(4)	2.0497(5)
S(4)-S(5)	2.0717(5)
S(5)-C(3)	1.7406(14)
N(1)-C(8)	1.3865(18)
N(1)-C(1)	1.3913(18)
N(1)-C(4)	1.4045(18)
C(1)-C(2)	1.3885(19)
C(1)-C(9)	1.4659(19)
C(2)-C(3)	1.4226(19)
C(3)-C(4)	1.392(2)
C(4)-C(5)	1.415(2)
C(5)-C(6)	1.359(2)
C(6)-C(7)	1.429(2)
C(7)-C(8)	1.353(2)
C(9)-C(10)	1.398(2)
C(9)-C(14)	1.403(2)
C(10)-C(11)	1.387(2)
C(11)-C(12)	1.385(2)
C(12)-C(13)	1.388(2)
C(13)-C(14)	1.387(2)

C(2)-S(1)-S(2)	103.01(5)
S(1)-S(2)-S(3)	105.12(2)
S(4)-S(3)-S(2)	104.60(2)
S(3)-S(4)-S(5)	104.08(2)
C(3)-S(5)-S(4)	103.40(5)
C(8)-N(1)-C(1)	129.53(12)
C(8)-N(1)-C(4)	120.80(12)
C(1)-N(1)-C(4)	109.64(11)
C(2)-C(1)-N(1)	106.80(12)
C(2)-C(1)-C(9)	129.70(13)
N(1)-C(1)-C(9)	123.50(12)
C(1)-C(2)-C(3)	108.99(12)
C(1)-C(2)-S(1)	124.63(11)
C(3)-C(2)-S(1)	126.35(11)
C(4)-C(3)-C(2)	107.16(12)
C(4)-C(3)-S(5)	124.24(11)
C(2)-C(3)-S(5)	128.56(11)
C(3)-C(4)-N(1)	107.39(12)
C(3)-C(4)-C(5)	133.85(13)
N(1)-C(4)-C(5)	118.74(13)
C(6)-C(5)-C(4)	119.90(13)
C(5)-C(6)-C(7)	120.13(13)
C(8)-C(7)-C(6)	120.41(14)
C(7)-C(8)-N(1)	119.97(13)
C(10)-C(9)-C(14)	118.64(13)
C(10)-C(9)-C(1)	121.82(13)
C(14)-C(9)-C(1)	119.49(13)
C(11)-C(10)-C(9)	120.21(14)
C(12)-C(11)-C(10)	120.74(14)
C(11)-C(12)-C(13)	119.66(14)
C(14)-C(13)-C(12)	120.05(15)
C(13)-C(14)-C(9)	120.70(14)

Symmetry transformations used to generate equivalent atoms:

Table S4-4. Anisotropic displacement parameters (A^2 x 10^3) for rt153.
The anisotropic displacement factor exponent takes the form:
-2 pi^2 [h^2 a*^2 U11 + ... + 2 h k a* b* U12]

	U11	U22	U33	U23	U13	U12
S(1)	19(1)	18(1)	23(1)	5(1)	4(1)	5(1)
S(2)	20(1)	17(1)	22(1)	-1(1)	2(1)	1(1)
S(3)	17(1)	24(1)	22(1)	1(1)	3(1)	2(1)
S(4)	22(1)	19(1)	20(1)	3(1)	3(1)	-2(1)
S(5)	18(1)	23(1)	20(1)	0(1)	1(1)	-8(1)
N(1)	17(1)	13(1)	16(1)	1(1)	2(1)	-1(1)
C(1)	17(1)	14(1)	15(1)	1(1)	1(1)	0(1)
C(2)	17(1)	15(1)	16(1)	2(1)	0(1)	1(1)
C(3)	16(1)	17(1)	17(1)	2(1)	1(1)	-4(1)
C(4)	20(1)	15(1)	14(1)	1(1)	0(1)	-4(1)
C(5)	28(1)	14(1)	17(1)	0(1)	0(1)	-5(1)

C(6)	34(1)	13(1)	20(1)	-1(1)	0(1)	2(1)
C(7)	26(1)	21(1)	20(1)	-1(1)	5(1)	5(1)
C(8)	20(1)	19(1)	18(1)	0(1)	6(1)	0(1)
C(9)	18(1)	14(1)	16(1)	-1(1)	4(1)	-1(1)
C(10)	18(1)	19(1)	24(1)	-1(1)	2(1)	2(1)
C(11)	17(1)	26(1)	39(1)	-6(1)	2(1)	-4(1)
C(12)	28(1)	19(1)	45(1)	-1(1)	10(1)	-10(1)
C(13)	34(1)	17(1)	33(1)	7(1)	3(1)	-3(1)
C(14)	23(1)	18(1)	23(1)	3(1)	-1(1)	-2(1)

Table S4-5. Torsion angles [deg] for rt153.

C(8)-N(1)-C(1)-C(2)	-177.20(13)
C(4)-N(1)-C(1)-C(2)	0.82(15)
C(8)-N(1)-C(1)-C(9)	2.8(2)
C(4)-N(1)-C(1)-C(9)	-179.18(12)
N(1)-C(1)-C(2)-C(3)	-0.07(16)
C(9)-C(1)-C(2)-C(3)	179.94(13)
N(1)-C(1)-C(2)-S(1)	-178.06(10)
C(9)-C(1)-C(2)-S(1)	2.0(2)
S(2)-S(1)-C(2)-C(1)	106.91(12)
S(2)-S(1)-C(2)-C(3)	-70.72(13)
C(1)-C(2)-C(3)-C(4)	-0.70(16)
S(1)-C(2)-C(3)-C(4)	177.24(10)
C(1)-C(2)-C(3)-S(5)	176.91(10)
S(1)-C(2)-C(3)-S(5)	-5.1(2)
S(4)-S(5)-C(3)-C(4)	-105.06(12)
S(4)-S(5)-C(3)-C(2)	77.70(13)
C(2)-C(3)-C(4)-N(1)	1.19(15)
S(5)-C(3)-C(4)-N(1)	-176.55(10)
C(2)-C(3)-C(4)-C(5)	-179.80(15)
S(5)-C(3)-C(4)-C(5)	2.5(2)
C(8)-N(1)-C(4)-C(3)	176.96(12)
C(1)-N(1)-C(4)-C(3)	-1.27(15)
C(8)-N(1)-C(4)-C(5)	-2.23(19)
C(1)-N(1)-C(4)-C(5)	179.55(12)
C(3)-C(4)-C(5)-C(6)	-176.47(15)
N(1)-C(4)-C(5)-C(6)	2.4(2)
C(4)-C(5)-C(6)-C(7)	-1.3(2)
C(5)-C(6)-C(7)-C(8)	-0.2(2)
C(6)-C(7)-C(8)-N(1)	0.4(2)
C(1)-N(1)-C(8)-C(7)	178.64(14)
C(4)-N(1)-C(8)-C(7)	0.8(2)
C(2)-C(1)-C(9)-C(10)	-132.55(16)
N(1)-C(1)-C(9)-C(10)	47.5(2)
C(2)-C(1)-C(9)-C(14)	44.9(2)
N(1)-C(1)-C(9)-C(14)	-135.12(14)
C(14)-C(9)-C(10)-C(11)	-0.1(2)
C(1)-C(9)-C(10)-C(11)	177.35(14)
C(9)-C(10)-C(11)-C(12)	-0.2(2)
C(10)-C(11)-C(12)-C(13)	0.4(2)
C(11)-C(12)-C(13)-C(14)	-0.3(3)
C(12)-C(13)-C(14)-C(9)	0.0(2)
C(10)-C(9)-C(14)-C(13)	0.2(2)
C(1)-C(9)-C(14)-C(13)	-177.32(14)

Table S5-1. Crystal data and structure refinement for 2-([1,2,3,4,5]pentathiepino[6,7-*a*]indolizin-6-yloxy)ethan-1-ol (**5e**).

Identification code	RT-85-5
Empirical formula	C10 H9 N O2 S5
Formula weight	335,48
Temperature	298(2) K
Wavelength	1.54184 A
Crystal system, space group	Triclinic, P -1
Unit cell dimensions	a = 4.44950(10) A alpha = 75.633(2) deg.
	b = 9.7780(2) A beta = 89.894(2) deg.
	c = 15.7164(3) A gamma = 85.165(2) deg.
Volume	659.91(2) A^3
Z, Calculated density	2, 1.688 Mg/m^3
Absorption coefficient	8.042 mm^-1
F(000)	344
Crystal Size	0.180 x 0.050 x 0.040 mm
Theta range for data collection	4.686 to 79.968 deg.
Limiting indices	-5<=h<=5, -12<=k<=12, -19<=l<=20
Reflections collected / unique	19302 / 2810 [R(int) = 0.0452]
Completeness to theta = 0.770	0.0 %
Absorption correction	Numerical
Max. and min. transmission	1.000 and 0.576

Refinement method	Full-matrix least-squares on F^2
Data / restraints / parameters	2810 / 1 / 167
Goodness-of-fit on F^2	1.103
Final R indices [I>2sigma(I)]	R1 = 0.0427, wR2 = 0.1171
R indices (all data)	R1 = 0.0453, wR2 = 0.1198
Extinction coefficient	n/a
Largest diff. peak and hole	0.972 and -0.336 e.A^-3

Table S5-2. Atomic coordinates (x 10^4) and equivalent isotropic displacement parameters (A^2 x 10^3) for RT-85-5.
U(eq) is defined as one third of the trace of the orthogonalized Uij tensor.

	x	y	z	U(eq)
S(1)	9707(2)	2359(1)	9030(1)	46(1)
S(2)	7169(2)	3989(1)	9391(1)	48(1)
S(3)	8882(2)	5788(1)	8669(1)	52(1)
S(4)	7001(2)	6078(1)	7437(1)	47(1)
S(5)	9481(2)	4661(1)	6879(1)	44(1)
O(1)	5040(4)	2969(2)	5868(1)	38(1)
O(2)	7387(7)	708(2)	5111(2)	67(1)
N(1)	5013(5)	1274(2)	7216(1)	33(1)
C(1)	3188(6)	353(3)	6969(2)	41(1)
C(2)	2405(7)	-779(3)	7578(2)	51(1)
C(3)	3453(8)	-1046(3)	8464(2)	53(1)
C(4)	5237(7)	-142(3)	8710(2)	45(1)
C(5)	6068(6)	1064(3)	8084(2)	36(1)
C(6)	7803(6)	2180(3)	8104(2)	37(1)
C(7)	7717(6)	3094(2)	7242(2)	35(1)
C(8)	5980(5)	2515(2)	6715(2)	33(1)
C(9)	7342(6)	3125(3)	5208(2)	38(1)
C(10)	6921(7)	2151(3)	4629(2)	45(1)

Table S5-3. Bond lengths [A] and angles [deg] for RT-85-5.

S(1)-C(6)	1.738(3)
S(1)-S(2)	2.0691(9)
S(2)-S(3)	2.0484(10)
S(3)-S(4)	2.0548(10)
S(4)-S(5)	2.0611(10)
S(5)-C(7)	1.748(2)

O(1)-C(8)	1.350(3)
O(1)-C(9)	1.447(3)
O(2)-C(10)	1.425(4)
N(1)-C(8)	1.374(3)
N(1)-C(1)	1.385(3)
N(1)-C(5)	1.404(3)
C(1)-C(2)	1.341(4)
C(2)-C(3)	1.424(4)
C(3)-C(4)	1.359(4)
C(4)-C(5)	1.412(4)
C(5)-C(6)	1.395(4)
C(6)-C(7)	1.425(3)
C(7)-C(8)	1.383(3)
C(9)-C(10)	1.494(4)
C(6)-S(1)-S(2)	103.56(9)
S(3)-S(2)-S(1)	104.13(4)
S(2)-S(3)-S(4)	104.12(4)
S(3)-S(4)-S(5)	105.14(4)
C(7)-S(5)-S(4)	103.57(8)
C(8)-O(1)-C(9)	117.02(19)
C(8)-N(1)-C(1)	128.8(2)
C(8)-N(1)-C(5)	109.01(19)
C(1)-N(1)-C(5)	122.1(2)
C(2)-C(1)-N(1)	119.0(3)
C(1)-C(2)-C(3)	120.8(3)
C(4)-C(3)-C(2)	120.3(3)
C(3)-C(4)-C(5)	120.0(3)
C(6)-C(5)-N(1)	107.3(2)
C(6)-C(5)-C(4)	135.0(2)
N(1)-C(5)-C(4)	117.7(2)
C(5)-C(6)-C(7)	107.4(2)
C(5)-C(6)-S(1)	124.66(19)
C(7)-C(6)-S(1)	127.92(19)
C(8)-C(7)-C(6)	107.6(2)
C(8)-C(7)-S(5)	124.66(19)
C(6)-C(7)-S(5)	127.70(19)
O(1)-C(8)-N(1)	118.7(2)
O(1)-C(8)-C(7)	132.6(2)
N(1)-C(8)-C(7)	108.6(2)
O(1)-C(9)-C(10)	109.8(2)
O(2)-C(10)-C(9)	110.9(2)

Symmetry transformations used to generate equivalent atoms:

Table S5-4. Anisotropic displacement parameters (A^2 x 10^3) for RT-85-5.
The anisotropic displacement factor exponent takes the form:
-2 pi^2 [h^2 a*^2 U11 + ... + 2 h k a* b* U12]

	U11	U22	U33	U23	U13	U12
S(1)	61(1)	42(1)	36(1)	-12(1)	-11(1)	2(1)
S(2)	69(1)	43(1)	34(1)	-14(1)	5(1)	-6(1)

S(3)	76(1)	41(1)	42(1)	-15(1)	-5(1)	-15(1)
S(4)	67(1)	33(1)	40(1)	-8(1)	-3(1)	-6(1)
S(5)	57(1)	41(1)	39(1)	-11(1)	12(1)	-19(1)
O(1)	43(1)	46(1)	25(1)	-6(1)	4(1)	-4(1)
O(2)	94(2)	40(1)	71(2)	-20(1)	23(1)	-15(1)
N(1)	44(1)	29(1)	28(1)	-8(1)	6(1)	-5(1)
C(1)	50(1)	39(1)	40(1)	-16(1)	7(1)	-12(1)
C(2)	64(2)	41(1)	52(2)	-17(1)	13(1)	-19(1)
C(3)	77(2)	34(1)	47(2)	-4(1)	17(1)	-15(1)
C(4)	67(2)	33(1)	32(1)	-2(1)	7(1)	-3(1)
C(5)	51(1)	30(1)	27(1)	-8(1)	5(1)	-2(1)
C(6)	50(1)	32(1)	29(1)	-9(1)	2(1)	-4(1)
C(7)	45(1)	30(1)	30(1)	-9(1)	4(1)	-6(1)
C(8)	43(1)	29(1)	25(1)	-6(1)	3(1)	-4(1)
C(9)	50(1)	35(1)	29(1)	-4(1)	9(1)	-11(1)
C(10)	57(2)	49(2)	30(1)	-11(1)	5(1)	-9(1)

Table S5-5. Hydrogen coordinates (x 10^4) and isotropic displacement parameters (A^2 x 10^3) for RT-85-5.

	x	y	z	U(eq)
H(1)	2515	516	6390	50
H(2A)	1163	-1397	7419	61
H(3)	2915	-1842	8877	63
H(4)	5910	-319	9290	54
H(9A)	9321	2905	5490	46
H(9B)	7215	4097	4858	46
H(10A)	4895	2335	4377	54
H(10B)	8335	2330	4152	54
H(2O)	7300(200)	120(100)	4880(70)	290(60)

Table S5-6. Torsion angles [deg] for RT-85-5.

C(8)-N(1)-C(1)-C(2)	-177.1(2)
C(5)-N(1)-C(1)-C(2)	-0.1(4)
N(1)-C(1)-C(2)-C(3)	-0.7(4)
C(1)-C(2)-C(3)-C(4)	0.9(5)
C(2)-C(3)-C(4)-C(5)	-0.2(4)
C(8)-N(1)-C(5)-C(6)	-2.0(3)
C(1)-N(1)-C(5)-C(6)	-179.5(2)
C(8)-N(1)-C(5)-C(4)	178.3(2)
C(1)-N(1)-C(5)-C(4)	0.8(4)
C(3)-C(4)-C(5)-C(6)	179.8(3)
C(3)-C(4)-C(5)-N(1)	-0.6(4)
N(1)-C(5)-C(6)-C(7)	1.7(3)
C(4)-C(5)-C(6)-C(7)	-178.7(3)
N(1)-C(5)-C(6)-S(1)	-177.72(17)
C(4)-C(5)-C(6)-S(1)	1.9(4)
S(2)-S(1)-C(6)-C(5)	-106.8(2)
S(2)-S(1)-C(6)-C(7)	73.9(2)
C(5)-C(6)-C(7)-C(8)	-0.7(3)

S(1)-C(6)-C(7)-C(8)	178.62(19)
C(5)-C(6)-C(7)-S(5)	179.99(19)
S(1)-C(6)-C(7)-S(5)	-0.6(4)
S(4)-S(5)-C(7)-C(8)	108.5(2)
S(4)-S(5)-C(7)-C(6)	-72.3(2)
C(9)-O(1)-C(8)-N(1)	-119.9(2)
C(9)-O(1)-C(8)-C(7)	63.9(3)
C(1)-N(1)-C(8)-O(1)	1.8(4)
C(5)-N(1)-C(8)-O(1)	-175.5(2)
C(1)-N(1)-C(8)-C(7)	178.8(2)
C(5)-N(1)-C(8)-C(7)	1.6(3)
C(6)-C(7)-C(8)-O(1)	176.0(2)
S(5)-C(7)-C(8)-O(1)	-4.7(4)
C(6)-C(7)-C(8)-N(1)	-0.5(3)
S(5)-C(7)-C(8)-N(1)	178.79(17)
C(8)-O(1)-C(9)-C(10)	118.2(2)
O(1)-C(9)-C(10)-O(2)	-65.0(3)

Symmetry transformations used to generate equivalent atoms:

Table S5-7. Hydrogen bonds for RT-85-5 [A and deg.].

D-H...A	d(D-H)	d(H...A)	d(D...A)	<(DHA)
C(10)-H(10A)...S(4)#1	0.97	2.97	3.648(3)	127.7
O(2)-H(2O)...O(2)#2	0.75(5)	2.28(10)	2.700(6)	116(9)

Symmetry transformations used to generate equivalent atoms:
#1 -x+1,-y+1,-z+1 #2 -x+1,-y,-z+1

Table S6-1. Crystal data and structure refinement for 3-([1,2,3,4,5]pentathiepino[6,7-*a*]indolizin-6-yloxy)propan-1-ol (**5f**).

Identification code	RT173
Empirical formula	C11 H11 N O2 S5
Formula weight	349.51
Temperature	102(2) K
Wavelength	1.54184 A
Crystal system, space group	Orthorhombic, P b c n
Unit cell dimensions	a = 20.78420(10) A alpha = 90 deg.
	b = 17.44680(10) A beta = 90 deg.
	c = 7.81130(10) A gamma = 90 deg
Volume	2832.52(4) A^3
Z, Calculated density	8, 1.639 Mg/m^3
Absorption coefficient	8.042 mm^-1
F(000)	1440
Crystal Size	0.150 x 0.070 x 0.050 mm
Theta range for data collection	3.307 to 66.596 deg.
Limiting indices	-24<=h<=24, -20<=k<=20, -9<=l<=8
Reflections collected / unique	86889 / 2515 [R(int) = 0.0471]
Completeness to theta = 0.770	0.0 %
Absorption correction	Numerical
Max. and min. transmission	0.889 and 0.564
Refinement method	Full-matrix least-squares on F^2
Data / restraints / parameters	2515 / 5 / 215
Goodness-of-fit on F^2	1.337
Final R indices [I>2sigma(I)]	R1 = 0.0521, wR2 = 0.1312
R indices (all data)	R1 = 0.0524, wR2 = 0.1312
Extinction coefficient	n/a
Largest diff. peak and hole	0.527 and -0.470 e.A^-3

Table S6-2. Atomic coordinates (x 10^4) and equivalent isotropic displacement parameters (A^2 x 10^3) for rt173.
U(eq) is defined as one third of the trace of the orthogonalized Uij tensor.

	x	y	z	U(eq)
S(1)	5928(1)	8543(1)	6628(2)	20(1)
S(2)	5299(1)	8900(1)	4749(2)	21(1)
S(3)	5844(1)	9616(1)	3230(2)	24(1)
S(4)	6360(1)	8895(1)	1667(2)	23(1)
S(5)	7085(1)	8478(1)	3234(2)	20(1)
O(1)	5883(2)	6671(2)	7578(5)	22(1)
N(2)	6617(2)	6521(2)	5362(5)	16(1)
C(1)	6232(2)	6994(3)	6324(6)	15(1)
C(2)	6295(2)	7731(3)	5730(7)	18(1)
C(3)	6747(2)	7710(3)	4328(6)	16(1)
C(4)	6931(2)	6958(3)	4105(6)	14(1)
C(5)	7355(2)	6567(3)	2972(6)	19(1)
C(6)	7454(3)	5798(3)	3157(7)	23(1)
C(7)	7122(2)	5385(3)	4468(7)	23(1)
C(8)	6715(2)	5741(3)	5547(7)	19(1)
C(9)	5671(6)	7060(6)	9080(14)	25(3)
C(10)	5796(6)	6556(7)	10602(14)	24(3)
C(11)	5488(6)	5774(8)	10480(19)	23(3)
O(2)	4798(4)	5815(5)	10480(10)	20(2)
C(9')	6099(5)	6862(6)	9344(11)	19(2)
C(10')	5502(5)	6834(6)	10414(13)	20(3)
C(11')	5157(7)	6071(6)	10384(13)	24(3)
O(2')	5514(4)	5484(5)	11245(13)	25(2)

Table S6-3. Bond lengths [A] and angles [deg] for rt173.

S(1)-C(2)	1.755(5)
S(1)-S(2)	2.0627(18)
S(2)-S(3)	2.0615(18)
S(3)-S(4)	2.0549(19)
S(4)-S(5)	2.0739(18)
S(5)-C(3)	1.739(5)
O(1)-C(1)	1.343(6)
O(1)-C(9)	1.426(11)
O(1)-C(9')	1.489(10)
N(2)-C(1)	1.372(6)
N(2)-C(8)	1.384(6)
N(2)-C(4)	1.404(6)
C(1)-C(2)	1.374(7)
C(2)-C(3)	1.442(7)
C(3)-C(4)	1.376(7)
C(4)-C(5)	1.423(7)
C(5)-C(6)	1.364(7)
C(6)-C(7)	1.429(8)
C(7)-C(8)	1.345(7)
C(9)-C(10)	1.502(14)

C(10)-C(11)	1.510(14)
C(11)-O(2)	1.435(12)
C(9')-C(10')	1.498(12)
C(10')-C(11')	1.512(14)
C(11')-O(2')	1.432(12)
C(2)-S(1)-S(2)	103.59(18)
S(3)-S(2)-S(1)	104.12(7)
S(4)-S(3)-S(2)	104.97(7)
S(3)-S(4)-S(5)	104.09(8)
C(3)-S(5)-S(4)	105.42(17)
C(1)-O(1)-C(9)	124.5(5)
C(1)-O(1)-C(9')	114.7(5)
C(1)-N(2)-C(8)	128.3(4)
C(1)-N(2)-C(4)	109.1(4)
C(8)-N(2)-C(4)	122.6(4)
O(1)-C(1)-N(2)	117.5(4)
O(1)-C(1)-C(2)	133.7(5)
N(2)-C(1)-C(2)	108.8(4)
C(1)-C(2)-C(3)	107.1(4)
C(1)-C(2)-S(1)	125.4(4)
C(3)-C(2)-S(1)	127.4(4)
C(4)-C(3)-C(2)	107.6(4)
C(4)-C(3)-S(5)	124.0(4)
C(2)-C(3)-S(5)	128.0(4)
C(3)-C(4)-N(2)	107.4(4)
C(3)-C(4)-C(5)	135.1(5)
N(2)-C(4)-C(5)	117.5(4)
C(6)-C(5)-C(4)	120.0(5)
C(5)-C(6)-C(7)	119.9(5)
C(8)-C(7)-C(6)	121.2(5)
C(7)-C(8)-N(2)	118.8(5)
O(1)-C(9)-C(10)	108.5(9)
C(9)-C(10)-C(11)	114.0(10)
O(2)-C(11)-C(10)	112.3(12)
O(1)-C(9')-C(10')	105.0(8)
C(9')-C(10')-C(11')	114.4(9)
O(2')-C(11')-C(10')	112.2(11)

Symmetry transformations used to generate equivalent atoms:

Table S6-4. Anisotropic displacement parameters (A^2 x 10^3) for rt173.
The anisotropic displacement factor exponent takes the form:
-2 pi^2 [h^2 a*^2 U11 + ... + 2 h k a* b* U12]

	U11	U22	U33	U23	U13	U12
S(1)	23(1)	18(1)	17(1)	-3(1)	3(1)	1(1)
S(2)	20(1)	19(1)	25(1)	1(1)	0(1)	2(1)
S(3)	26(1)	16(1)	30(1)	5(1)	0(1)	0(1)
S(4)	26(1)	24(1)	20(1)	6(1)	-2(1)	-1(1)
S(5)	19(1)	19(1)	22(1)	4(1)	2(1)	-5(1)
O(1)	29(2)	24(2)	14(2)	1(2)	5(2)	-7(2)

N(2)	15(2)	17(2)	16(2)	-1(2)	-3(2)	-1(2)
C(1)	15(2)	19(2)	11(2)	1(2)	2(2)	-1(2)
C(2)	18(2)	14(2)	20(3)	-1(2)	-2(2)	-2(2)
C(3)	14(2)	18(2)	15(2)	2(2)	-1(2)	-3(2)
C(4)	11(2)	16(2)	15(2)	1(2)	-3(2)	-3(2)
C(5)	17(2)	24(3)	15(2)	-2(2)	3(2)	-1(2)
C(6)	21(2)	24(3)	25(3)	-6(2)	-4(2)	3(2)
C(7)	23(3)	18(2)	27(3)	-4(2)	-6(2)	2(2)
C(8)	24(2)	11(2)	21(3)	2(2)	-5(2)	-4(2)
C(9)	34(8)	18(6)	24(7)	-8(5)	15(6)	-9(5)
C(10)	25(7)	34(7)	14(6)	-3(5)	-7(5)	3(6)
C(11)	23(7)	32(9)	16(7)	2(6)	14(6)	5(7)
O(2)	19(5)	21(4)	21(4)	-1(3)	-2(4)	1(4)
C(9')	23(6)	26(5)	7(5)	0(4)	-5(4)	-3(4)
C(10')	25(6)	22(6)	13(5)	0(4)	4(5)	5(5)
C(11')	27(8)	31(7)	13(5)	2(5)	7(5)	6(7)
O(2')	28(4)	26(5)	21(5)	-5(4)	1(4)	0(3)

Table S6-5. Hydrogen coordinates (x 10^4) and isotropic displacement parameters (A^2 x 10^3) for rt173.

	x	y	z	U(eq)
H(5)	7568	6840	2088	22
H(6)	7743	5538	2416	28
H(7)	7189	4849	4579	27
H(8)	6500	5462	6420	22
H(9A)	5206	7175	8993	31
H(9B)	5906	7551	9206	31
H(10A)	5635	6816	11642	29
H(10B)	6267	6490	10734	29
H(11A)	5633	5519	9416	28
H(11B)	5631	5457	11460	28
H(2O)	4620(60)	5460(70)	9530(170)	30
H(9C)	6294	7379	9380	22
H(9D)	6419	6484	9755	22
H(10C)	5202	7235	10008	24
H(10D)	5617	6957	11613	24
H(11C)	4732	6127	10939	28
H(11D)	5085	5915	9181	28
H(2O')	5410(60)	5530(70)	12360(190)	38

Table S6-6. Torsion angles [deg] for rt173.

C(9)-O(1)-C(1)-N(2)	153.7(8)
C(9')-O(1)-C(1)-N(2)	109.8(6)
C(9)-O(1)-C(1)-C(2)	-27.1(11)
C(9')-O(1)-C(1)-C(2)	-71.0(8)
C(8)-N(2)-C(1)-O(1)	-2.7(7)
C(4)-N(2)-C(1)-O(1)	178.8(4)
C(8)-N(2)-C(1)-C(2)	177.9(5)
C(4)-N(2)-C(1)-C(2)	-0.6(5)

O(1)-C(1)-C(2)-C(3)	-179.5(5)
N(2)-C(1)-C(2)-C(3)	-0.3(5)
O(1)-C(1)-C(2)-S(1)	4.2(9)
N(2)-C(1)-C(2)-S(1)	-176.6(3)
S(2)-S(1)-C(2)-C(1)	-114.4(4)
S(2)-S(1)-C(2)-C(3)	70.1(4)
C(1)-C(2)-C(3)-C(4)	1.1(6)
S(1)-C(2)-C(3)-C(4)	177.3(4)
C(1)-C(2)-C(3)-S(5)	-172.0(4)
S(1)-C(2)-C(3)-S(5)	4.2(7)
S(4)-S(5)-C(3)-C(4)	113.0(4)
S(4)-S(5)-C(3)-C(2)	-74.9(5)
C(2)-C(3)-C(4)-N(2)	-1.5(5)
S(5)-C(3)-C(4)-N(2)	172.0(3)
C(2)-C(3)-C(4)-C(5)	-179.8(5)
S(5)-C(3)-C(4)-C(5)	-6.3(8)
C(1)-N(2)-C(4)-C(3)	1.3(5)
C(8)-N(2)-C(4)-C(3)	-177.3(4)
C(1)-N(2)-C(4)-C(5)	180.0(4)
C(8)-N(2)-C(4)-C(5)	1.3(7)
C(3)-C(4)-C(5)-C(6)	176.7(5)
N(2)-C(4)-C(5)-C(6)	-1.5(7)
C(4)-C(5)-C(6)-C(7)	1.2(8)
C(5)-C(6)-C(7)-C(8)	-0.8(8)
C(6)-C(7)-C(8)-N(2)	0.6(8)
C(1)-N(2)-C(8)-C(7)	-179.2(5)
C(4)-N(2)-C(8)-C(7)	-0.9(7)
C(1)-O(1)-C(9)-C(10)	-134.6(8)
O(1)-C(9)-C(10)-C(11)	-56.3(14)
C(9)-C(10)-C(11)-O(2)	-65.1(15)
C(1)-O(1)-C(9')-C(10')	150.1(7)
O(1)-C(9')-C(10')-C(11')	58.3(12)
C(9')-C(10')-C(11')-O(2')	69.2(12)

Symmetry transformations used to generate equivalent atoms:

Table S6-7. Hydrogen bonds for rt173 [A and deg.].

D-H...A	d(D-H)	d(H...A)	d(D...A)	<(DHA)
C(8)-H(8)...O(2'^b)#1	0.95	2.64	3.332(9)	130.6
C(7)-H(7)...S(5)#2	0.95	3.02	3.836(5)	145.4
C(9^a)-H(9B^a)...S(1)	0.99	2.66	3.262(10)	119.7
C(11^a)-H(11B^a)...O(2^a)#3	0.99	2.63	3.212(16)	118.0
O(2^a)-H(2O^a)...O(2^a)#4	1.04(14)	2.54(13)	3.058(15)	110(8)
C(11'^b)-H(11C^b)...O(2'^b)#3	0.99	2.52	3.151(14)	121.2
O(2'^b)-H(2O'^b)...O(2'^b)#3	0.90(15)	2.22(13)	2.899(19)	132(11)

Symmetry transformations used to generate equivalent atoms:
#1 x,-y+1,z-1/2 #2 -x+3/2,y-1/2,z #3 -x+1,y,-z+5/2
#4 -x+1,-y+1,-z+2
